# Experimental and theoretical study of the low-temperature kinetics of the reaction of CN with CH_2_O and implications for interstellar environments†

**DOI:** 10.1039/d2cp05043a

**Published:** 2023-02-24

**Authors:** Niclas A. West, Lok Hin Desmond Li, Tom J. Millar, Marie Van de Sande, Edward Rutter, Mark A. Blitz, Julia H. Lehman, Leen Decin, Dwayne E. Heard

**Affiliations:** a School of Chemistry, University of Leeds Leeds LS2 9JT UK d.e.heard@leeds.ac.uk; b Astrophysics Research Centre, School of Mathematics and Physics, Queen's University Belfast University Road Belfast BT7 1NN UK; c School of Physics and Astronomy, University of Leeds Leeds LS2 9JT UK; d Instituut voor Sterrenkunde, KU Leuven Celestijnenlaan 200D 3001 Leuven Belgium

## Abstract

Rate coefficients for the reaction of CN with CH_2_O were measured for the first time below room temperature in the range 32–103 K using a pulsed Laval nozzle apparatus together with the Pulsed Laser Photolysis–Laser-Induced Fluorescence technique. The rate coefficients exhibited a strong negative temperature dependence, reaching (4.62 ± 0.84) × 10^−11^ cm^3^ molecule^−1^ s^−1^ at 32 K, and no pressure dependence was observed at 70 K. The potential energy surface (PES) of the CN + CH_2_O reaction was calculated at the CCSD(T)/aug-cc-pVTZ//M06-2X/aug-cc-pVTZ level of theory, with the lowest energy channel to reaction characterized by the formation of a weakly-bound van der Waals complex, bound by 13.3 kJ mol^−1^, prior to two transition states with energies of −0.62 and 3.97 kJ mol^−1^, leading to the products HCN + HCO or HNC + HCO, respectively. For the formation of formyl cyanide, HCOCN, a large activation barrier of 32.9 kJ mol^−1^ was calculated. Reaction rate theory calculations were performed with the MESMER (Master Equation Solver for Multi Energy well Reactions) package on this PES to calculate rate coefficients. While this *ab initio* description provided good agreement with the low-temperature rate coefficients, it was not capable of describing the high-temperature experimental rate coefficients from the literature. However, increasing the energies and imaginary frequencies of both transition states allowed MESMER simulations of the rate coefficients to be in good agreement with data spanning 32–769 K. The mechanism for the reaction is the formation of a weakly-bound complex followed by quantum mechanical tunnelling through the small barrier to form HCN + HCO products. MESMER calculations showed that channel generating HNC is not important. MESMER simulated the rate coefficients from 4–1000 K which were used to recommend best-fit modified Arrhenius expressions for use in astrochemical modelling. The UMIST Rate12 (UDfa) model yielded no significant changes in the abundances of HCN, HNC, and HCO for a variety of environments upon inclusion of rate coefficients reported here. The main implication from this study is that the title reaction is not a primary formation route to the interstellar molecule formyl cyanide, HCOCN, as currently implemented in the KIDA astrochemical model.

## Abstract 2

Rate coefficients for the reaction of CN with CH_2_O were measured for the first time below room temperature in the range 32–103 K using a pulsed Laval nozzle apparatus together with the Pulsed Laser Photolysis–Laser-Induced Fluorescence technique.

## Graphical abstract

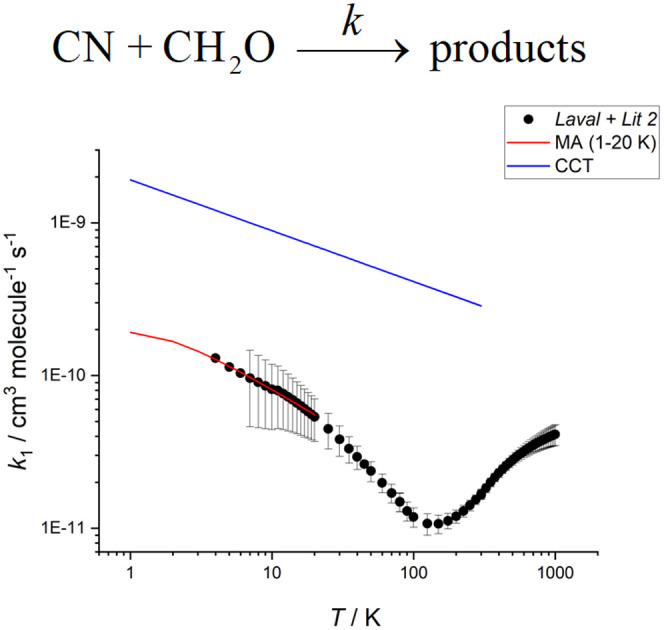

## 1 Introduction

The current list of rate coefficients and product branching ratios determined experimentally for reactions at very low temperatures, typical of astrochemical environments, is still relatively sparse. Theoretical calculations of these kinetics parameters require knowledge of the potential energy surface (PES), in particular the energy of transition states and intermediates along the reaction coordinate. At very low temperatures, only reactions with small or no activation barriers are likely to proceed quickly. Calculated rate coefficients are highly sensitive to small changes to the PES, which can be on the order of the uncertainties of the *ab initio* theory being used (estimated to be 3.0–4.5 kJ mol^−1^ at the CCSD(T)/aug-cc-pVTZ level of theory^1^), making kinetics predictions less reliable.^2^ Extrapolation of parameterisations to very low temperatures determined from rate coefficients at higher temperatures is fraught with difficulty, and has been shown to be incorrect by orders of magnitude.^3^ In some cases, this disagreement can be due to a change in the chemical mechanism which governs the temperature dependence of rate coefficients, *k*(*T*), at low temperatures.^3,4^

There is a desire to understand what aspects of the reaction potential energy landscape control the behavior of the reaction rate coefficient and the branching to products as a function of temperature. As discussed in a recent perspective,^3^ both PESs with small barriers to reaction and/or submerged barriers to reaction can cause sharp increases in rate coefficients at low temperatures until in some cases the collision limit is reached. For the case of small barriers to reaction under low temperature reaction conditions, weakly bound complexes in the entrance channel for reaction are formed with less energy compared with higher temperatures. This causes their lifetime for dissociation back to reactants to become sufficiently long that quantum mechanical tunneling through reaction barriers to products can become competitive, leading to a dramatic increase in the rate coefficient with decrease in temperature.^3^ Two examples are the reaction of OH with Complex Organic Molecules (COMs) such as CH_3_OH,^3,5–8^ and the reaction of C(^3^P) with H_2_O.^9^ For the case of submerged barriers, an increase in the rate coefficient with a decrease in temperature is observed consistent with a negative activation energy to reaction. Two examples are the reaction of C_2_H with O_2_,^10^ and the reaction of O(^1^D) with CH_4_.^11^ If a calculated PES is used in conjunction with kinetic models to predict *k*(*T*), any uncertainty in the height or width of relatively small potential energy barriers can lead to large uncertainties in the prediction of reaction rate coefficients. Therefore, these reaction-specific PES attributes are important to closely examine alongside robust experimental data spanning a broad temperature range, especially including low temperatures.

The current study focuses on the low temperature reaction of the cyano radical with formaldehyde, CN + CH_2_O (Reaction 1), which was suggested by astrochemists to be the primary formation route to the interstellar molecule formyl cyanide, HCOCN,^12^ a suggestion reinforced recently by the *ab initio* quantum calculations of the PES and master equation calculations of the rate coefficients by Tonolo *et al.*^13^ Chemical intuition and results from past studies of similar reactions^14–16^ suggest, however, that CN will likely attack CH_2_O *via* a hydrogen abstraction mechanism, forming hydrogen cyanide (HCN) and the formyl radical (HCO). Both reactants and predicted hydrogen abstraction products have already been observed in several astrochemical environments, such as Asymptotic Giant Branch (AGB) stellar winds,^17,18^ the InterStellar Medium (ISM),^19–26^ and dark clouds,^27–30^ providing further motivation for the current study at low temperatures. Additionally, the CN + CH_2_O reaction is relevant to combustion processes,^31^ planetary atmospheres,^32^ and Titan's atmosphere.^33,34^ The inclusion of new kinetic data for neutral–neutral reactions at very low temperatures can make a significant difference for the abundance of key species in interstellar environments.^3,35^

Previous experimental work on the CN + CH_2_O reaction measured overall rate coefficients between 297–673 K by Yu *et al.*^36^ and 294–769 K by Chang and Wang^37^ (for ease of reading these two works are referred to from now on as YCW^36,37^) using the Pulsed Laser Photolysis–Laser-Induced Fluorescence (PLP–LIF) technique in resistively-heated flow cells. In these studies, *k*_1_(*T*) was found to decrease slightly with decreasing temperature, from approximately 4 × 10^−11^ cm^3^ molecule^−1^ s^−1^ near 770 K to approximately 1.5 × 10^−11^ cm^3^ molecule^−1^ s^−1^ at room temperature. The uncertainties quoted for these studies were on average 2.8% for Yu *et al.*,^36^ and 1.2% for Chang and Wang.^37^ It was predicted that a hydrogen abstraction process, forming HCN, was likely to dominate the chemical mechanism. Indeed, a prior theoretical study (QCISD/6-31G**//UHF/6-31G**) found only a very small (∼2.7 kJ mol^−1^) barrier to this H atom abstraction reaction.^38^ In comparison, a very recent calculation by Tonolo *et al.*^13^ (CCSD(T)/CBS+CV) reports a small submerged barrier for the abstraction pathway forming HCN. Further aspects of the reaction potential energy surface were explored in this calculation, with particular attention paid to addition reaction pathways forming CN–CH_2_O adducts, with bonds formed between the C of formaldehyde and either end of CN. However, the high-pressure limit rate coefficient (2 × 10^−10^ cm^3^ molecule^−1^ s^−1^) calculated using transition state theory and based on this new PES^13^ is close to an order of magnitude larger than the previously measured room temperature studies of YCW. Exothermic product channels for the reaction of CN with CH_2_O include:^13,39,40^1a
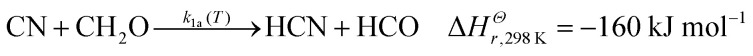
1b
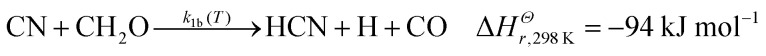
1c
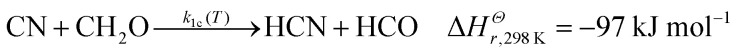
1d
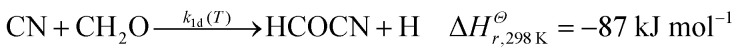
1e
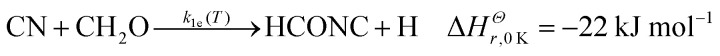


Enthalpies for reactions (1a)–(1c) are taken from Ruscic and Bross 2019;^39^ for reaction (1d) from Born *et al.*;^40^ but for reaction (1e) the zero-point energy corrected energy change calculated by Tonolo *et al.*^13^ is given. In the work presented here, low-temperature reaction rate coefficients were measured for the first time below 294 K for the CN + CH_2_O reaction using the PLP–LIF technique in a pulsed Laval nozzle apparatus. Using CCSD(T)/aug-cc-pVTZ//M06-2X/aug-cc-pVTZ, an *ab initio* PES was calculated for CN + CH_2_O and utilized in the Open Source MESMER software package^41^ to calculate temperature-dependent rate coefficients and product branching ratios. Using a fitting procedure, the parameters within MESMER were optimized to give the best agreement with experimental data over the range 32–769 K. These MESMER rate coefficients were then used to develop a parameterisation *via* a modified Arrhenius (MA) equation and extrapolated to 4 K. The fits of the rate coefficients were then incorporated into astrochemical simulations of cold dense clouds, AGB stars, and hot molecular cores.

## 2 Methods

### 2.1 Experimental measurement of rate coefficients at low temperatures

**Fig. 1:**
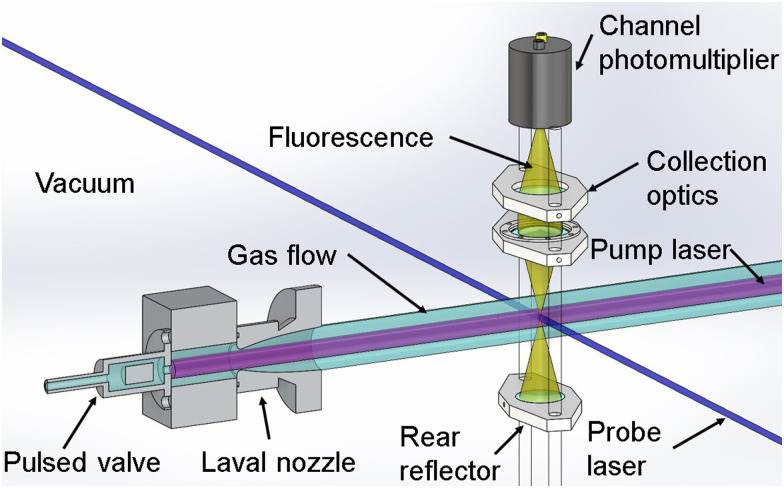
Schematic of the pulsed Laval nozzle apparatus and the PLP–LIF technique. Reprinted and adapted with permission from D. E. Heard, *AcChR*, 2018, **51**, 2620^3^. Copyright 2018 American Chemical Society.

Rate coefficient measurements for the reaction of CN with CH_2_O were performed in a pulsed Laval nozzle apparatus using the PLP–LIF technique, as shown schematically in Fig. 1.

The generation of uniform, thermalized, cold, gas flows for kinetics measurements with this apparatus was described previously in detail and will only be described briefly here.^14,42–47^ Gaseous formaldehyde (CH_2_O) is a difficult reagent to work with and particular care was given to its preparation from paraformaldehyde. Preparation of mixtures with the bath gas, its handling, and quantification of its concentration are necessary for accurate kinetics studies. Generation of the CH_2_O reagent gas was performed by controllably heating paraformaldehyde powder (Sigma-Aldrich, 95%) in an evacuated 500 mL glass bottle (Duran) with a heat gun (Steinel, model HL1810S) to ∼70 °C. During heating of paraformaldehyde, the nascent CH_2_O gas was then passed through a −10 °C cold trap. The cold trap was submerged in ethanol (VWR, 99.96%) which was chilled with a refrigerated immersion probe (LaPlant, model 100CD). The cold trap was utilized to trap any water or other condensable byproducts generated during the heating of paraformaldehyde. The purified CH_2_O was then allowed to flow into evacuated cylinders until reaching a pressure of ∼200 torr (∼26.7 kPa). The cylinders were then filled with argon (BOC, 99.998%) or nitrogen (BOC, 99.998%) to ∼6 atm (∼608 kPa) total (generating ∼4.4% mixtures of CH_2_O/bath gas). The gas in the cylinders was then allowed to mix for >12 hours. This method of generating CH_2_O is similar to methods utilized previously by our group and other groups.^14,48,49^ To generate the cyano radical, CN, the vapor from the solid precursor cyanogen iodide (ICN, Acros Organics, 98%, vapor pressure ∼1 torr (∼133 Pa) at 298 K.^50,51^) was entrained in ∼2 atm (∼203 kPa) of Ar or N_2_ bath gas.

The controlled mixing of reagent gases was accomplished through the use of Mass Flow Controllers (MFC) (MKS, type 1179A) such that final mixtures of gases were ∼0.1–1% CH_2_O, ∼0.005% ICN, and ∼99% Ar or N_2_ bath gas with a total flow rate of ∼2–5 slm. The gases were then allowed to mix in a mixing manifold prior to being pulsed through the Laval nozzle. Since CH_2_O was found to slowly repolymerize, depositing solid paraformaldehyde in the gas lines and causing the MFC calibration to slowly drift, the concentration of CH_2_O was determined *via* UV absorption measurements by sampling gas from the tubing between the mixing manifold and the pulsed valves for each concentration of CH_2_O utilized in a kinetics experiment.^14^ Measurements of the concentration of CH_2_O were made using a custom-made 1 m path length UV/Vis absorption cell filled to ∼1.2 atm (∼122 kPa) with the gas-mixture as measured by a capacitance manometer (MKS, 0–100 PSIA (∼689 kPa)). The light source for absorption measurements was a UVB lamp (EXOTERRA, UVB200) with continuous output around ∼290–350 nm. Absorption measurements were performed with a UV/Vis spectrometer (Ocean Optics, HR4000CG-UV-NIR) with 0.75 nm resolution. Absorption spectra were integrated for ∼2 seconds and 4 spectral traces were averaged. Representative averaged UV absorption spectra for CH_2_O are shown in Fig. S1 in the ESI.† Using concentrations determined from this spectrometer, a typical half-life of the generated ∼4.4% CH_2_O in an N_2_ bath in the cylinders was determined to be approximately 4 days.

After the mixing manifold, the gas mixtures were sent to two solenoid valves (Parker, Series 9) to be pulsed at 5 Hz into the pre-expansion region upstream of the Laval nozzle. Gas in the pre-expansion reservoir underwent a controlled expansion through a custom-made, axisymmetric, converging-diverging Laval nozzle such that the flow into the vacuum chamber at 0.3–2 torr (40–267 Pa) was a wall-less reactor. The vacuum chamber was continuously evacuated by two Roots blowers operating in parallel: a Roots blower (Leybold RUVAC 251) backed by a rotary pump (Leybold D65B) and a Roots blower (Edwards EH250) backed by a rotary pump (Edwards ED660) and the pressure in the vacuum chamber was monitored by a capacitance manometer (Leybold, type CTR90, 0–10 torr (0–1.3 kPa)). Flow temperatures between 32–103 K were achieved by switching between Laval nozzles of Mach numbers between 2.49 and 5.00 and/or switching between Ar and N_2_ bath gases. Impact pressure measurements using a Pitot tube were utilized to determine the Mach number along the flow, and *via* the Rayleigh equations, the density and rotational–translational temperature of the flow,^4^ as illustrated for temperature in Fig. S2 (ESI†).

In order to initiate reactions of CN + CH_2_O, the ICN precursor was photolyzed at 266 nm (∼30 mJ per pulse) using the fourth harmonic of a Nd:YAG laser (Quantel Q-Smart 850), which was directed co-linearly with the supersonic flow along the axis of the Laval nozzle (“Pump laser” in Fig. 1), generating a uniform density of CN radicals. Although the CN radical is initially generated with rotational excitation,^52–54^ collisions with the bath gas at the densities used ensure that rotational relaxation occurs on a timescale that is short compared with the timescale for removal of CN due to its reaction with CH_2_O. The time evolution of the CN radicals produced are monitored using laser-induced fluorescence following excitation of the R_1_(2) rotational line of the B^2^Σ − X^2^Σ (1,0) vibronic transition at 357.893 nm, which is generated by a Nd:YAG (Quantel, Q-smart 850) pumped dye laser (Sirah, Cobra Stretch, with Pyridine 2 dye). This “probe” laser beam (Fig. 1) crossed the gas flow perpendicularly at a fixed position ∼10–25 cm from the nozzle exit depending on the stable flow length of the particular nozzle used. Fluorescence from CN (B^2^Σ − X^2^Σ) was focused through a series of lenses and through a bandpass filter at 400 nm with a FWHM of 40 nm (Thorlabs, FB400-40) onto a Channel PhotoMultiplier (CPM) (PerkinElmer, C1952P). The gain of the CPM was controlled with a custom-built time-dependent high voltage gating module in order to block scattered light from the pump laser at 266 nm. Digitization and integration of the CPM signal were performed on an oscilloscope (LeCroy, Waverunner LT264), which is then transferred and saved to a computer for further analysis *via* a LabVIEW program. The timing of the experiment was also controlled *via* LabVIEW communication with a digital delay generator (BNC, Model 555), which randomly varied the order of the time delays between the pump and probe laser after each gas pulse.

### 2.2 Computational methods

Theoretical approaches were used in this work to calculate the PES of the CN + CH_2_O reaction in order to further explore the reaction mechanisms responsible for the behavior of the reaction rate coefficients as a function of temperature. Geometric structures of stationary points (reactants, products, intermediates, and transition states (TSs)) were first optimized at the BHandHLYP/aug-cc-pVDZ level of theory^55–58^ and further refined using M06-2X/aug-cc-pVTZ.^59^ Higher-level single-point energy calculations were performed at the CCSD(T)/aug-cc-pVTZ level^60,61^ to obtain more accurate energies. Vibrational frequency calculations were performed to evaluate zero-point vibrational energies (ZPVE), where TSs were found to have only one imaginary vibrational frequency. The vibrational frequency scaling factors for BHandHLYP/aug-cc-pVDZ and M06-2X/aug-cc-pVTZ are taken to be 0.9589 and 0.956, respectively.^62,63^ Intrinsic reaction coordinate (IRC) calculations were carried out for all TSs located during the PES search, unless otherwise specified, to verify that they are indeed saddle points on the minimum energy pathways connecting the respective local minima. In order to further explore the long-range reaction PES as the two reactants approach each other, relaxed scans were performed along the reaction entrance channels at the BHandHLYP/aug-cc-pVDZ and M06-2X/aug-cc-pVTZ levels of theory. For a relaxed scan, one of the geometric parameters is selected as the scan coordinate while all the other geometric parameters, unless being specifically frozen (in which it is referred to as a partially constrained scan), are allowed to be optimised to give the minimum energy geometry. All electronic structure calculations were carried out using the Gaussian 09 program.^64^

From the generated PES, statistical rate theory calculations were performed using the MESMER software program^41^ in order to obtain the rate coefficients of the system. The stationary points of the *ab initio* calculations provide the energies, rotational constants, and vibrational constants required by the MESMER input file. The energy wells along the PES are divided into energy grains, where each grain couples the reactant, intermediate, and product species to one another *via* the microcanonical rate coefficients, *k*(*E*). The individual grains can be populated/depopulated by exchange with other grains *via* collisional energy transfer with the buffer gas. The microcanonical rate coefficients were calculated with either Rice, Ramsperger, Kassel, and Marcus (RRKM) theory^65^ for reactions involving a defined transition state or the inverse Laplace transformation (ILT) method^66^ for barrierless reactions. Collisional energy transfer probabilities were described using the exponential-down model.^67^ Corrections for quantum mechanical tunneling were also included using the Eckart expression.^68^ The set of coupled differential equations that describe each of the energy grains is known as the energy grained master equation (EGME) and can be described by:2
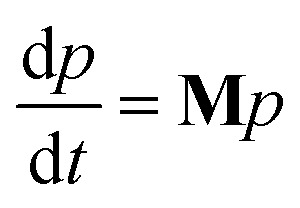
where *p* is the population density vector containing populations of each grain from each well, and **M** is the transition matrix that describes the population evolution due to collision energy transfer and reaction. The solution to eqn (2) is

3 *p* = **U**e^*Λt*^**U**^−1^*p*(0)

where *p*(0) contains the initial conditions for each grain, **U** is the matrix of eigenvectors obtained from the diagonalization of **M**, and *Λ* is the diagonal matrix of corresponding eigenvalues, where the smallest are the chemically significant eigenvalues (CSE). MESMER solves the EGME and obtains the phenomenological rate coefficients from the CSE using the procedure described by Bartis and Widom.^69^ Using the time dependence of the concentration of all species calculated by MESMER, the branching yield of different products can be determined. In addition, MESMER has a built-in fitting feature using available experimentally measured rate coefficients, whereby input parameters, *e.g.* the energies of the stationary points, can be adjusted to best fit to the experimental data. The input file for MESMER simulations used in this work are included in the online ESI.†

## 3 Results and discussion

### 3.1 Experimental results

The temperature-dependent rate coefficients for the reaction of CN + CH_2_O were obtained by first measuring the time-evolution of the relative transient LIF signal of CN as the radical reacted with an excess abundance of CH_2_O under pseudo-first-order conditions. The integrated LIF signal at each time delay between the photolysis laser (pump) and the dye laser (probe) was collected at least 5 times in order to obtain averaged traces of the temporal evolution of CN. Any background signal due to the probe-laser only was subtracted using the average value of data recorded prior to the pump-laser pulse.

The initial photolytic production of CN and fast collisional relaxation of rotationally-excited^52–54^ CN into the CN (X^2^Σ, *N* = 2) laser-probed state led to a rapid rise in the CN LIF signal (≲1 μs) which was not resolved in our experiments. Therefore, traces were analyzed after ∼5 μs following the initial rise in the signal, after which there was an exponential decay of CN due to diffusion, reaction with CH_2_O, and reaction with other possible species. Diffusion of CN out of the volume of supersonic flow through which the pump laser passed is given by:4
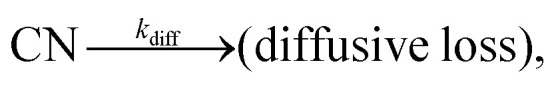


**Fig. 2:**
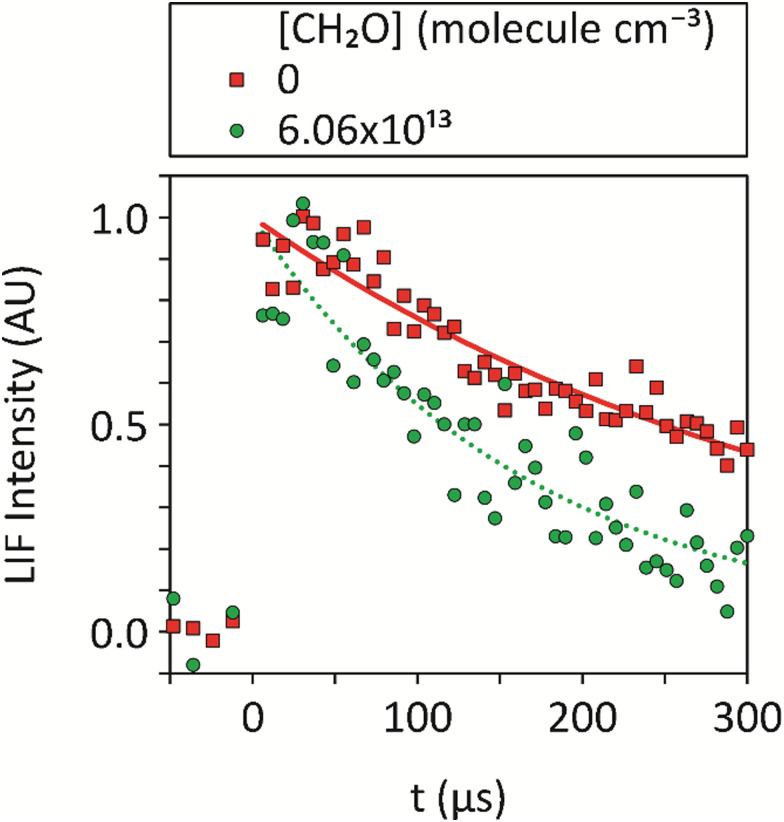
Averages of ≥5 temporal decays of the CN integrated LIF signal utilized to determine the pseudo-first-order rate coefficients, *k*_obs_, for loss of CN at 32 K, both in the absence of CH_2_O and for [CH_2_O] = 6.06 × 10^13^ molecule cm^−3^, at a total density of (3.24 ± 0.24) × 10^16^ molecule cm^−3^ in Ar bath gas, together with exponential fits (eqn (7)) to the data. No data could be recorded for longer pump-probe time delays due to the limited reaction time within the low-temperature uniform supersonic flow prior to the breakup of the flow.

The reaction of CN with CH_2_O (reaction 1, bimolecular rate coefficient *k*_1_), and potential reactions with other species (for example, with the precursor, other photolysis products, or reagent impurities) is given by:5
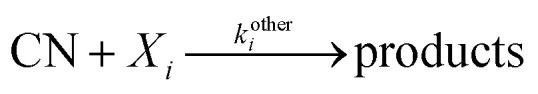
where *X*_*i*_ represents other non-reagent species *i*. The observed pseudo-first-order rate coefficient for the loss of CN, *k*_obs_, is thus given by:6
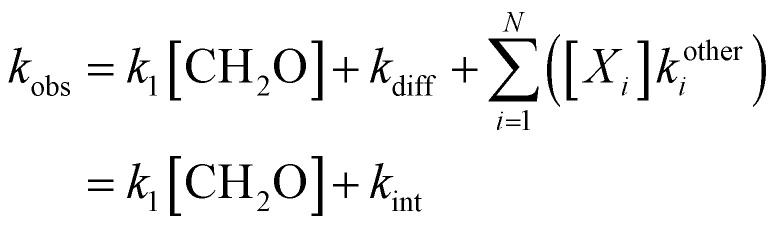
where *N* is the total number of species other than CH_2_O in the cold gas flow and *k*_int_ represents the intercept of a linear fit of a plot of *k*_obs_*versus* [CH_2_O], with *k*_int_ expected to be dominated by *k*_diff_. Each CN integrated LIF decay trace, *I*(*t*), which is proportional to [CN]_*t*_, was fitted with a single exponential decay function:7
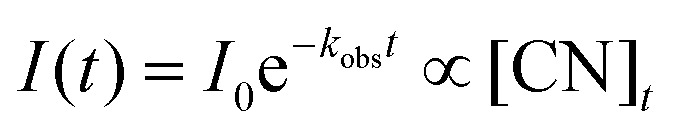
8
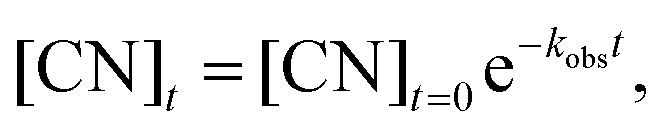
where *t* is the pump–probe time delay, and *I*_0_ is the fitted value of LIF signal at *t* = 0 for exponential decay fits of the signal. Fits of eqn (7) to the data were performed starting at a pump–probe time delay of ∼5 μs. This ensured the completion of all significant relaxation of CN into the rovibronic state that was being probed that could otherwise interfere with the analysis. Examples of the background corrected, averaged, temporal evolution of the CN integrated LIF signal together with such fits are shown in Fig. 2.

**Fig. 3:**
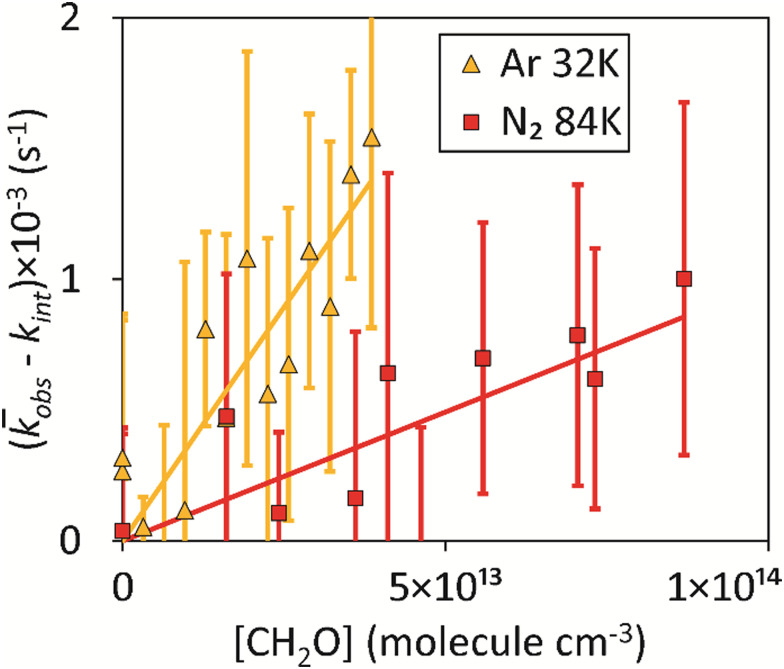
Variation of the intercept-subtracted, average loss rate of CN, *k̄*_obs_ − *k*_int_, with [CH_2_O], together with linear-least squares fits of eqn (6) to the data for *T* = 32 K, total density (Ar bath gas) = (3.24 ± 0.24) × 10^16^ molecule cm^−3^, and *T* = 84 K, total density (N_2_ bath gas) = (7.56 ± 0.63) × 10^16^ molecule cm^−3^. Error bars represent one standard deviation of the value of *k̄*_obs_ obtained from at least 5 temporal decays of CN (Fig. 2).

For each CH_2_O concentration, the experiment was repeated at least five times and the *k*_obs_ values obtained were averaged to give *k̄*_obs_. Second-order (bimolecular) plots of *k̄*_obs_*versus* [CH_2_O] were then generated at each temperature, examples of which are given in Fig. 3 (with the intercept *k*_int_ subtracted for clarity) with a linear least-squares fit of eqn (6) used to determine *k*_1_ from the gradient.

For the linear fits of the second-order plots such as shown in Fig. 3, care was taken to include only the range of [CH_2_O] for which *k̄*_obs_ is linear with [CH_2_O] to avoid any possible influence from CH_2_O dimers. Evidence for dimerization of CH_2_O was found in previous low-temperature studies at higher [CH_2_O] performed using a Laval nozzle, namely for rate coefficient determinations of the reactions OH + CH_2_O,^15^ CH + CH_2_O,^14^ and NH_2_ + CH_2_O.^70^ The range of values of the intercept (at [CH_2_O] = 0 molecule cm^−3^) was *k*_int_ = 2200–7600 s^−1^, and the variation of *k̄*_obs_ with [CH_2_O] without the subtraction of *k*_int_ can be found in Fig. S3 of the ESI.† We have been very cautious in the maximum [CH_2_O] used to obtain *k*_obs_ for a given temperature, always using less than the [CH_2_O] at which second-order plots have been seen to become non-linear in similar studies of radical + CH_2_O reactions.^14,15^ The values of *k*_1_(*T*) determined in this work for the CN + CH_2_O reaction, together with corresponding experimental conditions, are given in Table 1. At 70 K, *k*_1_ was determined at two overall densities providing evidence that *k*_1_ is independent of pressure.

**Table 1:** Rate coefficients and experimental conditions for kinetics studies of the CN + CH_2_O reaction

*T* a (K)	Bath gas	*N*_total_ a (10^16^ molecule cm^−3^)	*k*_1_(*T*)^b^ (10^−11^ molecule^−1^ cm^3^ s^−1^)
32 ± 2	Ar	3.24 ± 0.24	3.57 ± 0.53
32 ± 2	Ar	3.24 ± 0.24	4.62 ± 0.84
40 ± 4	Ar	8.36 ± 1.19	2.26 ± 2.11
53 ± 4	Ar	7.04 ± 0.74	3.18 ± 1.19
56 ± 6	Ar	7.58 ± 1.11	1.56 ± 1.94
70 ± 11	Ar	11.18 ± 2.54	1.51 ± 0.49
70 ± 2	N_2_	2.91 ± 0.20	1.30 ± 0.52
84 ± 3	N_2_	7.56 ± 0.63	0.99 ± 0.27
92 ± 6	N_2_	4.99 ± 0.74	1.80 ± 0.29
103 ± 10	N_2_	6.80 ± 1.57	1.45 ± 0.19

a Pitot tube measurements of impact pressures were utilized to determine *T* and *N*_total_. Here 1*σ* fluctuations of these values in the cold flow along the axis of the nozzle are reported.

b The error of each *k*_1_(*T*) value represents the standard error in the fitted value of the gradient of *k̄*_obs_*versus* [CH_2_O].

**Fig. 4:**
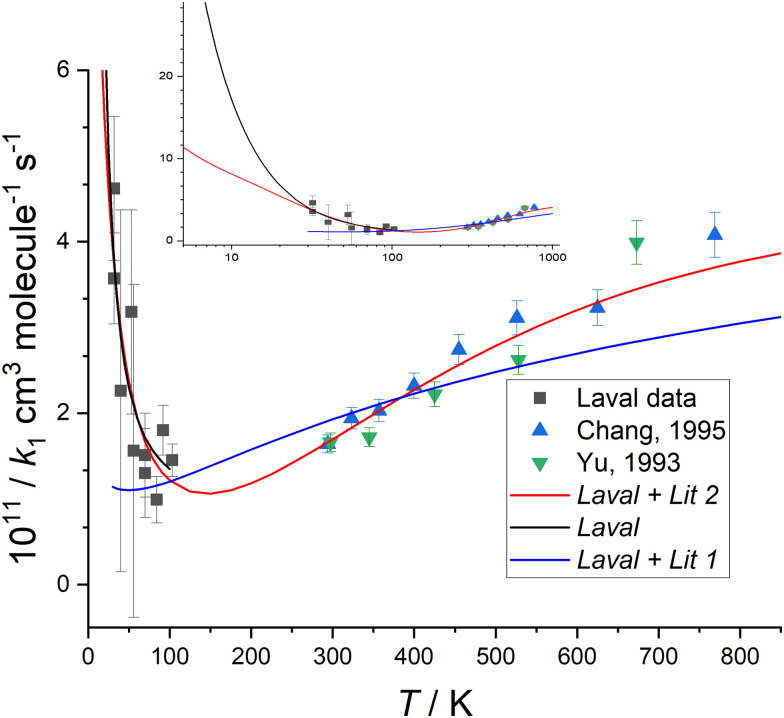
Measured values of *k*_1_(*T*) *versus* temperature from this work (black squares), Chang and Wang^37^ (blue triangles), and Yu *et al.*^36^ (green triangles) (collectively YCW). The error bar of each *k*_1_(*T*) from this work are those given in Table 1, and the YCW values are assigned an error equal to 0.064 × *k*_1_(*T*), see text for details. The Laval fit (black line) are the MESMER simulations which are a best-fit to the experimental Laval measurements only, with *ab initio* parameters (Table 2) including a submerged transition state. For details of the Laval + Lit 1 and Laval + Lit 2 fitting scenarios and parameters see Table 2. The inset shows the extrapolation of Laval (black line) and Laval + Lit 2 (red line) models to lower temperatures, where significant deviation in these models is only observed below 30 K. See text and Table 2 for further details of these scenarios.

The measurement uncertainty for *k*_1_ for CN + CH_2_O given in Table 1 is larger than reported in our determination of the rate coefficient for the CH + CH_2_O reaction.^14^ The uncertainty quoted is a statistical error only from linear fits of the type shown in Fig. 3, and adding systematic errors did not increase the overall error significantly. The primary reason is that *k*_1_(*T*) is about 10–100 times smaller than *k*_CH+CH_2_O_(*T*) across the range of temperatures studied (0.99 × 10^−11^–4.62 × 10^−11^*versus* 4.86 × 10^−10^ – 11.15 × 10^−10^ cm^3^ molecule^−1^ s^−1^, respectively). Hence, for the same [CH_2_O] and available reaction time (controlled by the length of the stable flow for a given Laval nozzle), the LIF signal from CN does not decay as much as for CH, resulting in a relatively larger uncertainty in the fit of eqn (7) to the data to determine *k*_obs_. The values of *k*_1_(*T*) determined in this work for *T* = 32–103 K for the CN + CH_2_O reaction are shown in Fig. 4, and also in Fig. S8(a) and S10 of the ESI,† together with experimental measurements from two other groups^36,37^ reported over the temperature range 294–769 K.

Fig. 4 shows that *k*_1_(*T*) exhibits a weak positive temperature dependence above room temperature but a strong negative temperature dependence below ∼100 K, with a likely minimum somewhere between 100–200 K, suggestive of a change in reaction mechanism between the low and high temperature regimes. The values of *k*_1_(*T*) at low temperatures are less precise than those previously reported at higher temperatures,^36,37^ due to the challenges for this experiment as discussed above. It is noted though that for the previous data reported at higher temperatures, the absolute concentration of CH_2_O was not determined by UV absorption spectroscopy (in contrast to the low temperature work reported here), rather manometric/flow methods were used to determine [CH_2_O].

### 3.2 *Ab initio* calculations of the CN + CH_2_O potential energy surface

**Fig. 5:**
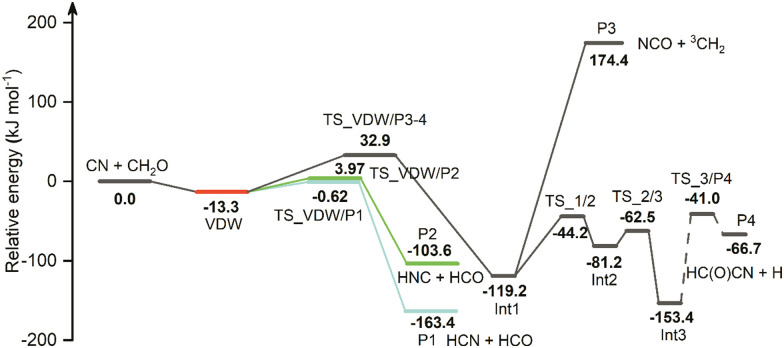
PES of the CN + CH_2_O reaction obtained at the CCSD(T)/aug-cc-pVTZ//M06-2X/aug-cc-pVTZ level of theory. All energy values are in kJ mol^−1^ and are corrected with scaled ZPVE. The red line indicates the van der Waals complex VDW found in this work. The blue and green paths show the subsequent pathways from VDW leading to HCN + HCO (P1) and HNC + HCO (P2) products, respectively.

The overall potential energy surface for the CN + CH_2_O reaction is shown in Fig. 5, with energies obtained using CCSD(T)/aug-cc-pVTZ//M06-2X/aug-cc-pVTZ and shown relative to the CN + CH_2_O entrance channel.

The geometries of the stationary points, obtained at the M06-2X/aug-cc-pVTZ level of theory, are shown in Fig. S4 (ESI†). The optimized Cartesian coordinates, vibrational frequencies, energy values, and additional scans along the reaction potential energy surface can be found in the ESI.†

A weakly bound van der Waals complex VDW (−13.3 kJ mol^−1^) is identified following the approach of CN to CH_2_O. As seen in Fig. 5, this complex leads to four different product pathways, namely H atom abstraction to form HCN (P1), H atom abstraction to form HNC (P2), and addition of CN onto the O atom (P3–4) eventually forming either NCO + ^3^CH_2_ (P3) or HC(O)CN + H (P4). The formation of HCN + HCO (P1, −163.4 kJ mol^−1^) is accessible through the submerged barrier TS_VDW/P1 (−0.62 kJ mol^−1^) while the formation of HNC + HCO (P2, −103.6 kJ mol^−1^) involves a small positive energy barrier relative to the CN + CH_2_O entrance channel (TS_VDW/P2, 3.97 kJ mol^−1^). The error of these two calculated barrier heights, even with the high level of theory used here, are such that they could both be either positive or submerged barriers if calculated at a different level of theory. At the CCSD(T)/aug-cc-pVTZ level of theory, the error of the energy is estimated to be 3.0–4.5 kJ mol^−1^ (250–450 cm^−1^).^1^

The addition of CN onto the O atom of CH_2_O involves surmounting a large barrier TS_VDW/P3-4 (32.9 kJ mol^−1^), leading to the formation of the intermediate H_2_C–O–CN (Int1, −119.2 kJ mol^−1^). H_2_C–O–CN can dissociate to form NCO + ^3^CH_2_ (P3, 174.4 kJ mol^−1^) or undergo cyclization through TS_1/2 (−44.2 kJ mol^−1^) to form the cyclic intermediate Int2 (−81.2 kJ mol^−1^). By going through TS_2/3 (−62.5 kJ mol^−1^), the ring opens to form the intermediate H_2_C(O)CN (Int3, −153.4 kJ mol^−1^). Breaking one of the CH bonds gives the products HC(O)CN + H (P4, −66.7 kJ mol^−1^). Although the IRC calculation did not converge successfully for TS_3/P4 (−41.0 kJ mol^−1^), judging from the vibrational mode of the imaginary frequency, it is likely that it is the TS connecting Int3 and P4. A dashed line connecting TS_3/P4 reflects the incomplete mapping of the IRC along this coordinate.

Relaxed scans at the BHandHLYP/aug-cc-pVDZ and M06-2X/aug-cc-pVTZ level of theory attempted to map out the approach of the two reactants, CN and CH_2_O. Two different reactant approaches were investigated: the CN radical approaching from the oxygen side of CH_2_O, and CN approaching from the hydrogen side. For each scan, the distance between the two reactants was fixed while all other coordinates were allowed to optimize. When CN approaches from the oxygen side of CH_2_O, as shown in Fig. S5 (ESI†), it is favorable for CN to orient the carbon towards CH_2_O at the beginning of the approach due to the dipole–dipole attraction. The potential energy decreases smoothly as the distance between CN carbon and CH_2_O oxygen decreases, eventually reaching the potential energy well where the van der Waals structure VDW is located.

**Fig. 6:**
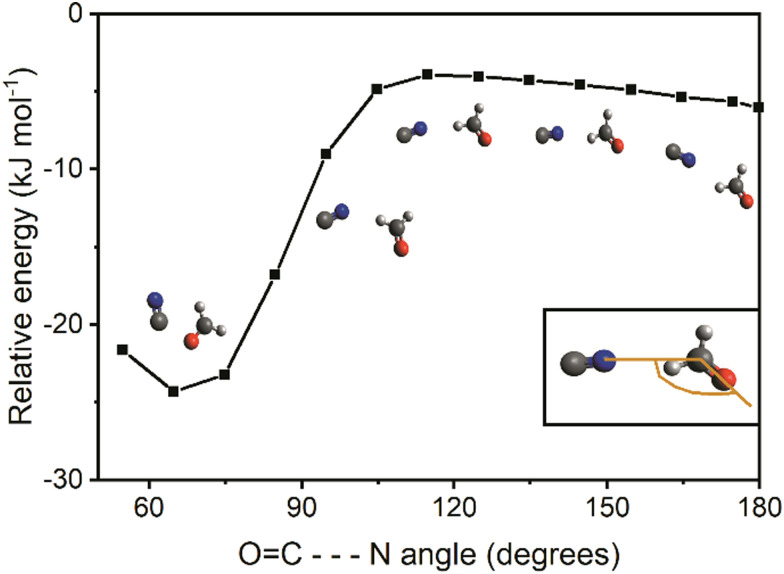
Potential energy curve (ZPVE corrected) calculated at the M06-2X/aug-cc-pVTZ level for the rotation of CN around CH_2_O for O=C–N angle from 55° to 180°. Energy is relative to the sum of the energies (ZPVE corrected) of the two separate reacting species, CN and CH_2_O, at the M06-2X/aug-cc-pVTZ level.

In the case where CN approaches from the hydrogen side of CH_2_O, as shown in Fig. S6 (ESI†), it is favorable at first for CN to orient itself such that the nitrogen side points towards CH_2_O. The potential energy decreases with decreasing separation between the two moieties until encountering a fairly “flat” region of the potential energy surface. Here, with a distance of approximately 3.3 Å between nitrogen of CN and carbon of CH_2_O, or a center of mass distance between the moieties of approximately 4.5 Å apart, the overall energy of the system (BHandHLYP/aug-cc-pVTZ, uncorrected for ZPVE) reaches −5 kJ mol^−1^ relative to the entrance channel. In order to further explore this “flat” region of the PES, a relaxed scan from this point has been done using the O=C⋯N angle as the scanning parameter, as shown in Fig. 6.

While the CN radical rotates around CH_2_O, the potential energy first experiences a fairly flat region of the potential and then falls smoothly into the potential energy well corresponding to the van der Waals structure VDW. Thus, it is suggested that both ways of approach can eventually lead to the van der Waals structure VDW.

A direct H abstraction mechanism of CN from CH_2_O to form HCN + HCO was studied in a previous calculation using the QCISD/6-31G**//UHF/6-31G** level of theory.^38^ This prior work suggested that the reaction mechanism involved overcoming a small positive ∼2 kJ mol^−1^ (emerged) barrier relative to the CN + CH_2_O entrance channel. The current study and a very recent study^13^ have not identified this pathway on the reaction coordinate, perhaps because of the lower level of theory used for geometry optimization (UHF/6-31G**) in the previous study.^13^ In the current study, instead of direct abstraction, an indirect channel to form HCN + HCO was found which involves the van der Waals complex VDW and a small submerged barrier TS_VDW/P1. A very recent theoretical study^13^ by Tonolo *et al.* also identified the VDW structure on the reaction PES (−13.0 kJ mol^−1^ relative to reactants, including ZPE, CCSD(T)/CBS + CV) leading to hydrogen abstraction to form HCN + HCO through a submerged barrier (−1.26 kJ mol^−1^). This pathway is consistent with our current work, with less than 0.5 kJ mol^−1^ difference in energy in the complex and barrier. Our work also details for the first time the presence of the HNC pathway from the VDW complex.

The work from Tonolo *et al.* identifies several stationary points along the reaction coordinate not used in the current study. For example, Tonolo *et al.* claim a barrierless route to forming a C–C bond directly from the reactants, resulting in a tetrahedral intermediate which they label 1C in a deep potential well (−153 kJ mol^−1^). Although we agree that this structure can be formed (Int3 in Fig. 5), we were unable to connect this structure to the reactants (relaxed scans, BHandHLYP/aug-cc-pVDZ and M06-2X/aug-cc-pVTZ) without surmounting a large barrier at least 50 kJ mol^−1^ (CCSD(T)/aug-cc-pVTZ//M06-2X/aug-cc-pVTZ) higher in energy than the reactants, as shown in Fig. S7 (ESI†). It is likely that the overestimated rate coefficients from Tonolo *et al.* compared to previous and current experimental work are directly related to not identifying a substantial barrier to C–C bond formation in forming their intermediate 1C. This structure 1C and the remainder of the addition pathways Tonolo *et al.* identified coming from this structure, including roaming mechanism pathways, were not considered in our search for reactions which would occur at temperatures relevant to the current study. This impacts on the relative importance stated for the pathway leading to the formation of formyl cyanide (HCOCN), although we identified a new pathway to HC(O)CN + H through VDW which does not involve roaming and has a barrier (TS_VDW/P3-4).

In comparison with the PES of OH + CH_2_O, which has been studied extensively in previous work,^2,16,71–75^ the results from the current work on CN + CH_2_O show that both systems share a similar shape of the PES in terms of the energy profile or mechanism for the H abstraction reaction. Following the approach of the two reacting species, a pre-reaction complex is formed followed by a transition state with a small barrier to form products. The difference in energy which determines whether the barrier is positive or submerged relative to the reactants energies is in the kJ mol^−1^ range, and hence within the uncertainty of most calculation methods, and so whether the transition state is submerged or not will depend on the level of theory used.

For example, the energy values of the transition state for H abstraction channel of OH + CH_2_O computed with more robust methods tend to give lower values,^2^ decreasing the barrier from being slightly emerged to slightly submerged. The latest value reported from Machado *et al.*^2^ obtained at CCSD(T)/CBS level is approximately −5.7 kJ mol^−1^.

### 3.3 Calculation of rate coefficients using the MESMER package

The energy values, see Fig. 5 (and given in Tables S1–S3, ESI†), Cartesian coordinates of the stationary points (Tables S4 and S5, ESI†), vibrational frequencies (Tables S6 and S7, ESI†), and rotational constants (Tables S8 and S9, ESI†) of the species obtained from the *ab initio* calculations were used as the inputs for the master equation solver, MESMER,^41^ in order to calculate the rate coefficients for CN + CH_2_O. From simulations over a wide range of temperatures (4–1000 K) and pressures (10^15^–10^19^ molecule cm^−3^) it was observed that HCN and HNC accounted for greater than 99.99% of the products, under all conditions. The reaction occurs initially *via* van der Waals complex (VDW) formation followed by transition states TS_VDW/P1 and TS_VDW/P2 to form HCN + HCO and HNC + HCO, respectively. The HNC channel never accounts for more than 1% yield, as shown in Fig. S8(b) (ESI†). However, uncertainties on calculated energy barriers (estimated to be 3.0–4.5 kJ mol^−1^ at the CCSD(T)/aug-cc-pVTZ level of theory^1^) can result in a reverse situation for which TS_VDW/P2 (Fig. 5) can be slightly submerged and TS/VDW/P1 somewhat emerged, and so there is a possibility that the yield of HNC may be significantly larger than this, and the yield of HCN correspondingly smaller.

As CN + CH_2_O did not show a pressure dependence for *k*_1_(*T*), either experimentally near 10^17^ molecule cm^−3^ or from MESMER simulations between 10^15^–10^18^ molecule cm^−3^, the gas density was set at 10^13^ molecule cm^−3^ (so making sure in a pressure independent region) for the MESMER simulations. Above 10^19^ molecule cm^−3^ and *T* < 50 K, a pressure dependence was evident and the VDW species was populated. No pressure dependence was observed in the current calculations, implying that the van der Waals complex is not significantly stabilised, even at the lowest temperatures.

**Fig. 7:**
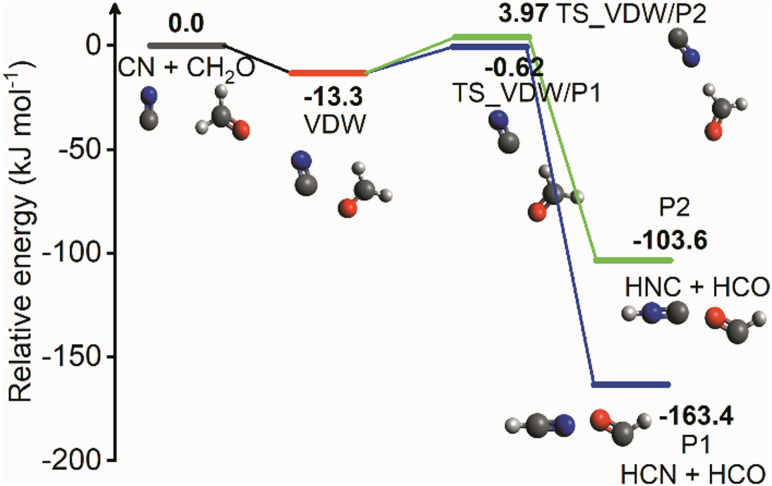
A simplified PES used for MESMER fitting scenarios, see Table 2, together with molecular structures at key stationary points. Initial van der Waals complex, VDW red, formation is followed by transition states to products, HCN + HCO (P1, blue) and HNC + HCO (P2, green) respectively. See text for details.

Therefore, to a very good approximation, Fig. 5 can be reduced to just the formation of HCN and HNC as shown in Fig. 7 without losing chemical information; reducing the system to just HCN formation would still describe the system to better than 99% of the product yield.

The inverse Laplace transformation (ILT) method^66^ was used to calculate the microcannonical rate coefficients for the barrierless formation of the VDW complex from CN + CH_2_O. The ILT approach overcomes the problem of explicitly assigning a transition state for a barrierless process, whose position would be varying as a function of temperature. The ILT method is especially convenient when experimental data are available, as for this study. The ILT parameters for VDW formation were assigned by:9
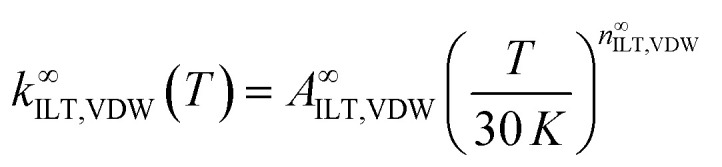
where *A*^∞^_ILT,VDW_ and *n*^∞^_ILT,VDW_ are the ILT parameters which describe the rate coefficient for the formation of the VDW at the high pressure limit (in cm^3^ molecule^−1^ s^−1^ and unitless respectively). A reference temperature in the expression of 30 K was chosen so that the *A* factor is representative of the lowest experimental data point at 32 K, making the ILT more suited with the low temperature regime relevant to conditions in the ISM. The MESMER input file for these calculations is given at the end of the ESI.†

As well as performing simulations, MESMER can adjust the important rate controlling parameters in eqn (9), as well as the transition state energies in order to best fit to available experimental data. During such data fitting, MESMER uses the Marquardt algorithm^76^ to adjust the parameters in order to minimize *χ*^2^:10
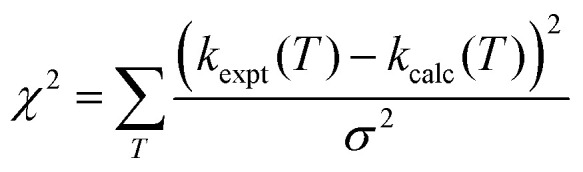
where *k*_calc_(*T*) is the MESMER calculated rate coefficient, and *k*_expt_(*T*) and *σ* are the experimental rate coefficients and their associated error. For the MESMER fitting exercise, the present low temperature results used the experimental errors given in Table 1, whereas the room temperature and above rate coefficients from YCW^36,37^ (denoted Laval + Lit 1 in Table 2) were assigned an error of 0.064 × *k*_1_(*T*). The MESMER fitting scenarios used in this work and the optimised kinetic parameters are summarised in Table 2.

**Table 2:** Fitting scenarios and optimized kinetics parameters from MESMER fitting

Name of fitting scenario	Laval	Laval + Lit 1	Laval + Lit 2
Temperature range (K)	32–103	32–769	32–769
*A* ^∞^_ILT,VDW_ ×10^−11 ^a	5.29 ± 0.8	2.5 ± 0.8	6.58 ± 0.7
*n* ^∞^_ILT,VDW_ (unitless)	−0.77 ± 0.17	0.016 ± 0.005	−0.10 ± 0.02
TS_VDW/P1 (kJ mol^−1^)	−0.62, fixed	−0.62, fixed	4.0 ± 0.9
Imaginary frequency^b^ (cm^−1^)	215, fixed	215, fixed	806 ± 24
*χ* ^2^/*N*^c^	1.09	4.24	0.80

a Units are cm^3^ molecule^−1^ s^−1^.

b Units are cm^−1^.

c *N* is the number of degrees of freedom, the number of data points minus the number of fitting parameters. The reported errors are 1 sigma.

Initially, MESMER data fitting was carried out with energies fixed at the *ab initio* calculated values, as shown in Fig. 7, with only the ILT parameters in eqn (9) being adjusted. When fitting to just the low temperature Laval nozzle data (Laval scenario in Table 2) an excellent visual fit with the data was obtained (see Fig. 4). *χ*^2^/*N* is a measure of the goodness of fit, where *χ*^2^ is given by eqn (10) and *N* is the number of degrees of freedom, which is equal to the number of data points minus the number of fitting parameters. A *χ*^2^/*N* value close to 1.0 represents a good fit, and from Table 2 it can be seen that the Laval model is close to 1.0, and indicates the experimental errors in Table 1 are realistic.

When MESMER simultaneously fits to the low and high-temperature data of YCW^36,37^ (denoted Laval + Lit 1 scenario in Table 2), the fit to the data is much worse, with *χ*^2^/*N* = 4.24, and as can clearly be seen in Fig. 4. Therefore it is concluded that MESMER calculations based on our *ab initio* calculated values is not capable of reproducing all the experimental data shown in Fig. 4. The reason is because the *ab initio* value for the energy of the transition state (TS_VDW/P1) is submerged, *i.e.* is negative with respect to the reagent energies, and a reaction mechanism proceeding *via* a submerged transition state is going to predict *k*_1_ to decrease with increasing temperature, which is at odds with the high temperature literature data, which requires the transition state to have a positive energy with respect to the reagents. The simplest way to account for the variation of *k*_1_ across the full range of temperatures is to increase the transition state energy so that it is positive, making it consistent with the high-temperature literature data trends and to increase the imaginary frequency of the tunnelling coordinate in the transition state so that quantum mechanical tunnelling overcomes the effect of the positive barrier, leading to an increase in *k*_1_ at lower temperatures, as is observed experimentally. When the model scenario Laval + Lit 2 (Table 2), where both the energy and the imaginary frequency of both transition states for the channels producing HCN and HNC products (TS_VDW/P1 and TS_VDW/P2, respectively in Fig. 7) are adjusted in the MESMER simulations using a best-fit procedure, the *k*_1_ experimental data across the full range of temperatures can be fitted well, evidenced by a value of *χ*^2^/*N* = 0.80. The optimum MESMER adjustments from the fitting were an increase in the energy from −0.62 to + 4.0 kJ mol^−1^ and an increase in the imaginary frequency from 215 to 806 cm^−1^ for TS_VDW/P1. We stress that the 4.59 kJ mol^−1^*ab initio* energy difference between the two transition states leading to HCN and HNC products (TS_VDW/P1 and TS_VDW/P2 in Fig. 7, respectively) is maintained, with the energy of the transition state for HNC (TS_VDW/P2) now being +8.59 kJ mol^−1^, and the imaginary frequency for the transition-state leading to HNC was also increased to ∼806 cm^−1^. This is reasonable given the similar calculated geometries of the two transition states, but with just the C and N flipped, as shown in Fig. S4 (ESI†), and the tunnelling motion is therefore roughly the same.

The MESMER simulation model scenario Laval + Lit 2 of the fit to the data is shown in Fig. 4, and is much improved over Laval + Lit 1. In order for the Laval + Lit 2 scenario to give the best fit to the *k*_1_ data, the transition state energy needed to be increased by ∼4.6 kJ mol^−1^ (from −0.62 kJ mol^−1^ to +4.0 kJ mol^−1^ for TS_VDW/P1, which is still the TS *via* which the reaction occurs to form HCN products) from our *ab initio* calculated values at the CCSD(T)/aug-cc-pVTZ level of theory.^1^ However, this adjustment seems reasonable given it is close to the estimated uncertainty at the CCSD(T)/aug-cc-pVTZ level of theory of ∼3.0–4.5 kJ mol^−1^.^1^ Similarly, even though the imaginary frequencies has been increased significantly, from 205 cm^−1^ to 806 cm^−1^, the adjusted value of ∼800 cm^−1^ again is not unreasonable.^5^ As both transition states emanate from the same pre-reaction complex leading to H abstraction products, it seems reasonable to adjust the energies of each of these by the same amount whilst maintaining the same difference between them. The motivation for changing the initial *ab initio* results to the fitted results using MESMER is to show that the experimental data *can* be fitted well if the potential energy surface, specifically for these two transition states, is modified. Just changing the properties of the two initially formed transition states which are formed from the same pre-reaction complex is the most straightforward way to do this.

From Fig. 4 it can be seen that the minimum in the rate coefficient *k*_1_ occurs around 150 K, which is the point at which the controlling influences of quantum mechanical tunnelling and the 4.0 kJ mol^−1^ barrier for TS_VDW/P1 become balanced. Also included in Fig. 4 is the simulation of model Laval down to 7 K, where at 10 K the predicted value of *k*_1_ is approximately twice that of the Laval + Lit 2 model. This significant difference emphasizes how sensitive *k*_1_ is to the parameters used in the models when extrapolating down to very low temperatures, *i.e.* <10 K, as both the Laval and Laval + Lit 2 models give almost the same quality fits to the experimentally measured *k*_1_ using the Laval nozzle.

### 3.4 Parameterisation of the temperature dependent rate coefficient *k*_1_(*T*) for use in astrochemical modelling

In order to provide an analytical expression for the results of the master equation analysis which is suitable for use in astrochemical modelling, the best-fit parameters of the Laval and Laval + Lit 2 scenarios (see Table 2) were used as input for MESMER simulations to generate values of *k*_1_(*T*) over a very wide temperature range from 4–1000 K, as tabulated in Table S10 (ESI†). Fig S8(a) (ESI†) shows all of the experimental data for *k*_1_(*T*) together with the MESMER simulations using the Laval + Lit 2 model. As CN + CH_2_O did not show a pressure dependence for *k*_1_(*T*), neither experimentally near 10^17^ molecule cm^−3^ nor from MESMER simulations between 10^15^–10^18^ molecule cm^−3^, the gas density was set at 10^13^ molecule cm^−3^ for the MESMER simulations; above 10^19^ molecule cm^−3^ and *T* < 50 K a pressure dependence was evident and the VDW species begins to become stabilized. From 50–1000 K, a 30 cm^−1^ grain size was sufficient to calculate a converged rate coefficient, but it was reduced down to 2 cm^−1^ for the 4–50 K simulations. It was not possible to run MESMER simulations at lower temperatures. Over the range of 4–1000 K, the MESMER simulations indicate that HCN + HCO are the only significant products formed. The fractional yield of HNC is less than 0.33% for all temperatures between 4–1000 K, because the energy of TS_VDW/P2 (forming HNC) is 4.59 kJ mol^−1^ above that of TS_VDW/P1 (forming HCN), and the imaginary frequencies of the both transition states is 806 cm^−1^. The temperature dependence of the fractional yield of the HCN and HNC products is shown in Fig. S8(b) (ESI†). The results of *k*_1_ for this simulation (Table S10 in the ESI†) are the basis for our recommended values for the overall CN + CH_2_O rate coefficient, from which parameterisations are developed for use in astrochemical models.

The equation utilized commonly as a parameterisation in several astrochemical models (which use, for example, the UMIST Rate12 (UDfa, https://udfa.ajmarkwick.net) or KIDA (https://kida.obs.u-bordeaux1.fr^78^) databases) to represent *k*(*T*) is a single modified Arrhenius (MA) equation:11
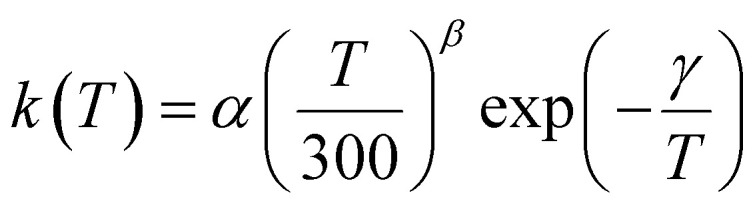
where *α*, *β* and *γ* are best-fit parameters. Although Table S10 (ESI†) can be used to look up the MESMER simulated values of *k*_1_(*T*) at a given temperature in the range 4–1000 K, a parameterisation in the form of eqn (11) is convenient to calculate *k*_1_(*T*) using an expression traditionally used by astrochemical modellers. However, a single MA eqn (11) is not able to adequately fit *k*_1_(*T*) for CN + CH_2_O over the full range of temperatures 4–1000 K. It is especially inadequate for the *k*_1_ values around 150 K, where the *T* dependence changes from negative to positive, see Fig. 4, and also at the lowest temperatures where *k*_1_ is rapidly increasing. Running MESMER simulations down to the lowest temperatures means that no extrapolation of fits using eqn (11) is necessary and avoids the extra error associated with this. However, given the use of the single MA in astrochemical databases, a piecewise parameterisation using eqn (11) is presented here. Our temperature ranges were chosen for the piecewise least-squares fits of MA eqn (11) to the MESMER simulated values of *k*_1_(*T*), and Fig. S10, ESI† shows these fits to the Laval + Lit 2 scenario for low (4–20 K), (20–100 K), (100–300 K) and (300–1000 K) temperature ranges. In addition, a fit to the Laval scenario for the low (3–80 K) temperature range was carried out and is shown in Fig. S10 (ESI†).

In the parameterization given by eqn (11) each MESMER data point was assigned a 5% error. The values for the best-fit parameters from eqn (11) fits to the Laval + Lit 2 simulation data are given in Table 3. From Table 3, it is noted that *χ*^2^/*N* is always less than 1, which implies that on average this representation is better or within 5% of the MESMER simulation data, see Fig. S10 (ESI†). Also included in Table 3 are the parameterization values for the Laval model for the range *T* = 3–80 K. But it is the parameterization of the Laval + Lit 2 model that represents our recommendation of *k*_1_(*T*) for use in astrochemical modelling.

**Table 3:** Best-fit parameters from a single modified Arrhenius equation (eqn (11)) fitted to MESMER simulated data over various temperature ranges

*T* range (K)	*α* (cm^3^ molecule^−1^ s^−1^)	*β* (Unitless)	*γ* (K)	*χ* ^2^/*N*^b^	MESMER fitting scenario
3–80^a^	(3.05 ± 0.14) × 10^−12^	−1.11 ± 0.02	−2.18 ± 0.23	0.70	Laval
4–20	(1.18 ± 0.08) × 10^−11^	−0.58 ± 0.03	0.53 ± 0.24	0.55	Laval + Lit 2
20–100	(3.72 ± 0.15) × 10^−12^	−1.09 ± 0.04	5.2 ± 1.8	0.12	Laval + Lit 2
100–300	(5.99 ± 0.14) × 10^−11^	−2.19 ± 0.04	−313.4 ± 7.7	0.11	Laval + Lit 2
300–1000	(6.26 ± 0.30) × 10^−11^	−0.02 ± 0.03	398 ± 16	0.03	Laval + Lit 2

a We recommend using the Laval + Lit 2 fitting scenario for astrochemical modelling.

b *N* is the number of degrees of freedom, which is equal to the number of MESMER simulated data points minus the number of fitting parameters.

**Fig. 8:**
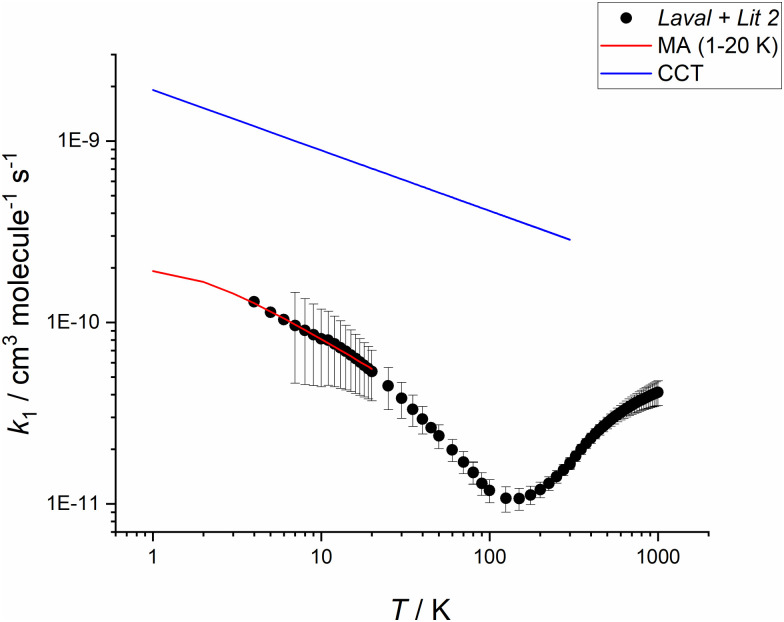
Plot of the classical capture theory (CCT) rate coefficient (blue curve) for the CN + CH_2_O reaction *versus* the *k*_1_(*T*) from the Laval + Lit 2 model (black filled circles), see Table S10 (ESI†), where errors have been propagated *via* the covariance matrix obtained from fitting to the experimental data, and the MA extrapolation to the lowest temperature (red curve). Note that the predicted values of *k*_1_(*T*) increase markedly at low *T*, but never approach the values from CCT.

As a check of whether the parameterisation for *k*_1_(*T*) gives values that are realistic at the very lowest temperatures, classical capture theory (CCT) calculations have been carried out for the CN + CH_2_O reaction to calculate the rate coefficient at the collision limit *k*_coll_(*T*), using the molecular parameters given in Table S11 (ESI†). As shown in Fig. S9 (ESI†) for the CN and CH_2_O collision pair, *k*_coll_(*T*) is dominated by large dipole–dipole interactions below 600 K. As shown in Fig. 8, *k*_coll_ at ∼3 K is equal to 1.3 × 10^−9^ cm^3^ molecule^−1^ s^−1^, and from the MESMER simulations *k*_1_(*T*) is still calculated to be a factor of 10 or so less than *k*_coll_(*T*), and does not get close nor indeed exceed *k*_coll_(*T*) for all temperatures relevant to conditions used in astrochemical models.

## 4. Astrochemical modelling

Three different astrochemical environments were considered in order to explore the impact of the newly measured and evaluated rate coefficients (*k*_1_(*T*)) for the reaction of CN with CH_2_O over a wide range of temperatures. These were a dark cloud model for a total density of 2 × 10^4^ cm^−3^, at *T* = 5, 10 and 30 K, hot cores/corino models at higher densities and temperatures, and C-rich and O-rich AGB outflow models, further details of which are given in the following sections. The network for the model utilised the UMIST Rate12 kinetic database^77^ with updates to rate coefficients to include recent measurements of SiH with O_2_^79^ and CH with CH_2_O.^14^ Further details are given in Section 3.4, together with the parameterisation used for *k*_1_(*T*).

### 4.1 Dark cloud models

We calculated time-dependent chemical kinetic models using low-metal abundances appropriate for cold, dark interstellar clouds with a number density of *n*(H_2_) = 10^4^ cm^−3^ and temperatures of 5, 10 and 30 K, comparing results using the new Laval + Lit2 MA parameterisation to those obtained with the rate coefficient of the CN + CH_2_O reaction in the UMIST Rate12 (UDFA, https://udfa.ajmarkwick.net^77^) database. The latter adopts an energy barrier of 826 K (6.9 kJ mol^−1^) over the range 297–2500 K and, in the lack of low-temperature information, the rate coefficient extrapolates to zero at low temperatures. We find that the inclusion of reaction (1), even at its faster low-temperature fit, makes no difference to the abundances of CN, CH_2_O, HCO, HCN and HNC (see Fig. S11, ESI†). This arises because each of these common interstellar molecules are formed through a large variety of reactions, many of which involve highly abundant atoms and radicals such as O, N and CH_2_, as well as ion-neutral routes.

The inclusion of the title reaction does, however, impact on the abundance of HCOCN which our *ab initio* calculations rule out as a product of the CN + CH_2_O reaction. Previous modeling^13^ that included this product channel with a rate coefficient of up to 2 × 10^−10^ cm^3^ molecule^−1^ s^−1^ had shown that the calculated HCOCN fractional abundance was similar to that observed, 3.5 × 10^−11^, in the cold dark cloud TMC-1, (*n*(H_2_) = 10^4^ cm^−3^, *T* = 10 K).^80^ Our results indicate that, since CH_2_O is an abundant reactant, the total production rate of HCOCN is likely to be an order of magnitude less than the observational requirement.^80^

### 4.2 Hot core/corino models

We also investigated higher density, higher temperature models appropriate for hot core/corino sources. In particular, we modeled two of the hot sources in the Orion Molecular Cloud, the Orion Hot Core (*n*(H_2_) = 5 × 10^7^ cm^−3^, *T* = 225 K) and the Plateau (*n*(H_2_) = 10^6^ cm^−3^, *T* = 125 K). In these sources, initial abundances are molecular and reflect those in dust grain ice mantles which evaporate in the hot gas surrounding a massive young star. Our initial fractional abundances are taken from Doddipatla *et al.*^81^ Although these values are specific to these objects, our results are likely appropriate to other hot core/corino sources. Fig. S12 (ESI†) shows the abundances of HCO and HCN calculated for the Orion Hot Core, those for the Plateau source show a similar behaviour. Again, we find no discernible difference between our newly calculated abundances and those using the UDfA (UMIST Rate12^77^) rate coefficient. The major reason for this is that neither CN nor CH_2_O are abundant species in these evaporating interstellar ice mantles nor are they produced in significant fractions in the hot gas chemistry. We note in passing that the difficulty mentioned above, in reproducing the observed HCOCN abundance in hot sources, is likely to persist until kinetic data for any new significant HCOCN production reactions are measured or calculated.

### 4.3 Asymptotic giant star (AGB) outflows

The AGB outflow model is based on the publicly available UMIST CSE model (McElroy *et al.*^77^). Following Van de Sande *et al.*,^82^ the temperature of the outflows is parameterised as 
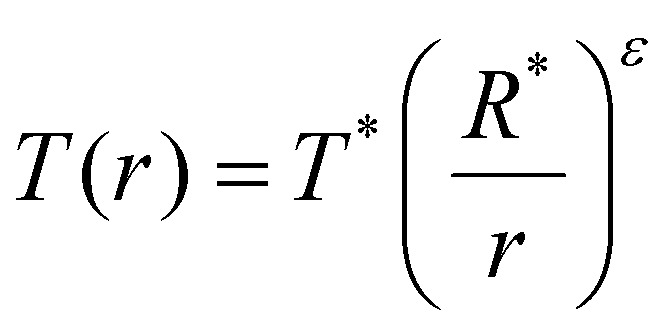
 where T* and R* are the stellar temperature and radius, respectively, and *ε* is the power law exponent. We assumed a value of R* = 5 × 10^13^ cm, T* = 2300 K and *ε* = 0.7. The outflow is assumed to be spherically symmetric with an expansion velocity of 15 km s^−1^, while varying the mass-loss rate between 10^−5^, 10^−6^, and 10^−7^ M_sun_ per year. Similar to West *et al.*,^14^ we considered both an O-rich and C-rich outflow, with the abundance of parent species taken from Agúndez *et al.*^83^

We find that changing the parameterisation of the reaction rate to the new measured and calculated rates does not affect the chemistry of both C-rich and O-rich outflows. The abundances and column densities of all species involved (CH_2_O, CH, HCO, HCN and HNC) are not changed. Fig. S13 and S14 (ESI†) show the abundances of HCO and HCN calculated for the high mass-loss rate O-rich and C-rich outflow, respectively. This behaviour is also seen in the lower mass-loss rate outflows.

## 5. Conclusions

Low-temperature rate coefficients, *k*_1_(*T*), for the reaction CN + CH_2_O have been measured for the first time below room temperature in a pulsed Laval apparatus using the PLP–LIF technique. A negative temperature dependence of *k*_1_(*T*) was observed below ∼100 K, in contrast to the positive temperature dependence of *k*_1_(*T*) reported previously in experiments performed above 300 K. Both *ab initio* calculations of the potential energy surface (PES) for the reaction and MESMER rate-theory simulations making use of this PES were performed in order to explore the overall mechanism that is consistent with the observed *k*_1_(*T*). Two low energy pathways were found on the PES through a VDW complex (binding energy of 13.3 kJ mol^−1^) with either a small positive barrier (3.97 kJ mol^−1^) to form HNC + HCO, or a submerged barrier (−0.62 kJ mol^−1^) to form HCN + HCO. This calculated PES can explain the negative temperature dependence of the rate coefficients at low temperatures but not the previously measured literature rate coefficients at room temperature and above. For the formation of formyl cyanide, HCOCN, a large activation barrier of 32.9 kJ mol^−1^ was calculated. Increasing both the energy of the transition state for the formation of HCN + HCO products from −0.62 to +4.0 kJ mol^−1^ (which is within the accuracy of the theory used) and also its imaginary frequency to 806 cm^−1^ in the MESMER simulations, enabled the temperature dependence of *k*_1_(T) across the entire experimental data to be reproduced very well. The energy and imaginary frequency of the other transition state for formation of HNC + HCO products were changed by the same amounts so their relative values were the same, ensuring that only the HCN + HCO remains the important one for this reaction. The postulated mechanism is similar to previous reactions of OH with volatile organic compounds where the formation of a weakly bound van der Waals complex, whose lifetime is extended at low temperature, is then followed by quantum mechanical tunneling through a small activation barrier to products.^3^

Using the MESMER package the product branching ratio was calculated and the formation of HNC was not found to be important as a reaction channel. MESMER was then used to simulate the rate coefficients *k*_1_(*T*) from 4–1000 K, and this dataset was used to recommend a parameterization from best-fit modified Arrhenius expressions for *k*_1_(*T*) for use in astrochemical modelling. Even at 4 K the simulated values of *k*_1_(*T*) were a factor of 10 less than the collision limit. This parameterization of *k*_1_(*T*) was used as input to astrochemical models of dark clouds, hot core/corinos, and Asymptotic Giant Star (AGB) stellar outflow environments using the UMIST Rate12 (UDfa) network of reactions. For a range of temperatures, the models yielded no significant changes in the abundances of HCN, HNC, and HCO upon inclusion of their formation using the rate coefficients reported here. It was found that the new rate coefficients for the reaction CN + CH_2_O do not have a significant effect on the modeled abundances of reagents and products of this reaction due to several other competing reactions with large rate coefficients. However, we note that the uncertainty at the CCSD(T)/aug-cc-pVTZ level of theory, (∼3.0–4.5 kJ mol^−1^) is similar to the difference in energy (4.59 kJ mol^−1^) calculated for the transition states forming HCN+HCO and HNC+HCO products. Hence, although HCN+HCO are the most likely products, it is possible that the HNC channel may also be the dominant one, as within the calculation errors the HNC+HCO transition state might be the lower one. In order to examine the impact of the scenario in which HNC is the dominant product of the reaction, the rate coefficient *k*_1_ was maintained at the same value, but it was assumed that HNC+HCO formed 100% of the products. For a 10 K rate coefficient and 100% formation to HNC, the title reaction provides less than 1% of the overall HNC production rate for cold environments such as molecular clouds. For the Orion Hot Core case at higher temperatures, the HNC+HCO reaction provides around 1% of the total HNC production rate. So, assuming 100% HNC production rather than very little production from the CN+CH_2_O reaction made a negligible difference to the modelled HNC abundance.

The main astrochemical implication of this paper is that the title reaction cannot contribute to the formation of formyl cyanide, HCOCN, in interstellar clouds, as has been suggested previously and currently implemented in astrochemical models.

## Author contributions

DEH and LD conceived the experiment, NAW and ER performed the experiments, LHDL and MAB performed the theoretical calculations, TJM and MVdS performed the astrochemical calculations, NAW, DEH and JHL wrote the manuscript with contributions from all authors.

## Conflicts of interest

There is no conflict of interest to declare.

## Supplementary Material

CP-025-D2CP05043A-s001
