# Supplementary material for: Experimental and theoretical study of the low-temperature kinetics of the reaction of CN with CH2O and implications for interstellar environments
Source: Phys Chem Chem Phys. 2023 Feb 24;25(11):7719–33. doi: 10.1039/d2cp05043a (PMC10015628; doi:10.1039/d2cp05043a)
Supplement: CP-025-D2CP05043A-s001 [file CP-025-D2CP05043A-s001.pdf]

Electronic Supplementary Material (ESI) for Physical Chemistry Chemical Physics.
This journal is © the Owner Societies 2023

Electronic Supplementary Information

Experimental and theoretical study of the low-temperature kinetics of the reaction of CN with CH_2O and implications for interstellar environments

Niclas A. West [a,1], Lok Hin Desmond Li [a], Tom J. Millar [b], Marie Van de Sande [c], Edward Rutter [a], Mark A. Blitz [a], Julia H. Lehman [a,2], Leen Decin [d], and Dwayne E. Heard [a*]

[a] *School of Chemistry, University of Leeds, Leeds, LS2 9JT, UK*

[b] *Astrophysics Research Centre, School of Mathematics and Physics, Queen's University Belfast, University Road, Belfast BT7 1NN, UK*

[c] *School of Physics and Astronomy, University of Leeds, Leeds, LS2 9JT, UK*

[d] *Instituut voor Sterrenkunde, KU Leuven, Celestijnenlaan 200D, 3001 Leuven, Belgium*

[1] *Current Address: Andor Technology Ltd., Belfast, BT12 7AL, UK*

[2] *Current Address: Current address: School of Chemistry, University of Birmingham, Edgbaston, B15 2TT, UK*

** Corresponding Author. Email: d.e.heard@leeds.ac.uk*

S1. Verification of the concentration of formaldehyde using UV absorption spectroscopy

After kinetics data were collected for each [CH_2O] generated by the gas mixing manifold, UV absorption spectra of final gas mixtures were measured in order to verify the CH_2O mixing ratio in the total flow going to the pulsed Laval nozzle. Further details of the spectrometer and absorption cell used can be found in West *et al.*, 2019.[1] Representative UV absorption spectra of CH_2O are shown in Fig. S1.

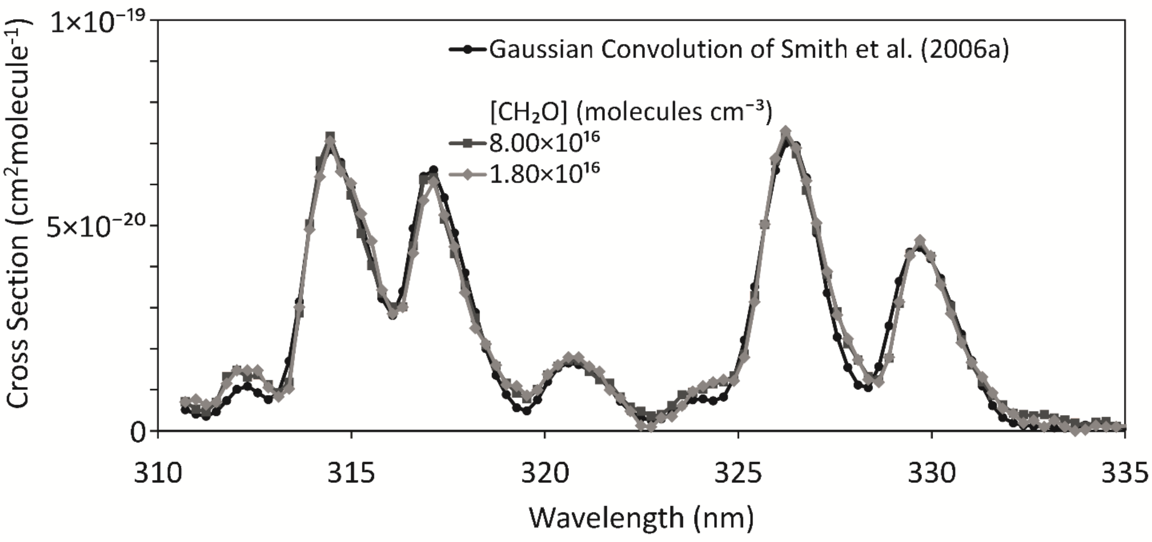

Fig. S1. Absorption spectra derived from two concentrations of CH_2O fitted to the convolution of a Gaussian function of 0.75 nm FWHM with a 0.0035 nm high-resolution spectrum from Smith *et al*.[2]

In order to compare each UV absorption spectrum collected in this study to the high-resolution literature spectrum,[2] the high-resolution spectrum was first convolved with a Gaussian function (FWHM = 0.75 nm) in order to match the resolution of the spectrometer utilized in this study. Then, a linear interpolation of the convolved spectrum was performed in order to match the wavelength grid of the spectra collected in this study. Next, each absorption spectrum was converted from absorbance values versus wavelength to absorption cross-section versus wavelength, using an initial estimate of the [CH_2O] in the absorbance cell and the known path length of the flow cell. Then, in order to obtain the true [CH_2O] in the absorption cell, a least-square analysis between the modified literature spectrum and the modified data spectrum was performed. Finally, the fraction of CH_2O in the Laval flow was determined by the fraction of CH_2O in the absorption cell based on the fitted [CH_2O] and the total pressure in the absorption cell.

S2. Laval flow characterization

The calculated flow temperature derived from Pitot tube impact pressure measurements versus the axial distance from the nozzle exit (d_{axial}) along the uniform supersonic flow is shown in Fig. S2.

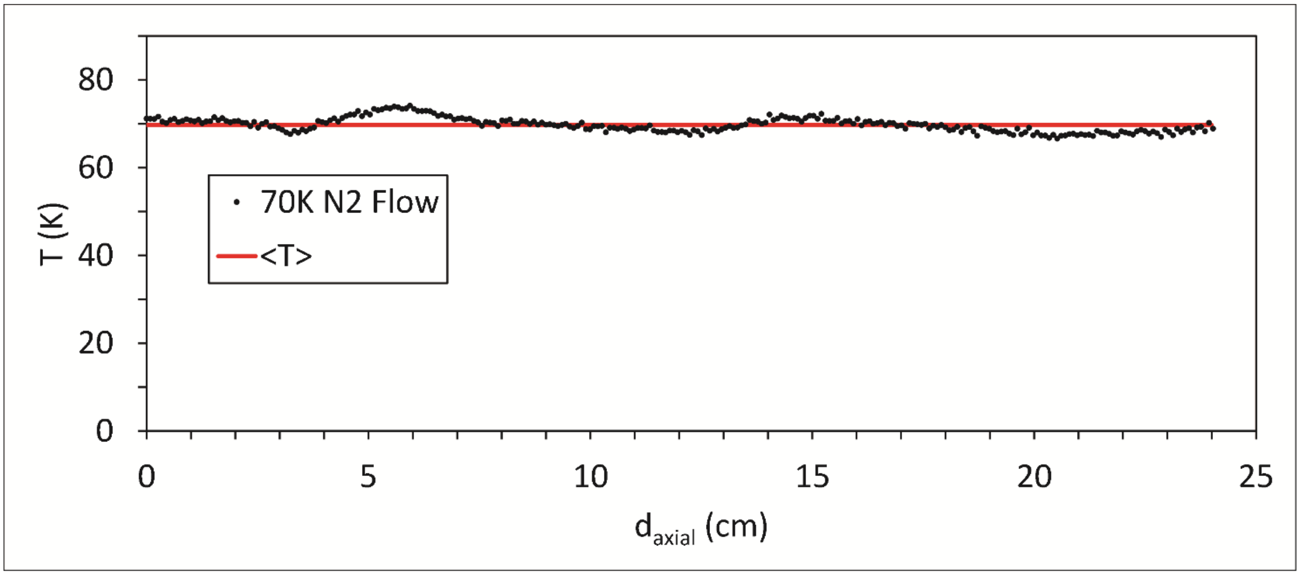

Fig. S2. Temperature values derived from Pitot tube impact pressure measurements in a N_2 bath-gas flow (black filled circles) and the average value of the measurements (70 K, red line, standard deviation of the measured temperature points is 2 K). d_{axial} represents the distance along the flow from the Laval nozzle exit.

S3. Second-order plots of the pseudo-first-order rate coefficient, k_{obs}, versus CH_2O concentration

Second-order plots of the pseudo-first-order rate coefficient, k_{obs}, versus CH_2O concentration for the reaction CN + CH_2O are shown in Fig. S3, but without subtraction of the intercepts at [CH_2O]=0 as in Fig. 3 of the main text. Intercepts of the second-order plots can be accounted for by the loss of the nascent radical being measured (CN) due to diffusion out of the volume traced out by the pump laser, as well as, to a small extent, the reaction of the nascent radicals with some of the species present in the flow. The largest reactive contributions to the intercept were likely due to reaction with the precursor, ICN, utilized to generate the radical, whose concentration was kept low. Second-order plots for CN + CH_2O, examples of which are shown in Fig. S3, did not curve over (show a decrease in the slope ($\bar{k}_{obs}$/[CH_2O])) at the CH_2O concentrations used.

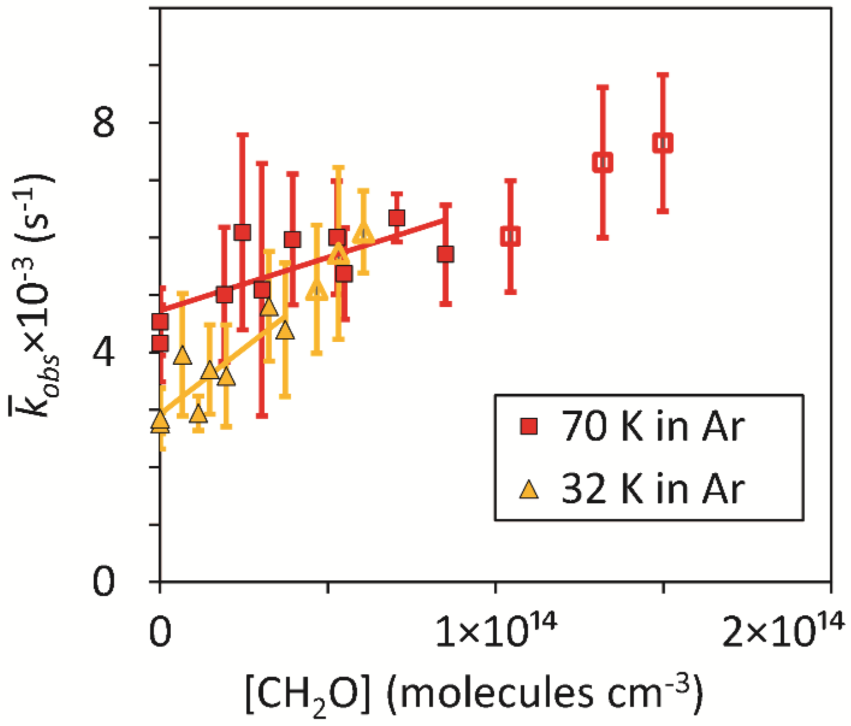

Fig. S3. Average pseudo-first-order rate coefficients for the loss of CN, $\bar{k}_{obs}$, versus the concentration of formaldehyde at two temperatures along with linear fits to the data for [CH_2O] < 1×10^{14} molecule cm^{-3} at each temperature. Error bars represent one standard deviation of fits of k_{obs} derived from at least 5 separate CN temporal traces.

S4. *Ab initio* quantum calculations using Gaussian

Tables S1 through S9 present the supplementary information on results from *ab initio* calculations, specifically the geometries and energies of stationary points along the CN + CH_2O reaction coordinate. Fig. S4 shows illustrations of the optimized geometries of the various stationary points, labelled as in the main text, particularly Fig. 5.

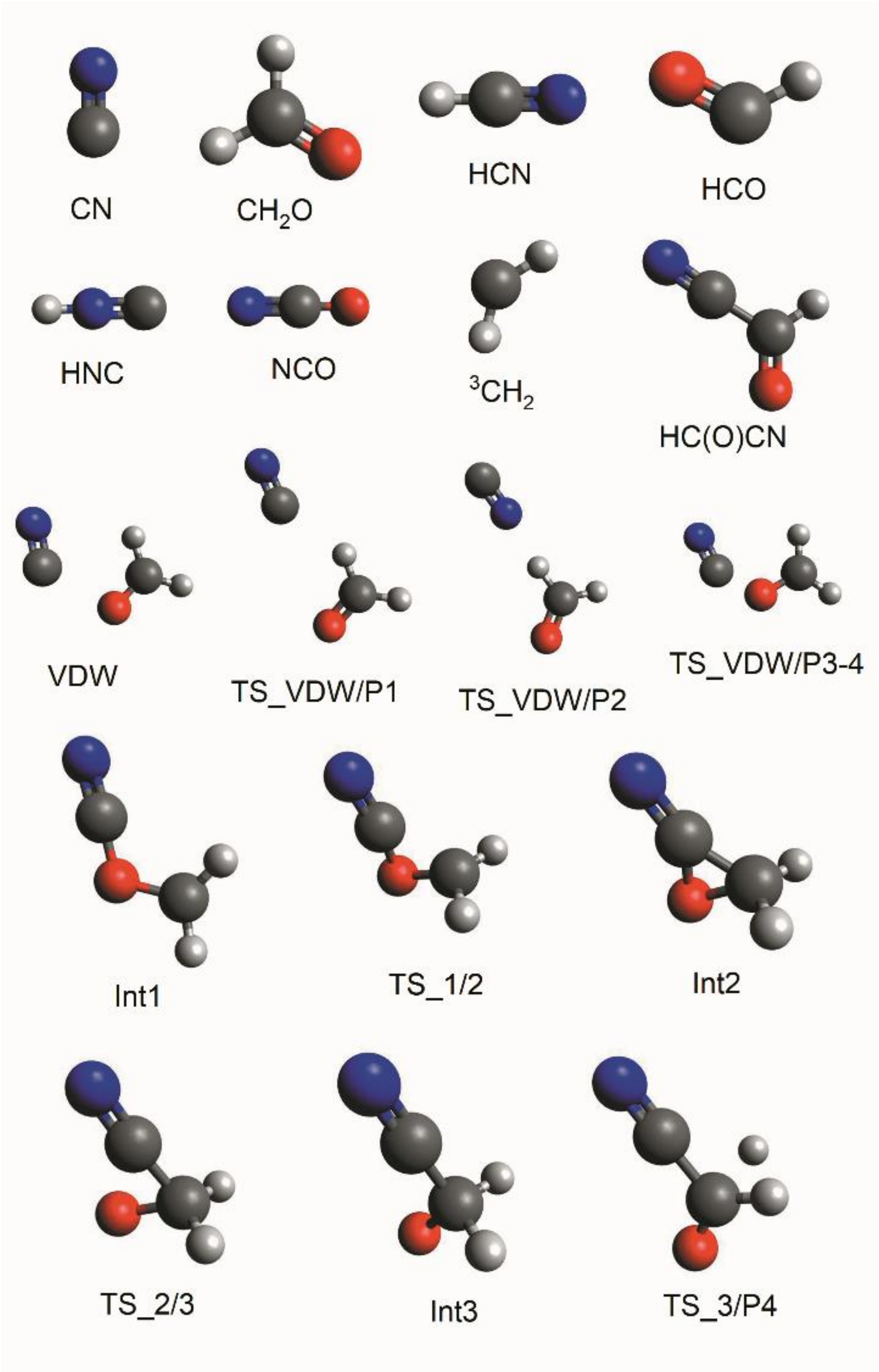

Fig. S4. Optimized geometries of reactants, products, intermediates, and transition states obtained at the M06-2X/aug-cc-pVTZ level of theory. The labels correspond to those shown on the PES in Fig. 5 of the main text.

Table S1. Energies (Hartree) of the stationary points indicated in Fig. 5 of the main text.

Molecule	BHandHLYP/aug-cc-pVDZ	CCSD(T)/aug-cc-pVTZ//BHandHLYP/aug-cc-pVDZ	M06-2X/aug-cc-pVTZ	CCSD(T)/aug-cc-pVTZ//M06-2X/aug-cc-pVTZ
CN	–92.6664471287	–92.570361289	–92.7109177045	–92.569940822
CH_2O	–114.454045365	–114.34248213	–114.498972536	–114.34256827
HCO	–113.808282507	–113.69205366	–113.849422185	–113.69211894
HCN	–93.3790556343	–93.280586134	–93.4240033474	–93.280464138
HNC	–93.3594694496	–93.257196852	–93.4034421705	–93.257105778
NCO	–167.937803563	–167.76167372	–168.001757582	–167.76182601
$^3CH_2$	–39.1344871171	–39.080082343	–39.1458950805	–39.080080114
HC(O)CN	–206.647867188	–206.43312185	–206.737485619	–206.43315497
H	–0.49807845526	–0.4998211760	–0.49820646135	–0.4998211760
VDW	–207.132179226	–206.92006960	–207.221791874	–206.92007837
TS_VDW/P1	–207.120946339	–206.91346038	–207.210896230	–206.91241474
TS_VDW/P2	–207.116552769	–206.90715307	–207.206033093	–206.90988176
TS_VDW/P3-4	–207.114313148	–206.90158148	–207.206393549	–206.90180253
Int1	–207.182819014	–206.96199619	–207.271625866	–206.96219122
TS_1/2	–207.149948879	–206.93246933	–207.241603107	–206.93246368
Int2	–207.171060223	–206.94904180	–207.258174359	–206.94900109
TS_2/3	–207.159894959	–206.94051055	–207.246790812	–206.94037893
Int3	–207.202787376	–206.97558554	–207.281454928	–206.97550823
TS_3/P4	–207.140727011	–206.92519810	–207.228517443	–206.92466699

Table S2. Zero-point energies (ZPVE) (Hartree) of the stationary points indicated in Fig. 5 of the main text.

Molecule	Unscaled BHandHLYP/aug-cc-pVDZ	Scaled BHandHLYP/aug-cc-pVDZ (scaling factor: 0.9589) [3]	Unscaled M06-2X/aug-cc-pVTZ	Scaled M06-2X/aug-cc-pVTZ (scaling factor: 0.956) [4]
CN	0.005120	0.004910	0.005108	0.004883
CH_2O	0.027595	0.026461	0.027038	0.025848
HCO	0.013500	0.012945	0.013274	0.012690
HCN	0.016876	0.016182	0.016612	0.015881
HNC	0.016059	0.015399	0.015985	0.015282
NCO	0.010383	0.009956	0.010285	0.009832
$^3CH_2$	0.017655	0.016929	0.017475	0.016706
HC(O)CN	0.027624	0.026489	0.027002	0.025814
H	0	0	0	0
VDW	0.035518	0.034058	0.034778	0.033248
TS_VDW/P1	0.032986	0.031630	0.031802	0.030403
TS_VDW/P2	0.030044	0.028809	0.030978	0.029615
TS_VDW/P3-4	0.034419	0.033004	0.034061	0.032562
Int1	0.037169	0.035641	0.036643	0.035031
TS_1/2	0.036018	0.034538	0.035395	0.033838

Int2	0.038832	0.037236	0.037962	0.036292
TS_2/3	0.036990	0.035470	0.036382	0.034781
Int3	0.037752	0.036200	0.036944	0.035318
TS_3/P4	0.028990	0.027799	0.028548	0.027292

Table S3. Relative energies of the stationary points indicated in Fig. 5 of the main text.

Molecule	Energy (CCSD(T)/aug-cc-pVTZ//M06-2X/aug-cc-pVTZ) + ZPVE (Scaled M06-2X/aug-cc-pVTZ frequencies) (Hartree)	Relative energy (Hartree)	Relative energy (kJ/mol)
CN	–92.565057574		
CH_2O	–114.316719942		
CN + CH_2O	–206.881777516	0	0
HCO	–113.679428996		
HCN	–93.264583066		
P1: HCO + HCN	–206.944012062	–0.062234546	–163.4
HNC	–93.241824118		
P2: HCO + HNC	–206.921253114	–0.039475598	–103.6
NCO	–167.75199355		
$^3CH_2$	–39.063374014		
P3: NCO + $^3CH_2$	–206.815367564	0.066409952	174.4
HC(O)CN	–206.407341058		
H	–0.4998211760		
P4: HC(O)CN + H	–206.907162234	–0.025384718	–66.7
VDW	–206.886830602	–0.005053086	–13.3
TS_VDW/P1	–206.882012028	–0.000234512	–0.62
TS_VDW/P2	–206.880266792	0.001510724	3.97
TS_VDW/P3-4	–206.869240214	0.012537302	32.9
Int1	–206.927160512	–0.045382996	–119.2
TS_1/2	–206.898626060	–0.016848544	–44.2
Int2	–206.912709418	–0.030931902	–81.2
TS_2/3	–206.905597738	–0.023820222	–62.5
Int3	–206.940189766	–0.058412250	–153.4
TS_3/P4	–206.897375102	–0.015597586	–41.0

Table S4. Optimized (BHandHLYP/aug-cc-pVDZ) Cartesian coordinates (in Å) of the stationary points.

Molecule	Atom	x	y	z
CN	C	0.0000000000	0.0000000000	0.0000070329
	N	0.0000000000	0.0000000000	1.1576529671
CH_2O	C	–0.0006269948	0.0000000000	–0.0004261838
	H	0.0000480566	0.0000000000	1.1029486987
	H	0.9868903289	0.0000000000	–0.4926113577

	O	–1.0163210369	0.0000000000	–0.6286262781
HCO	C	–1.1472608710	0.5108400621	–0.0693120621
	H	–2.1447035514	0.1497120150	0.2918159850
	O	–0.1159875776	0.1253359229	0.3161920771
HCN	C	–0.5012343391	0.2744630000	0.0000000000
	H	–1.5682846496	0.2744630000	0.0000000000
	N	0.6425389887	0.2744630000	0.0060188531
HNC	C	–0.6724460247	0.0859950800	2.1606269968
	H	–2.8299501604	0.0859950800	–0.0003623584
	N	–1.8358884249	0.0859950800	0.0025813953
NCO	C	–0.8286716441	0.3937950000	0.0935497776
	O	0.3372382922	0.3937950000	0.0900557182
	N	–2.0494519481	0.3937950000	0.0972090743
$^{3}CH_2$	C	–1.1518343493	0.8803618846	0.0000000000
	H	–0.5776428291	–0.0345635056	0.0000000000
	H	–2.2058255616	1.1167651410	0.0000000000
HC(O)CN	C	–4.4927412614	1.4312470592	1.6834996406
	H	–5.1162507065	2.3340601295	1.7150244670
	O	–3.3020663641	1.4587260846	1.6844629141
	C	–5.2500533317	0.1725471803	1.6395447126
	N	–5.8899343362	–0.7787584536	1.6063292656
VDW	C	0.1877328202	0.2711591310	–0.0330258805
	H	–0.2638490932	–0.6575597712	–0.4004401128
	H	0.9018087043	0.2168579409	0.7989691294
	O	–0.0830754105	1.3368653015	–0.5208162508
	C	–1.5175299875	0.9610130588	–2.1121827767
	N	–2.0220890333	0.0092783390	–2.5370281086
TS_VDW/P1	C	0.1359454235	0.2711243165	–0.2392824058
	H	1.2211917712	0.3136928104	–0.0177114384
	H	–0.3706552739	1.2435061946	–0.3585458806
	O	–0.4434274181	–0.7655423221	–0.3413919637
	C	3.2764185131	–0.9057404902	0.4234054596
	N	4.3024639841	–0.4095275092	0.6258532290
TS_VDW/P2	C	0.2661916136	–0.0236503220	0.6255598564
	H	1.3309111832	–0.1206752452	1.0254334191
	H	0.1859451594	–0.0084199370	–0.4785156740
	O	–0.6741770760	0.0541778703	1.3389681349
	C	3.5873140848	–0.3322721305	2.5342800458
	N	2.9672800350	–0.2691802355	1.5413972179
TS_VDW/P3-4	C	0.0920364602	0.2338898231	–0.0041991112
	H	–0.4428112964	–0.7137798210	0.0318507450
	H	1.0701180432	0.3407789529	0.4618604688
	O	–0.3772697775	1.2024634090	–0.6290982936
	C	–1.1261006683	0.8310588287	–2.1016021509
	N	–2.0129747613	0.2432038074	–2.5633356582
Int1	C	–0.0000008938	–0.0000061102	–0.0000014823
	H	–0.0000031530	–0.0000040487	1.0759509496
	H	0.8778810616	0.0000048253	–0.6268846190
	O	–1.1520814144	0.5622824272	–0.5224902562

	C	–1.2155804539	0.7114641955	–1.7979934490
	N	–1.3072451023	0.8637902144	–2.9331206285
TS_1/2	C	0.0931909504	0.1465526740	–0.3064537607
	H	0.0207105412	–0.8404824956	0.1250322672
	H	0.9592219679	0.7814679011	–0.1944497331
	O	–1.1432935063	0.8327859824	–0.4548828491
	C	–0.9337927174	0.4676094052	–1.6933915468
	N	–1.0194901257	0.2888840830	–2.8524941576
Int2	C	–2.1024967554	0.8015706954	0.0702475238
	H	–1.9179351684	–0.2654446290	0.0738140035
	H	–1.9248247908	1.3420588008	–0.8511123852
	O	–1.6552179011	1.4965763103	1.2748225196
	C	–2.9829519448	1.3708393455	1.0661845959
	N	–4.0687154396	1.6558544769	1.5696247425
TS_2/3	C	–2.0660532269	0.8323085785	0.1233983868
	H	–1.9313875963	–0.2470491519	0.0864227857
	H	–1.9382059875	1.3436290052	–0.8288204941
	O	–1.5294087403	1.4802575468	1.2455242471
	C	–3.1175694576	1.3213088099	0.9811071968
	N	–4.0695169913	1.6709992116	1.5959508777
Int3	C	2.1011728269	0.2673227771	–0.0263689090
	H	2.4306421008	–0.7781140666	–0.0879827475
	H	2.4306859917	0.7365453773	–0.9626032918
	O	2.7543879051	0.8656889591	1.0099136322
	C	0.6292312494	0.3168629311	0.0593544211
	N	–0.5154950739	0.3435340221	0.1054898949
TS_3/P4	C	2.0719329264	0.6454968366	0.0344230737
	H	2.4614827055	–1.1957132990	–0.1714942902
	H	2.4745770964	0.7109166164	–0.9835082782
	O	2.7181586587	0.9227998774	1.0152793237
	C	0.6202410070	0.4128591593	0.0998715841
	N	–0.5157683940	0.2554818094	0.1032315870

Table S5. Optimized (M06-2X/aug-cc-pVTZ) Cartesian coordinates (in Å) of the stationary points indicated in Fig. 5 of the main text.

Molecule	Atom	x	y	z
CN	C	0.0000000000	0.0000000000	0.0026490599
	N	0.0000000000	0.0000000000	1.1550109401
CH_2O	C	0.0001485802	0.0000000000	0.0000495740
	H	–0.0001405588	0.0000000000	1.1031921816
	H	0.9870338276	0.0000000000	–0.4928861636
	O	–1.0170514952	0.0000000000	–0.6290707129
HCO	C	–1.1478502975	0.5141164213	–0.0725884213
	H	–2.1413115962	0.1481177562	0.2934102438
	O	–0.1187901063	0.1236538225	0.3178741775
HCN	C	–0.5009704719	0.2744629999	0.0000000001
	H	–1.5672104034	0.2744630000	0.0000000000

	N	0.6412008754	0.2744630000	0.0000000000
HNC	C	0.0000000000	0.0000000000	2.1606269968
	H	0.0000000000	0.0000000000	0.0009053833
	N	0.0000000000	0.0000000000	0.9990676199
NCO	C	–0.8286470860	0.3937947400	0.0740993138
	O	0.3388251903	0.3937947400	0.0714754171
	N	–2.0512108442	0.3937947400	0.0768467291
$^{3}CH_2$	C	–1.1506440635	0.8820149934	0.0000000000
	H	–0.5835642543	–0.0316382648	0.0000000000
	H	–2.2010944222	1.1121867914	0.0000000000
HC(O)CN	C	0.0032967422	–0.0000002047	1.1906161951
	H	0.9209549067	0.0000004152	1.7928472351
	O	–0.0015665304	0.0000004720	–0.0017081873
	C	–1.2430500638	–0.0000019747	1.9813255879
	N	–2.1822720597	–0.0000033753	2.6374370909
VDW	C	–0.0802996529	0.1415542821	0.0886125371
	H	0.3496471560	–0.0961093270	1.0703091894
	H	0.5472902933	0.0377236402	–0.8054473223
	O	–1.2243327195	0.5096266809	0.0078247294
	C	–1.5449835579	0.8356580995	–2.1280613547
	N	–0.8443245190	0.7091606244	–3.0377607789
TS_VDW/P1	C	0.1716529423	0.2587231536	–0.2325318080
	H	1.2616271691	0.3753610457	–0.0227715791
	H	–0.3944269679	1.1975579239	–0.3527164016
	O	–0.3334755327	–0.8157694274	–0.3181451437
	C	3.1448813234	–0.6399711328	0.3875280146
	N	4.2716790658	–0.6283885630	0.6309639178
TS_VDW/P2	C	0.2113600000	–0.0187690000	0.6031100000
	H	1.2133530000	–0.1107640000	1.1232550000
	H	0.2482040000	–0.0123130000	–0.5040380000
	O	–0.8088380000	0.0649370000	1.2039010000
	C	3.9161060000	–0.3597260000	2.3440750000
	N	2.8832790000	–0.2633860000	1.8168200000
TS_VDW/P3-4	C	–0.0116283756	0.1813465161	0.0270360337
	H	0.0291975418	–0.2980508345	1.0030401411
	H	0.7985096458	0.0656845292	–0.6909816016
	O	–1.0343488706	0.8203321919	–0.2769992306
	C	–1.5011454612	0.6329863407	–1.8962339024
	N	–1.0775884803	0.7353152567	–2.9703854402
Int1	C	–0.0064939441	–0.0139702952	–0.0015974672
	H	0.0122374291	0.0112781460	1.0722481941
	H	0.8678708603	0.0047991567	–0.6309435748
	O	–1.1606985987	0.5580962510	–0.5156613165
	C	–1.2162074000	0.7120354299	–1.7963994727
	N	–1.2937106462	0.8653759968	–2.9321701796
TS_1/2	C	0.0879953265	0.1494321889	–0.3052931015
	H	0.0190707113	–0.8428251218	0.1105866138
	H	0.9574269136	0.7794121972	–0.2078289364
	O	–1.1499014895	0.8385652739	–0.4422518661

	C	–0.9371549787	0.4710474619	–1.6882849988
	N	–1.0008893731	0.2811855500	–2.8435674912
Int2	C	–2.0934364379	0.8028707424	0.0724275401
	H	–1.9211421562	–0.2651836430	0.0754841440
	H	–1.9280438389	1.3433442997	–0.8500462091
	O	–1.6509548511	1.4932901570	1.2691054179
	C	–2.9875027967	1.3727799976	1.0695708324
	N	–4.0710619193	1.6543524462	1.5670412748
TS_2/3	C	–2.0565893803	0.8355409283	0.1285849616
	H	–1.9384682443	–0.2456553068	0.0885760969
	H	–1.9428643134	1.3478379113	–0.8249170764
	O	–1.5124574751	1.4758666455	1.2429259904
	C	–3.1327740514	1.3178461229	0.9749756185
	N	–4.0689885354	1.6700166987	1.5934374090
Int3	C	2.1019519703	0.2689745078	–0.0233970520
	H	2.4311880099	–0.7772828818	–0.0910711717
	H	2.4311601750	0.7334860708	–0.9633416719
	O	2.7550285184	0.8665912714	1.0113692084
	C	0.6273189309	0.3161569553	0.0582102287
	N	–0.5160226045	0.3439140766	0.1060334586
TS_3/P4	C	2.0900040565	0.6431041278	0.0376043407
	H	2.3885960779	–1.1549710693	–0.1743549441
	H	2.4921201804	0.7060797797	–0.9829747076
	O	2.7284378186	0.9373352605	1.0186570996
	C	0.6320234888	0.3973763644	0.0971419418
	N	–0.5005576222	0.2229175369	0.1017302696

Table S6. Unscaled vibrational frequencies (cm^{-1}) of the stationary points in Table S4 (BHandHLYP/aug-cc-pVDZ). Four decimal place precision is used to display the values although the value of the imaginary frequency for some transition state species is changed by considerably more than this for the MESMER fitting exercise.

Molecule	Wavenumber (cm^{-1})
CN	2247.3106
CH_2O	1253.4937 1293.4062 1567.4704 1893.7491 3013.1585 3091.7026
HCO	1127.8469 2018.0359 2780.0316
HCN	781.9379 781.9379 2295.8436 3548.1368
HNC	469.8860 469.8873 2190.8722 3918.4467
NCO	541.0596 623.8794 1349.4921 2043.3909
$^3CH_2$	1091.4693 3204.6888 3453.4607
HC(O)CN	244.3149 313.7408 647.4999 964.6374 1045.5923 1444.7613 1882.3985 2457.8298 3124.8194
VDW	112.5553 114.5264 274.7901 299.5249 322.1122 1271.1946 1282.3253 1544.0397 1844.3247 2254.3442 3080.8598 3189.8143

TS_VDW/P1	–139.2544 27.2536 37.2742 55.8459 116.4252 1249.7897 1275.1838 1558.0157 1895.5680 2250.0120 2941.3277 3072.5828
TS_VDW/P2	–539.1909 38.0694 65.5620 151.7302 294.6677 1208.5307 1250.1347 1414.3783 1556.2042 1960.5812 2239.6025 3008.2362
TS_VDW/P3-4	–936.1640 98.5446 158.9751 449.6762 494.2486 1071.7107 1250.8951 1360.1802 1567.5274 2218.5304 3149.2681 3288.8040
Int1	216.8530 252.1633 502.3936 537.7094 646.8613 985.8456 1204.3661 1302.3194 1491.2028 2482.6848 3258.2562 3434.5003
TS_1/2	–855.2504 365.7461 432.8525 622.8138 844.4755 1013.2573 1135.0087 1162.7004 1495.0897 2102.5012 3235.7245 3399.7794
Int2	444.5194 445.2710 760.6977 942.9260 1019.6413 1120.4699 1133.0812 1223.1826 1529.6402 1876.0723 3217.8224 3332.1531
TS_2/3	–781.9591 377.3786 393.3708 882.3567 1040.3693 1147.6051 1169.5877 1282.5490 1541.5014 1989.0210 3161.6952 3251.2184
Int3	238.0977 347.7046 608.5778 650.3364 939.2090 1117.4834 1200.9026 1378.6666 1423.2928 2474.3670 3080.2164 3112.5181
TS_3/P4	–836.2741 242.6908 283.8152 407.6675 451.0981 644.5861 958.4904 1020.5629 1423.6047 1712.7181 2458.5362 3121.2336

Table S7. Unscaled vibrational frequencies (cm^{-1}) of the stationary points in Table S5 (M06-2X/aug-cc-pVTZ). Four decimal place precision is used to display the values although the value of the imaginary frequency for some transition state species is changed by considerably more than this for the MESMER fitting exercise.

Molecule	Wavenumber (cm^{-1})
CN	2241.9799
CH_2O	1216.3324 1275.0095 1540.7191 1869.4302 2948.5832 3018.3473
HCO	1103.4473 1993.0627 2729.8956
HCN	785.3481 785.3481 2254.5540 3466.4178
HNC	528.7255 528.7255 2147.7615 3811.6074
NCO	533.0140 614.6710 1326.9732 2039.9448
$^3CH_2$	1096.3069 3177.8218 3396.7268
HC(O)CN	235.3323 310.0780 630.6469 932.1926 1016.9040 1415.0844 1853.2170 2407.2828 3051.7761
VDW	116.7742 129.4838 262.3213 301.7164 306.4708 1229.0710 1264.4117 1513.7341 1821.9598 2212.0823 3004.5679 3103.2415
TS_VDW/P1	–214.6595 23.3018 35.5117 50.7396 91.0436 1196.6207 1227.9601 1509.2793 1876.0265 2226.6387 2725.6977 2996.8099

TS_VDW/P2	–222.6725 48.6415 67.4455 134.4869 266.0340 1209.1278 1212.2454 1479.9321 1841.4504 2080.0746 2325.2605 2932.8990
TS_VDW/P3-4	–850.0448 114.6174 180.3015 455.8172 522.2467 1064.1180 1230.1891 1379.3882 1546.5007 2177.9188 3075.0931 3204.8833
Int1	229.7909 247.1081 526.2839 557.0405 641.1308 975.9125 1189.5383 1281.1086 1469.3312 2432.9556 3187.0090 3347.4220
TS_1/2	–781.1002 372.5848 426.2327 606.2279 809.4990 992.8945 1131.9599 1142.4768 1465.7413 2100.9242 3166.7193 3321.3474
Int2	428.9031 437.4089 778.5354 899.1928 986.8137 1110.9110 1125.4648 1186.6388 1498.4004 1814.2092 3145.0780 3251.9897
TS_2/3	–661.6184 364.7564 387.8360 866.2833 1012.5754 1134.0929 1168.9878 1254.5642 1511.9142 2015.1175 3085.8493 3167.9682
Int3	232.3747 341.2933 598.7539 669.1942 913.7207 1093.4184 1182.8619 1344.0640 1391.4860 2424.4943 3000.6790 3024.2660
TS_3/P4	–927.9940 234.7680 284.0235 429.7689 469.1457 627.3944 922.0683 1015.6697 1393.0411 1721.4685 2403.7170 3029.8942

Table S8. Rotational constants (GHz) of the stationary points in Table S4 (BHandHLYP/aug-cc-pVDZ).

Molecule	Rotational constants (GHz)
CN	58.356032
CH_2O	284.94442 39.54571 34.72626
HCO	735.99076 45.40460 42.76627
HCN	45.114810
HNC	45.854396
NCO	11.876083
$^3CH_2$	1696.16249 252.20695 219.55999
HC(O)CN	69.20026 5.05119 4.70756
VDW	30.74881 4.38635 3.83875
TS_VDW/P1	39.06318 2.08177 1.97645
TS_VDW/P2	39.86240 2.62094 2.45925
TS_VDW/P3-4	38.78166 5.05054 4.57598
Int1	52.55168 5.55680 5.03272
TS_1/2	29.12886 7.41526 6.17356
Int2	26.72865 8.25964 6.59524
TS_2/3	26.91551 7.68428 6.22771
Int3	41.55366 4.93283 4.53128
TS_3/P4	42.80559 4.87796 4.60759

**Table S9**. Rotational constants (GHz) of the stationary points in Table S5 (M06-2X/aug-cc-pVTZ).	
Molecule	Rotational constants (GHz)
CN	58.892432
CH_2O	284.75755 39.45537 34.65382
HCO	720.91067 45.43688 42.74292
HCN	45.232416
HNC	45.932654
NCO	11.842920
$^3CH_2$	1671.48960 255.53548 221.64989
HC(O)CN	68.47755 5.04092 4.69528
VDW	29.57540 4.52700 3.92605
TS_VDW/P1	44.25913 2.22853 2.12172
TS_VDW/P2	63.66131 2.27792 2.19922
TS_VDW/P3-4	36.12996 5.25240 4.68349
Int1	51.43216 5.57054 5.03485
TS_1/2	28.66960 7.46924 6.18979
Int2	26.99825 8.20895 6.57940
TS_2/3	26.95494 7.61005 6.18138
Int3	41.58770 4.92674 4.52590
TS_3/P4	43.54483 4.86961 4.59540

As discussed in the main text, a series of relaxed scans were performed in order to understand the approach of CN to different sides of CH_2O. As the carbon side of CN approaches the oxygen side of CH_2O, CN moves to spin towards the geometry of the van der Waals complex in a barrierless process (Fig. S5). There is a slight discontinuity in the scan shown here, mostly due to issues around the molecule moving between planar and non-planar configurations as the carbon-oxygen distance was scanned to produce this figure, but no barrier was found when this was investigated further. As the nitrogen end of CN approaches the carbon of CH_2O (Fig. S6), CN moves to orient itself along the C_2 axis, bisecting the HCH bond angle. When restricting the symmetry in a geometry optimization procedure, a weakly bound C_{2v} complex can be formed in this way, but it was not significantly stable when allowing the symmetry to relax. As shown in the main text (Fig. 6), when we allow the lowest energy structure found in Fig. S6 to further optimize, it is also able to form the van der Waals complex in a nearly barrierless process. The relaxed scan on the C-C separation in the adduct $H_2C(O)CN$ (Int3) was also performed (Fig. S7), which suggested that the direct addition from/dissociation to the starting reagents involves a very large barrier.

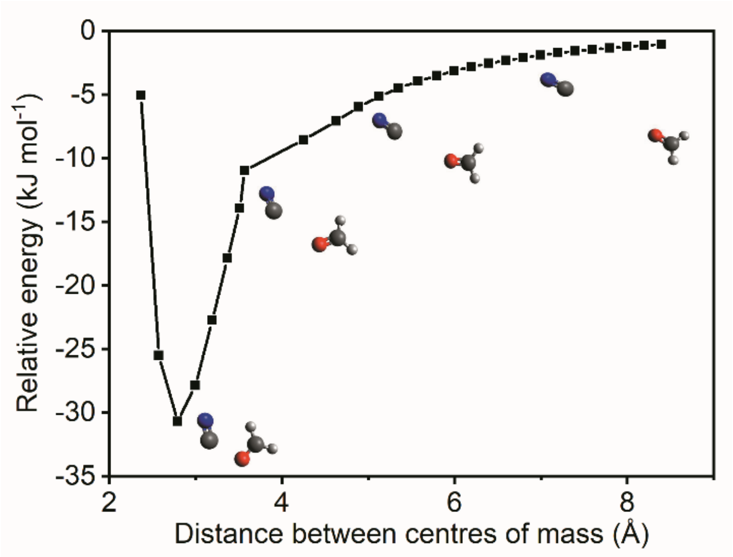

Fig. S5. Potential energy curve (BHandHLYP/aug-cc-pVDZ, uncorrected for ZPVE) for the approach of CN from the oxygen side of the CH_2O. The scan coordinate is the distance between the carbon atom of the CN moiety and the oxygen of CH_2O.

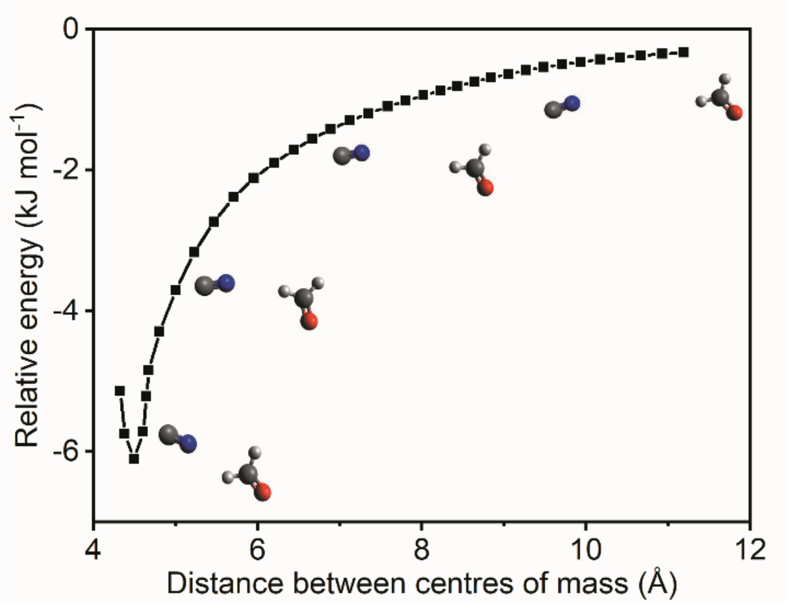

Fig. S6. Potential energy curve (BHandHLYP/aug-cc-pVDZ, uncorrected for ZPVE) for the approach of CN from the hydrogen side of the CH_2O. The scan coordinate is the distance between the carbon atom of the CN moiety and the hydrogen atom of CH_2O.

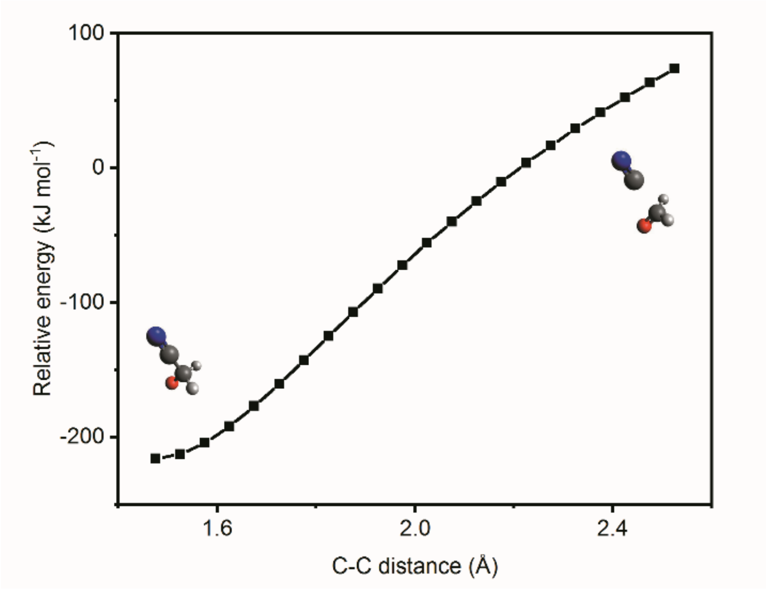

Fig. S7. Potential energy curve (BHandHLYP/aug-cc-pVDZ, uncorrected for ZPVE) for the direct dissociation of CN from the adduct $H_2C(O)CN$ (Int3). The scan coordinate is the distance between the carbon atom of the CN moiety and the carbon atom of CH_2O.

S5. MESMER calculations

Table S10 gives the recommended values of k_1 from 4 to 1000 K from the MESMER simulations of the *Laval* + *Lit 2* model. The fractional errors (2σ) were determined by propagating the errors from the fit to the data within MESMER. It was not possible to propagate errors below 7 K

Table S10. Tabulated values for k_1 (cm^3 molecule^{-1} s^{-1}) and associated 2σ error from the MESMER simulations using the *Laval* + *Lit 2* model. Also included are simulations using the *Laval* model. Three decimal places are used to display the values although the real precision is one decimal place.

T **(K)**	$\boldsymbol{k_1}$ **from** ***Laval + Lit 2***	**2σ error in** $\boldsymbol{k_1}$ **% / 100**	$\boldsymbol{k_1}$ **from** ***Laval***
3			1.012E-09
4	1.299E-10		6.339E-10
5	1.138E-10		4.534E-10
6	9.735E-11		3.662E-10
7	9.524E-11	0.520	2.852E-10
8	9.187E-11	0.497	2.321E-10

9	8.799E-11	0.476	1.949E-10
10	8.398E-11	0.456	1.676E-10
11	8.003E-11	0.437	1.467E-10
12	7.625E-11	0.420	1.303E-10
13	7.268E-11	0.403	1.171E-10
14	6.934E-11	0.388	1.063E-10
15	6.622E-11	0.373	9.718E-11
16	6.332E-11	0.359	8.950E-11
17	6.062E-11	0.345	8.291E-11
18	5.811E-11	0.333	7.720E-11
19	5.578E-11	0.321	7.221E-11
20	5.360E-11	0.309	6.781E-11
20	5.360E-11	0.309	6.781E-11
25	4.468E-11	0.261	5.187E-11
30	3.816E-11	0.223	4.192E-11
30	3.816E-11	0.223	4.192E-11
35	3.321E-11	0.195	3.512E-11
40	2.934E-11	0.174	3.020E-11
45	2.625E-11	0.149	2.647E-11
50	2.372E-11	0.140	2.355E-11
60	1.985E-11	0.138	1.927E-11
70	1.702E-11	0.195	1.630E-11
80	1.488E-11	0.140	1.411E-11
80	1.494E-11	0.140	
90	1.298E-11	0.143	
100	1.185E-11	0.145	
125	1.073E-11	0.164	
150	1.071E-11	0.138	
175	1.119E-11	0.116	
200	1.198E-11	0.100	
225	1.298E-11	0.088	
250	1.413E-11	0.079	
275	1.542E-11	0.072	
300	1.682E-11	0.071	
300	1.657E-11	0.071	
325	1.833E-11	0.067	
350	1.999E-11	0.065	
375	2.154E-11	0.064	
400	2.300E-11	0.064	
425	2.437E-11	0.065	
450	2.565E-11	0.067	
475	2.685E-11	0.070	
500	2.798E-11	0.074	
525	2.904E-11	0.078	
550	3.004E-11	0.082	
575	3.098E-11	0.087	
600	3.187E-11	0.092	
625	3.270E-11	0.096	
650	3.350E-11	0.101	

675	3.425E-11	0.105	
700	3.496E-11	0.110	
725	3.563E-11	0.114	
750	3.627E-11	0.119	
775	3.688E-11	0.123	
800	3.746E-11	0.127	
825	3.801E-11	0.131	
850	3.854E-11	0.135	
875	3.904E-11	0.139	
900	3.952E-11	0.142	
925	3.998E-11	0.146	
950	4.042E-11	0.149	
975	4.084E-11	0.152	
1000	4.124E-11	0.156	

The MESMER input file (.xml format) can be found at the end of this Supplementary Information, where the energy transfer parameters of the various bath gases are given.

Fig. S8(a) shows all of the experimental data for $k_1(T)$ together with the MESMER simulations using the *Laval + Lit 2* model.

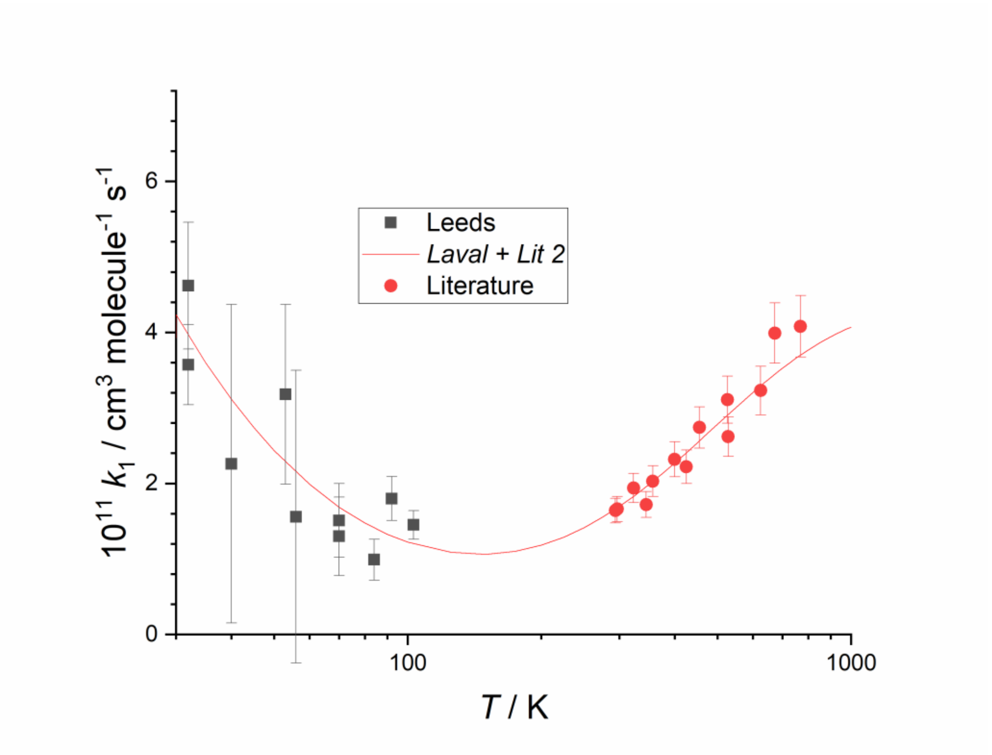

Fig. S8(a). All of the experimental data for $k_1(T)$, namely the low temperature data obtained using the Laval nozzle (black squares) and the literature data at higher temperatures (red circles) together with the MESMER simulations using the *Laval + Lit 2* model (red line).

As discussed in the main text, the primary products over the entire temperature range covered here are HCN + HCO. Fig. S8(b) shows the fractional yield of HCN and HNC products as a function of temperature. The HNC yield was calculated by maintaining the *ab initio* energy difference between the two transition states leading to HCN and HNC products (the transition state for HNC formation being ~4.59 kJ mol^{-1} higher), and the imaginary frequency for the transition-state leading to HNC was also increased by the same amount (to ~ 806 cm^{-1}) as in the *Laval + Lit 2* model for the transition state leading to HCN, as detailed in the main paper.

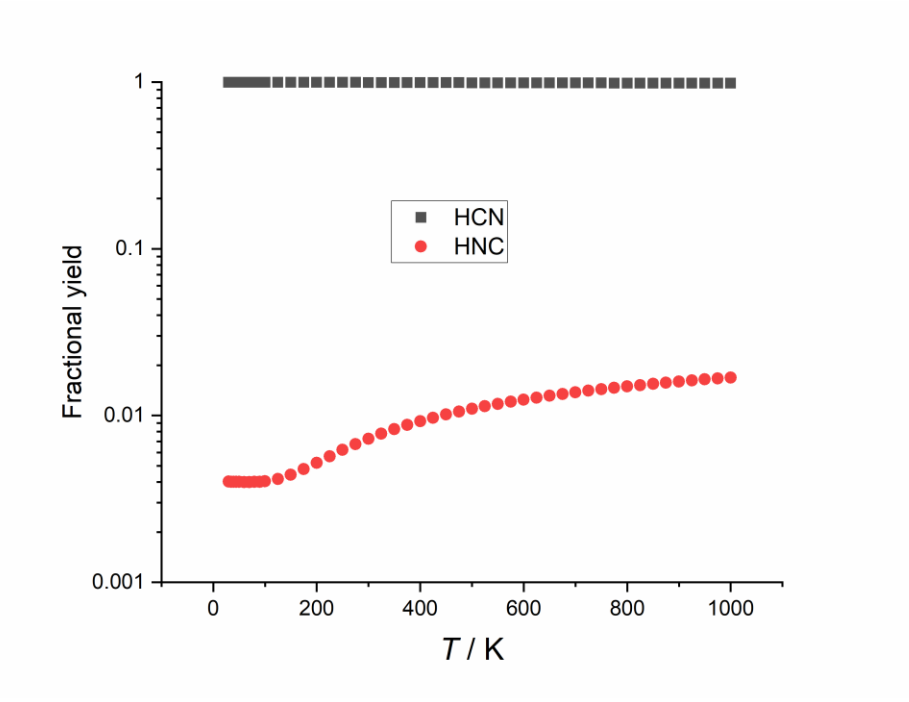

Fig. S8(b). Predicted branching ratios of the CN + CH_2O reaction as a function of temperature using the parameters obtained from the *Laval + Lit 2* model, and maintaining the ab initio calculated energy difference between the transition states forming HCN and HNC, with both transition states having an imaginary frequency of 806 cm^{-1}.

S6. Classical capture theory

One important feature that should ultimately limit the negative temperature dependence of reaction rate coefficients is the collision limit, which is when the rate of reaction is equal to the rate of collisions between reagent molecules. Second-order rate coefficients can be described as:

	$$k(T) = \sigma(T)\langle v(T)\rangle$$	(S1)

where $k(T)$ is a temperature-dependent rate coefficient (cm^3 $molecule^{-1}$ s^{-1}), $\sigma(T)$ is a temperature-dependent cross-section (cm^2 $molecule^{-1}$), and $<v(T)>$ is the temperature-dependent average velocity (cm s^{-1}). The simplest hard-sphere model of the collision limit would predict a $T^{1/2}$ dependence due entirely to the temperature dependence of the average velocity. If instead of modeling the reagent molecules as hard spheres, the orientation-averaged attractive intermolecular potential between the reagent molecules is considered, then the reagent molecules are deflected toward each other and collisions occur more often at lower temperatures when average molecular velocities are lower. This relatively simple method for approximating the collision limit, k_{coll}, is called classical capture theory (CCT). [5] The CCT derivation is given here to allow illustration of the contribution of the various components of long-range attractive forces at play, and is similar to that presented in West et al. 2017 [1]. When solving for k_{coll} with CCT using the most significant contributing forces to the long-range potential experienced between neutral-neutral molecular species, dipole-dipole ($D - D$), dipole-induced-dipole ($D - iD$), and London dispersion ($Disp$) forces, the form of the k_{coll} equation is:

	$$\begin{aligned} k_{coll}(T) &= \sigma_{coll}(T)\langle v(T)\rangle \\ &= \left[\pi\left(\frac{2C_6}{k_BT}\right)^{1/3}\Gamma\left(\frac{2}{3}\right)\right]\left[\left(\frac{8k_BT}{\pi\mu}\right)^{1/2}\right] \end{aligned}$$	(S1)

where $\Gamma(x)$ is the gamma function such that $\Gamma(2/3) = 1.353$, k_B is the Boltzmann constant, μ is the reduced mass and C_6 is the sum of coefficients describing forces between collision partners:

	$$C_6 = C_6^{D-D} + C_6^{D-iD} + C_6^{Disp}$$	(S2)

C_6^{D-D} can be described by:

	$$C_6^{D-D} = \frac{2}{3}\left(\frac{\mu_1^2\mu_2^2}{k_BT(4\pi\epsilon_0)^2}\right)$$	(S3)

where μ_1 and μ_2 are the dipole moments of reagents 1 and 2 and ϵ_0 is the permittivity of free space.[6] C_6^{D-iD} can be described by:

	$$C_6^{D-iD} = \frac{\mu_1^2\alpha_2 + \mu_2^2\alpha_1}{4\pi\epsilon_0}$$	(S4)

where α_1 and α_2 are the polarizabilities of reagents 1 and 2. C_6^{Disp} can be described by:

	$$C_6^{Disp} = \frac{3}{2}\alpha_1\alpha_2\left(\frac{I_1I_2}{I_1 + I_2}\right)$$	(S5)

where I_1 and I_2 are the ionization energies of reagents 1 and 2. When CCT is utilized to calculate k_{coll}, this can provide an approximate upper limit on the extrapolation of fits of the negative temperature dependence of reaction rate coefficients measured at low temperature. There are several other methods of calculating the collision limit more accurately[7] including: rotationally Adiabatic Capture (AC) theory,[8-10] Statistical

Adiabatic Capture Model (SACM),[11, 12] and long-range E,J-resolved microcanonical Variational Transition State Theory (μj-VTST).[13] To a good first order approximation, however, CCT can be used to estimate the collision limit in order to avoid extrapolation of negative temperature dependencies to values which are wrong by orders of magnitude.

In order to examine the relative effects of D-D, Disp, and D-iD interactions on the calculation of $k_{coll}(T)$ for the CN and CH_2O collision pair, a comparison of $k_{coll}(T)$ curves with the individual interactions only and the summation of the interactions for the CN and CH_2O collision pair are shown in Fig. S9.

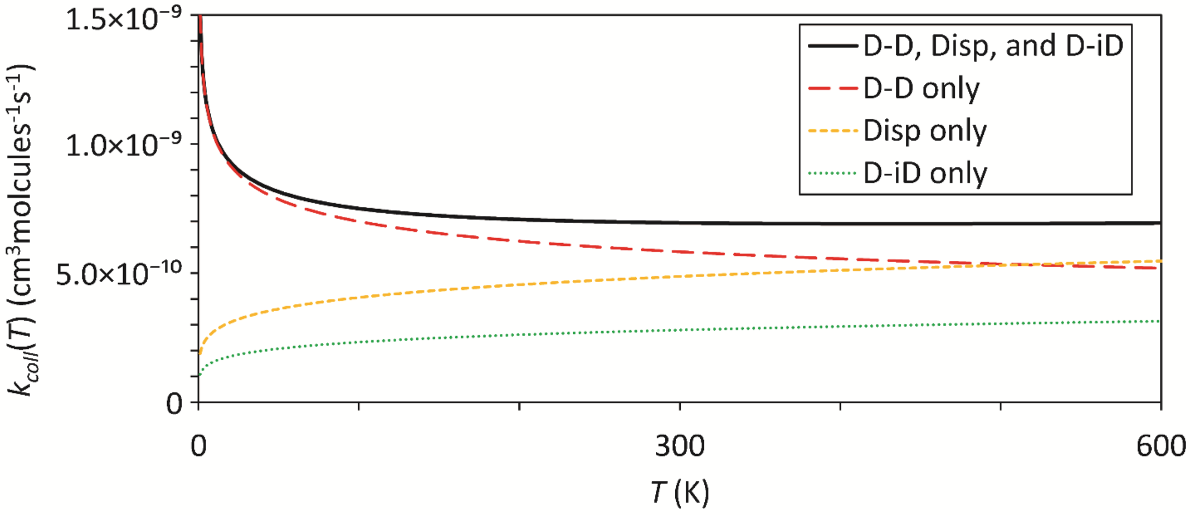

Fig. S9. The capture rate coefficient $k_{coll}(T)$ calculated using Equation (S1), together with the various relative contributions. In order to show the relative contribution of each intermolecular force to the total $k_{coll}(T)$, "$D-D$ only," "*Disp* only," and "$D-iD$ only" curves were calculated with $C_6 = C_6^{D-D}$, $C_6 = C_6^{Disp}$, and $C_6 = C_6^{D-iD}$ respectively, as given in equations SS3-SS5. See text for details.

In this work, the molecular constants utilized to calculate $k_{coll}(T)$ for CN + CH_2O are given in Table S11.

Table S11. Parameters utilized to calculate $k_{coll}(T)$ between CN and CH_2O.

Molecule	Dipole Moment		Polarizability	Ionization Energy	
	μ_n		α_n	I_n	
	(Debye)	(C cm)	(cm^3)	(eV)	(J)
CH_2O	2.33	7.77×10^{-28} [1]	2.77×10^{-24} [3]	10.8887	1.74×10^{-18} [5]
CN	1.45	4.83×10^{-28} [2]	2.70×10^{-24} [4] ^{a}	13.598	2.18×10^{-18} [6]

(a) Estimated from calculation values. (Since $D-D$ interactions dominate at ~ 10 K, small changes to this value negligibly affect $k_{coll}(T)$ calculations). References: [1];[14] [2];[15] [3];[16] [4];[17] [5]; [18] [6][17]

A comparison of $k_{coll}(T)$ with $k_1(T)$ values can be made by comparing curves in Fig. S9 and S10.

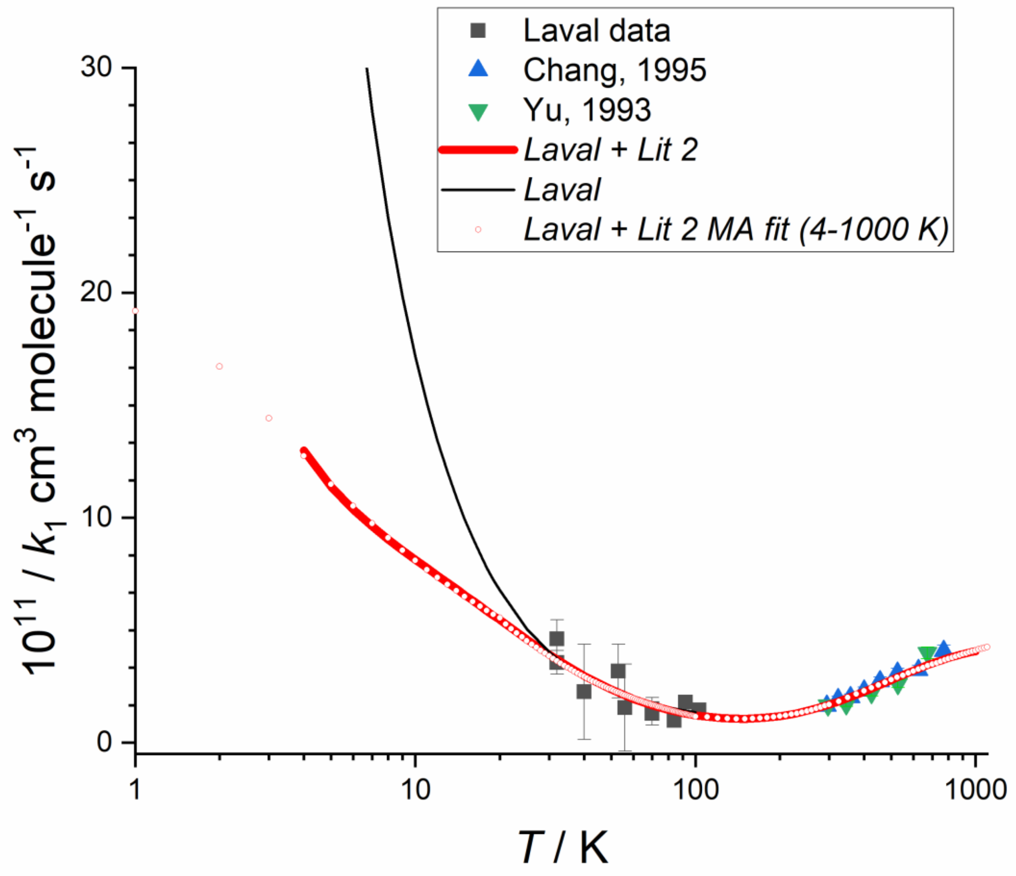

Fig. S10. The red circles represent the modified Arrhenius (MA) fits to the *Laval + Lit 2* model data over the four temperature ranges, see Table 3. Similarly, the black line is the fit to the *Laval* model data. The other data in the figure are the same as given in Fig 4.

S7. Astrochemical modeling

See main text Section 4 for a description of the astrochemical model and the three different environments considered. Fig. S11 – S14 below represent the results described in Section 4 of the main text for a dark cloud model (Section S8.1), a hot core/corino model (Section 8.2) and both an O-rich and C-rich AGB outflow model (Section S8.3).

S8.1 Dark cloud model

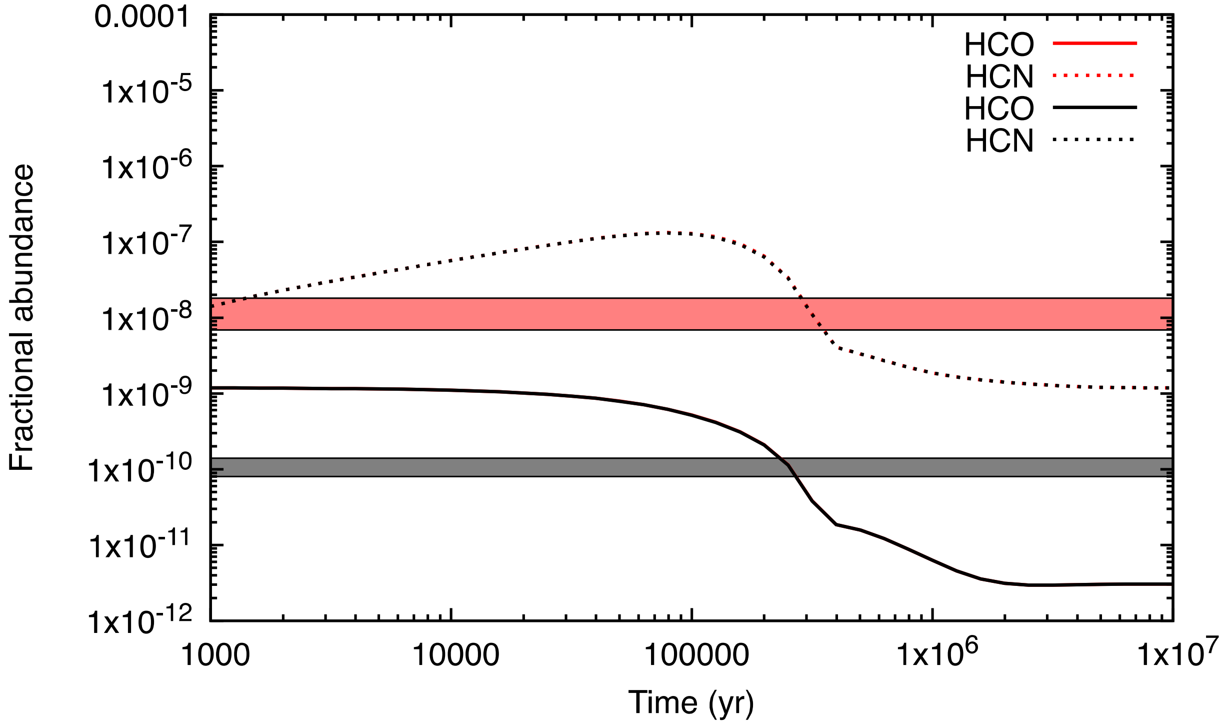

Fig. S11. Time evolution of the HCO and HCN abundances for a cold (10 K), dense ($n(H_2) = 10^4$ cm^{-3}) interstellar cloud. Red curves relate to the base model, black to the model in which we adopt the fast rate coefficient for the title reaction at low temperatures as discussed in Sect. 4.1. The black and red shaded areas represent the observed abundances of HCO and HCN in TMC-1, respectively (Cernicharo J et al A&A, 665, L21, 2021; Pratap P et al, ApJ, 486, 862, 1997). The overlap of red and black curves shows that our new rate coefficient does not change the abundances of HCO and HCN.

S8.2 Hot core/corino model

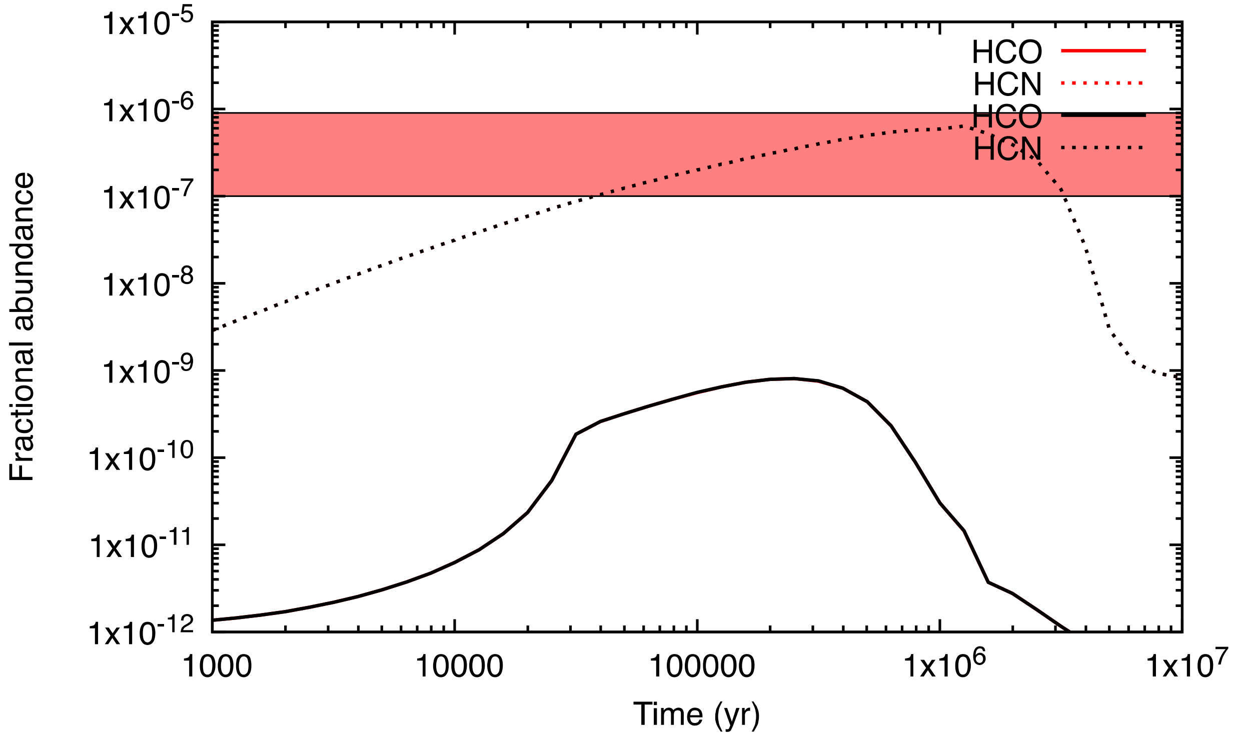

Fig. S12. Time evolution of the HCO and HCN abundances for conditions appropriate to the Orion Hot Core (225 K), dense ($n(H_2) = 5 \times 10^7$ cm^{-3}) interstellar cloud. Red curves relate to the base model, black to the model in which we adopt the fast rate coefficient for the title reaction at low temperatures as discussed in Sect. 4.1. The red shaded area represents the observed abundance of HCN in the Orion Hot Core (Blake G A et al, ApJ, 315, 621, 1987). HCO has not been detected in this source. The overlap of red and black curves shows that our new rate coefficient does not change the abundances of HCO and HCN.

S8.3 AGB outflow model

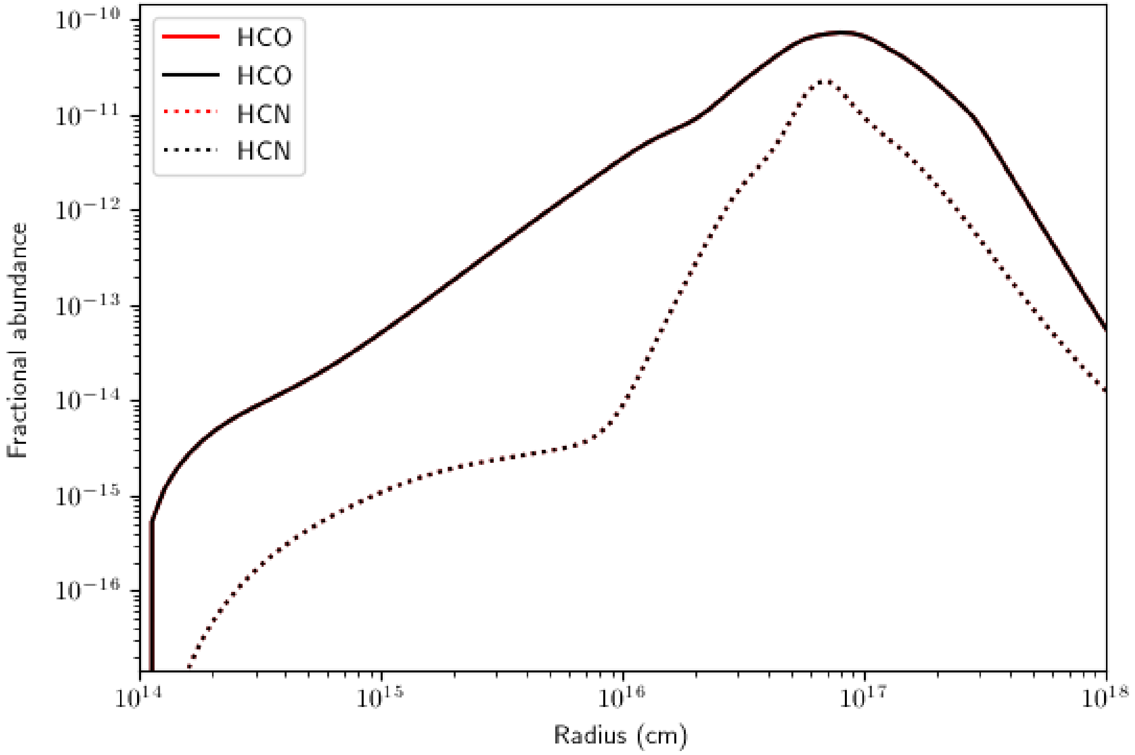

Fig. S13. HCO and HCN profiles throughout the O-rich AGB outflow with a mass-loss rate of 10^{-5} M_{sun}/yr. Red curves relate to the base model, black to the model in which we adopt the fast rate coefficient for the title reaction at low temperatures as discussed in Sect. 4.1. The overlap of red and black curves shows that our new rate coefficient does not change the abundances of HCO and HCN.

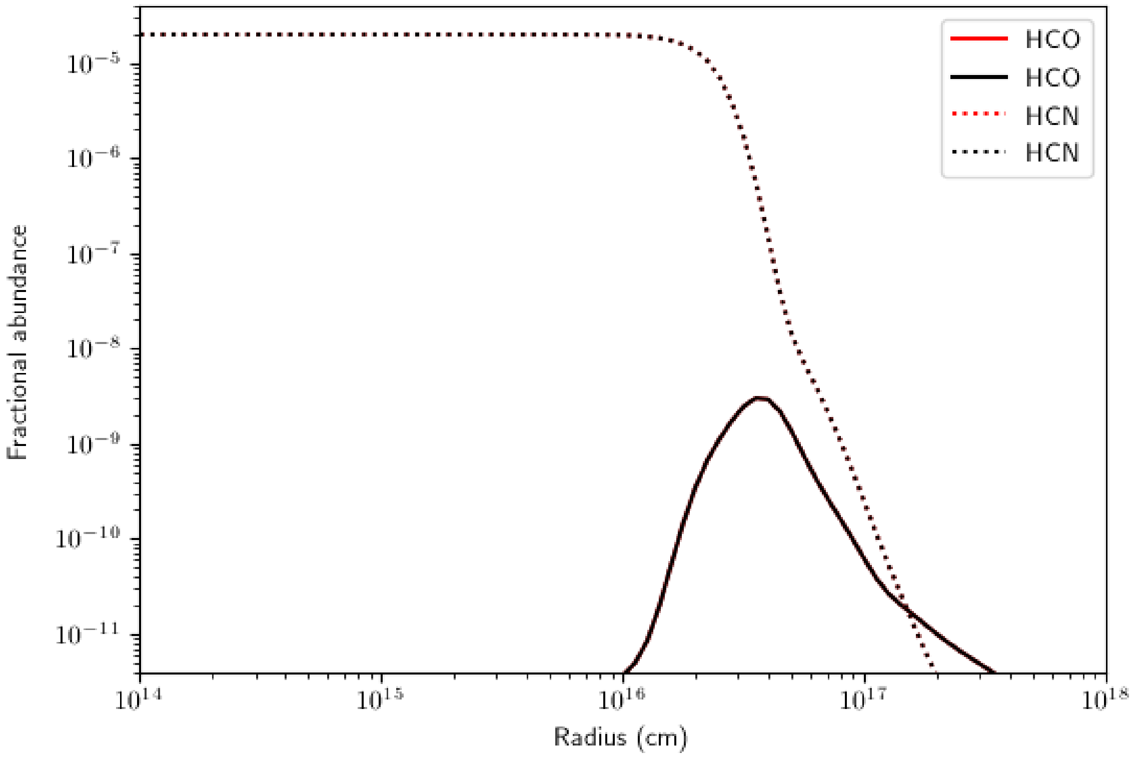

Fig. S14. HCO and HCN profiles throughout the C-rich AGB outflow with a mass-loss rate of 10^{-5} M_{sun}/yr. Red curves relate to the base model, black to the model in which we adopt the fast rate coefficient for the title reaction at low temperatures as discussed in Sect. 4.1. The overlap of red and black curves shows that our new rate coefficient does not change the abundances of HCO and HCN.

References

1. N. A. West, T. J. Millar, M. Van de Sande, E. Rutter, M. A. Blitz, L. Decin and D. E. Heard, *The Astrophysical Journal*, 2019, **885**, 134.
2. C. A. Smith, F. D. Pope, B. Cronin, C. B. Parkes and A. J. Orr-Ewing, *The Journal of Physical Chemistry A*, 2006, **110**, 11645-11653.
3. M. L. Laury, Boesch, S.E., Haken, I., Sinha, P., Wheeler, R.A. and Wilson, A.K. , *Journal of Computational Chemistry*, 2011, **32**, 2239-2347.
4. NIST Computational Chemistry Comparison and Benchmark Database, http://cccbdb.nist.gov/.
5. I. W. M. Smith, *Kinetics and Dynamics of Elementary Gas Reactions*, *Butterworths Monographs in Chemistry and Chemical Engineering*, Butterworths1980.

6. J. O. Hirschfelder, C. F. Curtiss and R. B. Bird, *Molecular theory of gases and liquids,* Wiley, New York, 1964.
7. L. F. Phillips, *Progress in Energy and Combustion Science*, 1992, **18**, 75-90.
8. T. Stoecklin, C. Dateo and D. Clary, *Journal of the Chemical Society, Faraday Transactions*, 1991, **87**, 1667-1679.
9. D. C. Clary, T. S. Stoecklin and A. G. Wickham, *Journal of the Chemical Society, Faraday Transactions*, 1993, **89**, 2185-2191.
10. D. C. Clary, *AIP Conference Proceedings*, 1994, **312**, 405-421.
11. M. Quack and J. Troe, *Berichte der Bunsengesellschaft für Physikalische Chemie*, 1974, **78**, 240-252.
12. J. Troe, *Chemical Physics Letters*, 1985, **122**, 425-430.
13. Y. Georgievskii and S. J. Klippenstein, *The Journal of Chemical Physics*, 2005, **122**, 194103.
14. R. D. Nelson Jr, D. R. Lide Jr and A. A. Maryott, *Selected values of electric dipole moments for molecules in the gas phase*, National Standard Reference Data System1967.
15. R. Thomson and F. Dalby, *Canadian Journal of Physics*, 1968, **46**, 2815-2819.
16. T. N. Olney, N. Cann, G. Cooper and C. Brion, *Chemical physics*, 1997, **223**, 59-98.
17. E. Lemmon, M. McLinden, D. Friend, P. Linstrom and W. Mallard, *NIST Chemistry WebBook, NIST Standard Reference Database Number 69 (National Institute of Standards and Technology, Gaithersburg)*, 2011.
18. B. Niu, D. A. Shirley and Y. Bai, *The Journal of Chemical Physics*, 1993, **98**, 4377-4390.

MESMER .xml Input File


```
<me:title> CN+CH2O</me:title>
  <moleculeList>

        <molecule id="He" description="helium">
            <atomArray>
                <atom elementType="He"/>
            </atomArray>
            <propertyList>
                <property dictRef="me:epsilon">
                    <scalar>10</scalar>
                </property>
                <property dictRef="me:sigma">
                    <scalar>2.55</scalar>
                </property>
                <property dictRef="me:MW">
                    <scalar units="amu">4.0</scalar>
                </property>
            </propertyList>
        </molecule>

        <molecule id="N2" description="nitrogen">
    <atomArray>
      <atom id="a1" elementType="N" />
      <atom id="a2" elementType="N" />
    </atomArray>
    <bondArray>
      <bond atomRefs2="a2 a1" order="3" />
    </bondArray>
    <propertyList>
```

```
      <property dictRef="me:epsilon">
        <scalar>48</scalar>
      </property>
      <property dictRef="me:sigma">
        <scalar>3.9</scalar>
      </property>
      <property dictRef="me:MW">
        <scalar units="amu">28.0</scalar>
      </property>
    </propertyList>
  </molecule>

        <molecule id="Ar" description="argon">
                <atomArray>
                        <atom elementType="Ar"/>
                </atomArray>
                <propertyList>
                        <property dictRef="me:epsilon">
                                <scalar>114</scalar>
                        </property>
                        <property dictRef="me:sigma">
                                <scalar>3.47</scalar>
                        </property>
                        <property dictRef="me:MW">
                                <scalar units="amu">40.0</scalar>
                        </property>
                </propertyList>
        </molecule>

        <molecule id="CN" description="cyano radical">
                <atomArray>
                        <atom id="a1" elementType="C" x3="0.000000" y3="0.000000" z3="0.002649"/>
                        <atom id="a2" elementType="N" x3="0.000000" y3="0.000000" z3="1.155011"/>
                </atomArray>
                <bondArray>
                        <bond atomRefs2="a1 a2" order="3" />
                </bondArray>
   <propertyList>
                        <property title="MW">
        <scalar>26</scalar>
      </property>
                        <property dictRef="me:ZPE">
                                 <scalar units="kJ/mol" >0.0</scalar>
                        </property>
                        <property title="SpinMultiplicity" dictRef="me:spinMultiplicity">
                                <scalar>2 </scalar>
                        </property>
                        <property dictRef="me:frequenciesScaleFactor">
                                <scalar>0.956</scalar>
                        </property>
                        <property title="Vibrational Frequencies" dictRef="me:vibFreqs">
                                <array units="cm-1">2241.98 </array>
                        </property>
                        <property title="Rotational Constants" dictRef="me:rotConsts">
                                <array units="cm-1">1.963 </array>
                        </property>
                        <property title="Symmetry Number" dictRef="me:symmetryNumber">
                                <scalar>1 </scalar>
                        </property>
                </propertyList>
         <me:DOSCMethod>QMRotors</me:DOSCMethod>
                <me:energyTransferModel xsi:type="me:ExponentialDown">
                        <me:deltaEDown bathGas="N2" units="cm-1" >250</me:deltaEDown>
                        <me:deltaEDownTExponent bathGas="N2" referenceTemperature="298" >0.25</me:deltaEDownTExponent>
                        <me:deltaEDown bathGas="Ar" units="cm-1" >200</me:deltaEDown>
                        <me:deltaEDownTExponent bathGas="Ar" referenceTemperature="298" >0.5</me:deltaEDownTExponent>
                        <me:deltaEDown bathGas="He" units="cm-1" >100</me:deltaEDown>
                        <me:deltaEDownTExponent bathGas="He" referenceTemperature="298" >1.0</me:deltaEDownTExponent>
                </me:energyTransferModel>
        </molecule>
```

```xml
<molecule id="CH2O" description="formaldehyde">
        <atomArray>
            <atom id="a1" elementType="C" x3="0.000149" y3="0.000000" z3="0.000050"/>
            <atom id="a2" elementType="H" x3="-0.000141" y3="0.000000" z3="1.103192"/>
            <atom id="a3" elementType="H" x3="0.987034" y3="0.000000" z3="-0.492886"/>
            <atom id="a4" elementType="O" x3="-1.017051" y3="0.000000" z3="-0.629071"/>
        </atomArray>
        <bondArray>
            <bond atomRefs2="a4 a1" order="2"/>
            <bond atomRefs2="a3 a1" order="1"/>
            <bond atomRefs2="a1 a2" order="1"/>
        </bondArray>
        <propertyList>
            <property title="MW">
   <scalar>30</scalar>
  </property>
            <property dictRef="me:ZPE">
                 <scalar units="kJ/mol" >0.0</scalar>
            </property>
            <property title="SpinMultiplicity" dictRef="me:spinMultiplicity">
                <scalar>1 </scalar>
            </property>
            <property dictRef="me:frequenciesScaleFactor">
                <scalar>0.956</scalar>
            </property>
            <property title="Vibrational Frequencies" dictRef="me:vibFreqs">
                <array units="cm-1">1216.33 1275.01 1540.72 1869.43 2948.58 3018.35 </array>
            </property>
            <property title="Rotational Constants" dictRef="me:rotConsts">
                <array units="cm-1">9.492 1.315 1.155 </array>
            </property>
            <property title="Symmetry Number" dictRef="me:symmetryNumber">
                <scalar>2 </scalar>
            </property>
        </propertyList>
    <me:DOSCMethod>QMRotors</me:DOSCMethod>
        <me:energyTransferModel xsi:type="me:ExponentialDown">
            <me:deltaEDown bathGas="N2" units="cm-1" >250</me:deltaEDown>
            <me:deltaEDownTExponent bathGas="N2" referenceTemperature="298" >0.25</me:deltaEDownTExponent>
            <me:deltaEDown bathGas="Ar" units="cm-1" >200</me:deltaEDown>
            <me:deltaEDownTExponent bathGas="Ar" referenceTemperature="298" >0.5</me:deltaEDownTExponent>
            <me:deltaEDown bathGas="He" units="cm-1" >100</me:deltaEDown>
            <me:deltaEDownTExponent bathGas="He" referenceTemperature="298" >1.0</me:deltaEDownTExponent>
        </me:energyTransferModel>
   </molecule>

   <molecule id="HCO" description="formyl radical">
        <atomArray>
            <atom id="a1" elementType="C" x3="-1.147850" y3="0.514116" z3="-0.072588"/>
            <atom id="a2" elementType="H" x3="-2.141312" y3="0.148118" z3="0.293410"/>
            <atom id="a3" elementType="O" x3="-0.118790" y3="0.123654" z3="0.317874"/>
        </atomArray>
        <bondArray>
            <bond atomRefs2="a1 a2" order="1"/>
            <bond atomRefs2="a1 a3" order="2"/>
        </bondArray>
        <propertyList>
            <property title="MW">
   <scalar>29</scalar>
  </property>
            <property dictRef="me:ZPE">
                 <scalar units="kJ/mol" >0.0</scalar>
            </property>
            <property title="SpinMultiplicity" dictRef="me:spinMultiplicity">
                <scalar>2 </scalar>
            </property>
            <property dictRef="me:frequenciesScaleFactor">
                <scalar>0.956</scalar>
            </property>
```

```
<property title="Vibrational Frequencies" dictRef="me:vibFreqs">
        <array units="cm-1">1103.45 1993.06 2729.90 </array>
</property>
<property title="Rotational Constants" dictRef="me:rotConsts">
        <array units="cm-1">24.030 1.515 1.425 </array>
</property>
<property title="Symmetry Number" dictRef="me:symmetryNumber">
        <scalar>1 </scalar>
</property>
</propertyList>
<me:DOSCMethod>QMRotors</me:DOSCMethod>
<me:energyTransferModel xsi:type="me:ExponentialDown">
        <me:deltaEDown bathGas="N2" units="cm-1" >250</me:deltaEDown>
        <me:deltaEDownTExponent bathGas="N2" referenceTemperature="298" >0.25</me:deltaEDownTExponent>
        <me:deltaEDown bathGas="Ar" units="cm-1" >200</me:deltaEDown>
        <me:deltaEDownTExponent bathGas="Ar" referenceTemperature="298" >0.5</me:deltaEDownTExponent>
        <me:deltaEDown bathGas="He" units="cm-1" >100</me:deltaEDown>
        <me:deltaEDownTExponent bathGas="He" referenceTemperature="298" >1.0</me:deltaEDownTExponent>
</me:energyTransferModel>
</molecule>

<molecule id="HCO2" description="formyl radical">
        <atomArray>
                <atom id="a1" elementType="C" x3="-1.147850" y3="0.514116" z3="-0.072588"/>
                <atom id="a2" elementType="H" x3="-2.141312" y3="0.148118" z3="0.293410"/>
                <atom id="a3" elementType="O" x3="-0.118790" y3="0.123654" z3="0.317874"/>
        </atomArray>
        <bondArray>
                <bond atomRefs2="a1 a2" order="1"/>
                <bond atomRefs2="a1 a3" order="2"/>
        </bondArray>
        <propertyList>
                <property title="MW">
  <scalar>29</scalar>
</property>
                <property dictRef="me:ZPE">
                        <scalar units="kJ/mol" >0.00</scalar>
                </property>
                <property title="SpinMultiplicity" dictRef="me:spinMultiplicity">
                        <scalar>2 </scalar>
                </property>
                <property dictRef="me:frequenciesScaleFactor">
                        <scalar>0.956</scalar>
                        <!-- <scalar>1</scalar> -->
                </property>
                <property title="Vibrational Frequencies" dictRef="me:vibFreqs">
                        <array units="cm-1">1103.45 1993.06 2729.90 </array>
                </property>
                <property title="Rotational Constants" dictRef="me:rotConsts">
                        <array units="cm-1">24.030 1.515 1.425 </array>
                </property>
                <property title="Symmetry Number" dictRef="me:symmetryNumber">
                        <scalar>1 </scalar>
                </property>
        </propertyList>
        <me:DOSCMethod>QMRotors</me:DOSCMethod>
                <me:energyTransferModel xsi:type="me:ExponentialDown">
                <me:deltaEDown bathGas="N2" units="cm-1" >250</me:deltaEDown>
                <me:deltaEDownTExponent bathGas="N2" referenceTemperature="298" >0.25</me:deltaEDownTExponent>
                <me:deltaEDown bathGas="Ar" units="cm-1" >200</me:deltaEDown>
                <me:deltaEDownTExponent bathGas="Ar" referenceTemperature="298" >0.5</me:deltaEDownTExponent>
                <me:deltaEDown bathGas="He" units="cm-1" >100</me:deltaEDown>
                <me:deltaEDownTExponent bathGas="He" referenceTemperature="298" >1.0</me:deltaEDownTExponent>
        </me:energyTransferModel>
</molecule>

<molecule id="HCN" description="hydrogen cyanide">
        <atomArray>
                <atom id="a1" elementType="C" x3="-0.500970" y3="0.274463" z3="0.000000"/>
                <atom id="a2" elementType="H" x3="-1.567210" y3="0.274463" z3="0.000000"/>
```

```
                                        <atom id="a3" elementType="N" x3="0.641201" y3="0.274463" z3="0.000000"/>
                        </atomArray>
                        <bondArray>
                                        <bond atomRefs2="a1 a2" order="1"/>
                                        <bond atomRefs2="a1 a3" order="3"/>
                        </bondArray>
                        <propertyList>
                                        <property title="MW">
   <scalar>27</scalar>
 </property>
                                        <property dictRef="me:ZPE">
                                                  <scalar units="kJ/mol" >-163.4</scalar>
                                        </property>
                                        <property title="SpinMultiplicity" dictRef="me:spinMultiplicity">
                                                <scalar>1 </scalar>
                                        </property>
                                        <property dictRef="me:frequenciesScaleFactor">
                                                <scalar>0.956</scalar>
                                                <!-- <scalar>1</scalar> -->
                                        </property>
                                        <property title="Vibrational Frequencies" dictRef="me:vibFreqs">
                                                <array units="cm-1">785.35 785.35 2254.55 3466.42 </array>
                                        </property>
                                        <property title="Rotational Constants" dictRef="me:rotConsts">
                                                <array units="cm-1">1.508 </array>
                                        </property>
                                        <property title="Symmetry Number" dictRef="me:symmetryNumber">
                                                <scalar>1 </scalar>
                                        </property>
                        </propertyList>
                          <me:DOSCMethod>QMRotors</me:DOSCMethod>
                                        <me:energyTransferModel xsi:type="me:ExponentialDown">
                                        <me:deltaEDown bathGas="N2" units="cm-1" >250</me:deltaEDown>
                                        <me:deltaEDownTExponent bathGas="N2" referenceTemperature="298" >0.25</me:deltaEDownTExponent>
                                        <me:deltaEDown bathGas="Ar" units="cm-1" >200</me:deltaEDown>
                                        <me:deltaEDownTExponent bathGas="Ar" referenceTemperature="298" >0.5</me:deltaEDownTExponent>
                                        <me:deltaEDown bathGas="He" units="cm-1" >100</me:deltaEDown>
                                        <me:deltaEDownTExponent bathGas="He" referenceTemperature="298" >1.0</me:deltaEDownTExponent>
                        </me:energyTransferModel>
        </molecule>

        <molecule id="HCN2" description="hydrogen cyanide">
                        <atomArray>
                                        <atom id="a1" elementType="C" x3="-0.500970" y3="0.274463" z3="0.000000"/>
                                        <atom id="a2" elementType="H" x3="-1.567210" y3="0.274463" z3="0.000000"/>
                                        <atom id="a3" elementType="N" x3="0.641201" y3="0.274463" z3="0.000000"/>
                        </atomArray>
                        <bondArray>
                                        <bond atomRefs2="a1 a2" order="1"/>
                                        <bond atomRefs2="a1 a3" order="3"/>
                        </bondArray>
                        <propertyList>
                                        <property title="MW">
   <scalar>27</scalar>
 </property>
                                        <property dictRef="me:ZPE">
                                                  <scalar units="kJ/mol" >-163.4</scalar>
                                        </property>
                                        <property title="SpinMultiplicity" dictRef="me:spinMultiplicity">
                                                <scalar>1 </scalar>
                                        </property>
                                        <property dictRef="me:frequenciesScaleFactor">
                                                <scalar>0.956</scalar>
                                        </property>
                                        <property title="Vibrational Frequencies" dictRef="me:vibFreqs">
                                                <array units="cm-1">785.35 785.35 2254.55 3466.42 </array>
                                        </property>
                                        <property title="Rotational Constants" dictRef="me:rotConsts">
                                                <array units="cm-1">1.508 </array>
                                        </property>
```

```
<property title="Symmetry Number" dictRef="me:symmetryNumber">
<scalar>1 </scalar>
</property>
</propertyList>
<me:DOSCMethod>QMRotors</me:DOSCMethod>
<me:energyTransferModel xsi:type="me:ExponentialDown">
<me:deltaEDown bathGas="N2" units="cm-1" >250</me:deltaEDown>
<me:deltaEDownTExponent bathGas="N2" referenceTemperature="298" >0.25</me:deltaEDownTExponent>
<me:deltaEDown bathGas="Ar" units="cm-1" >200</me:deltaEDown>
<me:deltaEDownTExponent bathGas="Ar" referenceTemperature="298" >0.5</me:deltaEDownTExponent>
<me:deltaEDown bathGas="He" units="cm-1" >100</me:deltaEDown>
<me:deltaEDownTExponent bathGas="He" referenceTemperature="298" >1.0</me:deltaEDownTExponent>
</me:energyTransferModel>
</molecule>


<molecule id="HNC" description="hydrogen isocyanide">
<atomArray>
<atom id="a1" elementType="H" x3="0.000000" y3="0.000000" z3="1.424672"/>
<atom id="a2" elementType="N" x3="0.000000" y3="0.000000" z3="0.426514"/>
<atom id="a3" elementType="C" x3="0.000000" y3="0.000000" z3="-0.735045"/>
</atomArray>
<bondArray>
<bond atomRefs2="a3 a2" order="1"/>
<bond atomRefs2="a2 a1" order="1"/>
</bondArray>
<propertyList>
<property title="MW">
<scalar>27</scalar>
</property>
<property dictRef="me:ZPE">
<scalar units="kJ/mol" >-103.6</scalar>
</property>
<property title="SpinMultiplicity" dictRef="me:spinMultiplicity">
<scalar>1 </scalar>
</property>
<property dictRef="me:frequenciesScaleFactor">
<scalar>0.956</scalar>
</property>
<property title="Vibrational Frequencies" dictRef="me:vibFreqs">
<array units="cm-1">528.7255 528.7255 2147.7615 3811.6074 </array>
</property>
<property title="Rotational Constants" dictRef="me:rotConsts">
<array units="cm-1">1.531 </array>
</property>
<property title="Symmetry Number" dictRef="me:symmetryNumber">
<scalar>1 </scalar>
</property>
</propertyList>
<me:DOSCMethod>QMRotors</me:DOSCMethod>
<me:energyTransferModel xsi:type="me:ExponentialDown">
<me:deltaEDown bathGas="N2" units="cm-1" >250</me:deltaEDown>
<me:deltaEDownTExponent bathGas="N2" referenceTemperature="298" >0.25</me:deltaEDownTExponent>
<me:deltaEDown bathGas="Ar" units="cm-1" >200</me:deltaEDown>
<me:deltaEDownTExponent bathGas="Ar" referenceTemperature="298" >0.5</me:deltaEDownTExponent>
<me:deltaEDown bathGas="He" units="cm-1" >100</me:deltaEDown>
<me:deltaEDownTExponent bathGas="He" referenceTemperature="298" >1.0</me:deltaEDownTExponent>
</me:energyTransferModel>
</molecule>


<molecule id="NCO" description="isocyanato radical">
<atomArray>
<atom id="a1" elementType="C" x3="-0.828647" y3="0.393795" z3="0.074099"/>
<atom id="a2" elementType="O" x3="0.338825" y3="0.393795" z3="0.071475"/>
<atom id="a3" elementType="N" x3="-2.051211" y3="0.393795" z3="0.076847"/>
</atomArray>
<bondArray>
<bond atomRefs2="a2 a1" order="1"/>
<bond atomRefs2="a1 a3" order="3"/>
```

```
                </bondArray>
                <propertyList>
                                <property title="MW">
   <scalar>42</scalar>
</property>
                                <property dictRef="me:ZPE">
                                                <scalar units="kJ/mol" >174.4</scalar>
                                </property>
                                <property title="SpinMultiplicity" dictRef="me:spinMultiplicity">
                                                <scalar>2 </scalar>
                                </property>
                                <property dictRef="me:frequenciesScaleFactor">
                                                <scalar>0.956</scalar>
                                                <!-- <scalar>1</scalar> -->
                                </property>
                                <property title="Vibrational Frequencies" dictRef="me:vibFreqs">
                                                <array units="cm-1">533.01 614.67 1326.97 2039.94 </array>
                                </property>
                                <property title="Rotational Constants" dictRef="me:rotConsts">
                                                <array units="cm-1">0.395 </array>
                                </property>
                                <property title="Symmetry Number" dictRef="me:symmetryNumber">
                                                <scalar>1 </scalar>
                                </property>
                </propertyList>
                  <me:DOSCMethod>QMRotors</me:DOSCMethod>
                                <me:energyTransferModel xsi:type="me:ExponentialDown">
                                <me:deltaEDown bathGas="N2" units="cm-1" >250</me:deltaEDown>
                                <me:deltaEDownTExponent bathGas="N2" referenceTemperature="298" >0.25</me:deltaEDownTExponent>
                                <me:deltaEDown bathGas="Ar" units="cm-1" >200</me:deltaEDown>
                                <me:deltaEDownTExponent bathGas="Ar" referenceTemperature="298" >0.5</me:deltaEDownTExponent>
                                <me:deltaEDown bathGas="He" units="cm-1" >100</me:deltaEDown>
                                <me:deltaEDownTExponent bathGas="He" referenceTemperature="298" >1.0</me:deltaEDownTExponent>
                </me:energyTransferModel>
     </molecule>

     <molecule id="CH2_triplet" description="triplet methylene">
                <atomArray>
                                <atom id="a1" elementType="C" x3="-1.150644" y3="0.882015" z3="0.000000"/>
                                <atom id="a2" elementType="H" x3="-0.583564" y3="-0.031638" z3="0.000000"/>
                                <atom id="a3" elementType="H" x3="-2.201094" y3="1.112187" z3="0.000000"/>
                </atomArray>
                <bondArray>
                                <bond atomRefs2="a1 a2" order="1"/>
                                <bond atomRefs2="a1 a3" order="1"/>
                </bondArray>
                <propertyList>
                                <property title="MW">
   <scalar>14</scalar>
</property>
                                <property dictRef="me:ZPE">
                                                <scalar units="kJ/mol" >0.0</scalar>
                                </property>
                                <property title="SpinMultiplicity" dictRef="me:spinMultiplicity">
                                                <scalar>3 </scalar>
                                </property>
                                <property dictRef="me:frequenciesScaleFactor">
                                                <scalar>0.956</scalar>
                                                <!-- <scalar>1</scalar> -->
                                </property>
                                <property title="Vibrational Frequencies" dictRef="me:vibFreqs">
                                                <array units="cm-1">1096.31 3177.82 3396.73 </array>
                                </property>
                                <property title="Rotational Constants" dictRef="me:rotConsts">
                                                <array units="cm-1">55.716 8.518 7.388 </array>
                                </property>
                                <property title="Symmetry Number" dictRef="me:symmetryNumber">
                                                <scalar>2 </scalar>
                                </property>
                </propertyList>
```

```
          <me:DOSCMethod>QMRotors</me:DOSCMethod>
                    <me:energyTransferModel xsi:type="me:ExponentialDown">
                    <me:deltaEDown bathGas="N2" units="cm-1" >250</me:deltaEDown>
                    <me:deltaEDownTExponent bathGas="N2" referenceTemperature="298" >0.25</me:deltaEDownTExponent>
                    <me:deltaEDown bathGas="Ar" units="cm-1" >200</me:deltaEDown>
                    <me:deltaEDownTExponent bathGas="Ar" referenceTemperature="298" >0.5</me:deltaEDownTExponent>
                    <me:deltaEDown bathGas="He" units="cm-1" >100</me:deltaEDown>
                    <me:deltaEDownTExponent bathGas="He" referenceTemperature="298" >1.0</me:deltaEDownTExponent>
          </me:energyTransferModel>
     </molecule>

     <molecule id="NCCOH" description="formyl cyanide">
          <atomArray>
                    <atom id="a1" elementType="O" x3="-0.001567" y3="0.000000" z3="-0.001708"/>
                    <atom id="a2" elementType="C" x3="0.003297" y3="0.000000" z3="1.190616"/>
                    <atom id="a3" elementType="H" x3="0.920955" y3="0.000000" z3="1.792847"/>
                    <atom id="a4" elementType="C" x3="-1.243050" y3="-0.000002" z3="1.981326"/>
                    <atom id="a5" elementType="N" x3="-2.182272" y3="-0.000003" z3="2.637437"/>
          </atomArray>
          <bondArray>
                    <bond atomRefs2="a1 a2" order="2"/>
                    <bond atomRefs2="a2 a3" order="1"/>
                    <bond atomRefs2="a2 a4" order="1"/>
                    <bond atomRefs2="a4 a5" order="3"/>
          </bondArray>
          <propertyList>
                    <property title="MW">
    <scalar>55</scalar>
  </property>
                    <property dictRef="me:ZPE">
                              <scalar units="kJ/mol" >-66.7</scalar>
                    </property>
                    <property title="SpinMultiplicity" dictRef="me:spinMultiplicity">
                              <scalar>1 </scalar>
                    </property>
                    <property dictRef="me:frequenciesScaleFactor">
                              <scalar>0.956</scalar>
                    </property>
                    <property title="Vibrational Frequencies" dictRef="me:vibFreqs">
                    <array units="cm-1">235.33 310.08 630.65 932.19 1016.90 1415.08 1853.22 2407.28 3051.78 </array>
                    </property>
                    <property title="Rotational Constants" dictRef="me:rotConsts">
                              <array units="cm-1">2.283 0.168 0.157 </array>
                    </property>
                    <property title="Symmetry Number" dictRef="me:symmetryNumber">
                              <scalar>1 </scalar>
                    </property>
          </propertyList>
          <me:DOSCMethod>QMRotors</me:DOSCMethod>
                    <me:energyTransferModel xsi:type="me:ExponentialDown">
                    <me:deltaEDown bathGas="N2" units="cm-1" >250</me:deltaEDown>
                    <me:deltaEDownTExponent bathGas="N2" referenceTemperature="298" >0.25</me:deltaEDownTExponent>
                    <me:deltaEDown bathGas="Ar" units="cm-1" >200</me:deltaEDown>
                    <me:deltaEDownTExponent bathGas="Ar" referenceTemperature="298" >0.5</me:deltaEDownTExponent>
                    <me:deltaEDown bathGas="He" units="cm-1" >100</me:deltaEDown>
                    <me:deltaEDownTExponent bathGas="He" referenceTemperature="298" >1.0</me:deltaEDownTExponent>
          </me:energyTransferModel>
     </molecule>

     <molecule id="H" description="hydrogen">
          <atom elementType="H" />
          <propertyList>
                    <property dictRef="me:ZPE">
                              <scalar units="kJ/mol">0.0</scalar>
                    </property>
                    <property dictRef="me:MW">
                              <scalar units="amu">1.0</scalar>
                    </property>
          </propertyList>
</molecule>
```

<molecule id="vdW" description="CH2OCN van der Waals complex">
<atomArray>
<atom id="a1" elementType="C" x3="-0.080300" y3="0.141554" z3="0.088613"/>
<atom id="a2" elementType="H" x3="0.349647" y3="-0.096109" z3="1.070309"/>
<atom id="a3" elementType="H" x3="0.547290" y3="0.037724" z3="-0.805447"/>
<atom id="a4" elementType="O" x3="-1.224333" y3="0.509627" z3="0.007825"/>
<atom id="a5" elementType="C" x3="-1.544984" y3="0.835658" z3="-2.128061"/>
<atom id="a6" elementType="N" x3="-0.844325" y3="0.709161" z3="-3.037761"/>
</atomArray>
<bondArray>
<bond atomRefs2="a6 a5" order="3"/>
<bond atomRefs2="a3 a1" order="1"/>
<bond atomRefs2="a4 a1" order="2"/>
<bond atomRefs2="a1 a2" order="1"/>
</bondArray>
<propertyList>
<property title="MW">
<scalar>56</scalar>
</property>
<property dictRef="me:ZPE">
<scalar units="kJ/mol" >-13.3</scalar>
</property>
<property title="SpinMultiplicity" dictRef="me:spinMultiplicity">
<scalar>2 </scalar>
</property>
<property dictRef="me:frequenciesScaleFactor">
<scalar>0.956</scalar>
</property>
<property title="Vibrational Frequencies" dictRef="me:vibFreqs">
<array units="cm-1">116.77 129.48 262.32 301.72 306.47 1229.07 1264.41 1513.73 1821.96 2212.08 3004.57 3103.24 </array>
</property>
<property title="Rotational Constants" dictRef="me:rotConsts">
<array units="cm-1">0.986 0.151 0.131 </array>
</property>
<property title="Symmetry Number" dictRef="me:symmetryNumber">
<scalar>1 </scalar>
</property>
</propertyList>
<!-- <me:DOSCMethod>ClassicalRotors</me:DOSCMethod> -->
<me:DOSCMethod>QMRotors</me:DOSCMethod>
<me:energyTransferModel xsi:type="me:ExponentialDown">
<me:deltaEDown bathGas="N2" units="cm-1" >250</me:deltaEDown>
<me:deltaEDownTExponent bathGas="N2" referenceTemperature="298" >0.25</me:deltaEDownTExponent>
<me:deltaEDown bathGas="Ar" units="cm-1" >200</me:deltaEDown>
<me:deltaEDownTExponent bathGas="Ar" referenceTemperature="298" >0.5</me:deltaEDownTExponent>
<me:deltaEDown bathGas="He" units="cm-1" >100</me:deltaEDown>
<me:deltaEDownTExponent bathGas="He" referenceTemperature="298" >1.0</me:deltaEDownTExponent>
</me:energyTransferModel>
</molecule>

<molecule id="TS_HCN" description="TS_HCN: -> HCO + HCN">
<atomArray>
<atom id="a1" elementType="C" x3="0.171653" y3="0.258723" z3="-0.232532"/>
<atom id="a2" elementType="H" x3="1.261627" y3="0.375361" z3="-0.022772"/>
<atom id="a3" elementType="H" x3="-0.394427" y3="1.197558" z3="-0.352716"/>
<atom id="a4" elementType="O" x3="-0.333476" y3="-0.815769" z3="-0.318145"/>
<atom id="a5" elementType="C" x3="3.144881" y3="-0.639971" z3="0.387528"/>
<atom id="a6" elementType="N" x3="4.271679" y3="-0.628389" z3="0.630964"/>
</atomArray>
<bondArray>
<bond atomRefs2="a3 a1" order="1"/>
<bond atomRefs2="a4 a1" order="2"/>
<bond atomRefs2="a1 a2" order="1"/>
<bond atomRefs2="a5 a6" order="3"/>
</bondArray>
<propertyList>
<property title="MW">
<scalar>56</scalar>
</property>

<property dictRef="me:ZPE">

<!-- <scalar units="kJ/mol" lower="-5" upper="15" stepsize="0.01">3.9670365</scalar> -->
<scalar units="kJ/mol" lower="-5" upper="15" stepsize="0.01">1.70365</scalar>
<!-- <scalar units="kJ/mol" >-0.62</scalar> -->
</property>

<property title="SpinMultiplicity" dictRef="me:spinMultiplicity">
<scalar>2 </scalar>
</property>
<property dictRef="me:frequenciesScaleFactor">
<scalar>0.956</scalar>
<!-- <scalar>1</scalar> -->
</property>
<property title="Vibrational Frequencies" dictRef="me:vibFreqs">
<array units="cm-1">23.30 35.51 50.74 91.04 1196.62 1227.96 1509.28 1876.03 2226.64 2725.70 2996.81 </array>
</property>

<property title="ImaginaryFrequency" dictRef="me:imFreqs">
<scalar units="cm-1" lower="100.19" upper="2794.5" stepsize="1">804</scalar>
</property>

<!-- <property dictRef="me:imFreqs"> -->
<!-- <scalar units="cm-1">214.66</scalar> -->
<!-- </property> -->

<property title="Rotational Constants" dictRef="me:rotConsts">
<array units="cm-1">1.475 0.074 0.071 </array>
</property>
<property title="Symmetry Number" dictRef="me:symmetryNumber">
<scalar>1 </scalar>
</property>
</propertyList>
<!-- <me:DOSCMethod>ClassicalRotors</me:DOSCMethod> -->
<me:DOSCMethod>QMRotors</me:DOSCMethod>
</molecule>

<molecule id="TS_HNC" description="TS_HCN: -> HCO + HNC">
<atomArray>
<atom id="a1" elementType="C" x3="0.211360" y3="-0.018769" z3="0.603110"/>
<atom id="a2" elementType="H" x3="1.213353" y3="-0.110764" z3="1.123255"/>
<atom id="a3" elementType="H" x3="0.248204" y3="-0.012313" z3="-0.504038"/>
<atom id="a4" elementType="O" x3="-0.808838" y3="0.064937" z3="1.203901"/>
<atom id="a5" elementType="N" x3="2.883279" y3="-0.263386" z3="1.816820"/>
<atom id="a6" elementType="C" x3="3.916106" y3="-0.359726" z3="2.344075"/>
</atomArray>
<bondArray>
<bond atomRefs2="a3 a1" order="1"/>
<bond atomRefs2="a1 a2" order="1"/>
<bond atomRefs2="a1 a4" order="2"/>
<bond atomRefs2="a5 a6" order="3"/>
</bondArray>
<propertyList>
<property title="MW">
<scalar>56</scalar>
</property>
<property dictRef="me:ZPE">
<scalar units="kJ/mol" >3.97</scalar>
</property>
<property title="SpinMultiplicity" dictRef="me:spinMultiplicity">
<scalar>2 </scalar>
</property>
<property dictRef="me:frequenciesScaleFactor">
<scalar>0.956</scalar>
<!-- <scalar>1</scalar> -->
</property>
<property title="Vibrational Frequencies" dictRef="me:vibFreqs">
<array units="cm-1">48.64 67.45 134.49 266.03 1209.13 1212.25 1479.93 1841.45 2080.07 2325.26 2932.90 </array>
</property>
<property dictRef="me:imFreqs">

```
                <scalar units="cm-1">222.67 </scalar>
            </property>
            <property title="Rotational Constants" dictRef="me:rotConsts">
                <array units="cm-1">2.122 0.076 0.073 </array>
            </property>
            <property title="Symmetry Number" dictRef="me:symmetryNumber">
                <scalar>1 </scalar>
            </property>
        </propertyList>
        <!-- <me:DOSCMethod>ClassicalRotors</me:DOSCMethod> -->
         <me:DOSCMethod>QMRotors</me:DOSCMethod>
    </molecule>

    <molecule id="TS_chain" description="TS_chain: vdW -> CH2OCN chain">
        <atomArray>
            <atom id="a1" elementType="C" x3="-0.011628" y3="0.181347" z3="0.027036"/>
            <atom id="a2" elementType="H" x3="0.029198" y3="-0.298051" z3="1.003040"/>
            <atom id="a3" elementType="H" x3="0.798510" y3="0.065685" z3="-0.690982"/>
            <atom id="a4" elementType="O" x3="-1.034349" y3="0.820332" z3="-0.276999"/>
            <atom id="a5" elementType="C" x3="-1.501145" y3="0.632986" z3="-1.896234"/>
            <atom id="a6" elementType="N" x3="-1.077588" y3="0.735315" z3="-2.970385"/>
        </atomArray>
        <bondArray>
            <bond atomRefs2="a6 a5" order="2"/>
            <bond atomRefs2="a5 a4" order="1"/>
            <bond atomRefs2="a3 a1" order="1"/>
            <bond atomRefs2="a4 a1" order="1"/>
            <bond atomRefs2="a1 a2" order="1"/>
        </bondArray>
        <propertyList>
            <property title="MW">
   <scalar>56</scalar>
</property>
            <property dictRef="me:ZPE">
                 <scalar units="kJ/mol" >32.9</scalar>
            </property>
            <property title="SpinMultiplicity" dictRef="me:spinMultiplicity">
                <scalar>2 </scalar>
            </property>
            <property dictRef="me:frequenciesScaleFactor">
                <scalar>0.956</scalar>
            </property>
            <property title="Vibrational Frequencies" dictRef="me:vibFreqs">
        <array units="cm-1">114.62 180.30 455.82 522.25 1064.12 1230.19 1379.39 1546.50 2177.92 3075.09 3204.88 </array>
            </property>
            <property dictRef="me:imFreqs">
                <scalar units="cm-1">850.04 </scalar>
            </property>

            <property title="Rotational Constants" dictRef="me:rotConsts">
                <array units="cm-1">1.204 0.175 0.156 </array>
            </property>
            <property title="Symmetry Number" dictRef="me:symmetryNumber">
                <scalar>1 </scalar>
            </property>
        </propertyList>
        <!-- <me:DOSCMethod>ClassicalRotors</me:DOSCMethod> -->
         <me:DOSCMethod>QMRotors</me:DOSCMethod>
    </molecule>

    <molecule id="Int1" description="CH2OCN chain">
        <atomArray>
            <atom id="a1" elementType="C" x3="-0.006494" y3="-0.013970" z3="-0.001597"/>
            <atom id="a2" elementType="H" x3="0.012237" y3="0.011278" z3="1.072248"/>
            <atom id="a3" elementType="H" x3="0.867871" y3="0.004799" z3="-0.630944"/>
            <atom id="a4" elementType="O" x3="-1.160699" y3="0.558096" z3="-0.515661"/>
            <atom id="a5" elementType="C" x3="-1.216207" y3="0.712035" z3="-1.796399"/>
            <atom id="a6" elementType="N" x3="-1.293711" y3="0.865376" z3="-2.932170"/>
        </atomArray>
        <bondArray>
```

```
                <bond atomRefs2="a6 a5" order="3"/>
                <bond atomRefs2="a5 a4" order="1"/>
                <bond atomRefs2="a3 a1" order="1"/>
                <bond atomRefs2="a4 a1" order="1"/>
                <bond atomRefs2="a1 a2" order="1"/>
            </bondArray>
            <propertyList>
                <property title="MW">
  <scalar>56</scalar>
</property>
                <property dictRef="me:ZPE">

                    <scalar units="kJ/mol" >-119.2</scalar>
                </property>
                <property title="SpinMultiplicity" dictRef="me:spinMultiplicity">
                    <scalar>2 </scalar>
                </property>
                <property dictRef="me:frequenciesScaleFactor">
                    <scalar>0.956</scalar>
                </property>
                <property title="Vibrational Frequencies" dictRef="me:vibFreqs">
    <array units="cm-1">229.79 247.11 526.28 557.04 641.13 975.91 1189.54 1281.11 1469.33 2432.96 3187.01 3347.42 </array>
                </property>
                <property title="Rotational Constants" dictRef="me:rotConsts">
                    <array units="cm-1">1.714 0.186 0.168 </array>
                </property>
                <property title="Symmetry Number" dictRef="me:symmetryNumber">
                    <scalar>1 </scalar>
                </property>
            </propertyList>
            <!-- <me:DOSCMethod>ClassicalRotors</me:DOSCMethod> -->
              <me:DOSCMethod>QMRotors</me:DOSCMethod>
            <me:energyTransferModel xsi:type="me:ExponentialDown">
                <me:deltaEDown bathGas="N2" units="cm-1" >250</me:deltaEDown>
                <me:deltaEDownTExponent bathGas="N2" referenceTemperature="298" >0.25</me:deltaEDownTExponent>
                <me:deltaEDown bathGas="Ar" units="cm-1" >200</me:deltaEDown>
                <me:deltaEDownTExponent bathGas="Ar" referenceTemperature="298" >0.5</me:deltaEDownTExponent>
                <me:deltaEDown bathGas="He" units="cm-1" >100</me:deltaEDown>
                <me:deltaEDownTExponent bathGas="He" referenceTemperature="298" >1.0</me:deltaEDownTExponent>
            </me:energyTransferModel>
    </molecule>

    <molecule id="TS1/2" description="TS1/2: CH2OCN chain -> cyclo">
            <atomArray>
                <atom id="a1" elementType="C" x3="0.087995" y3="0.149432" z3="-0.305293"/>
                <atom id="a2" elementType="H" x3="0.019071" y3="-0.842825" z3="0.110587"/>
                <atom id="a3" elementType="H" x3="0.957427" y3="0.779412" z3="-0.207829"/>
                <atom id="a4" elementType="O" x3="-1.149901" y3="0.838565" z3="-0.442252"/>
                <atom id="a5" elementType="C" x3="-0.937155" y3="0.471047" z3="-1.688285"/>
                <atom id="a6" elementType="N" x3="-1.000889" y3="0.281186" z3="-2.843567"/>
            </atomArray>
            <bondArray>
                <bond atomRefs2="a6 a5" order="2"/>
                <bond atomRefs2="a5 a4" order="1"/>
                <bond atomRefs2="a5 a1" order="1"/>
                <bond atomRefs2="a4 a1" order="1"/>
                <bond atomRefs2="a1 a3" order="1"/>
                <bond atomRefs2="a1 a2" order="1"/>
            </bondArray>
            <propertyList>
                <property title="MW">
  <scalar>56</scalar>
</property>
                <property dictRef="me:ZPE">
                    <scalar units="kJ/mol" >-44.2</scalar>
                </property>
                <property title="SpinMultiplicity" dictRef="me:spinMultiplicity">
                    <scalar>2 </scalar>
                </property>
                <property dictRef="me:frequenciesScaleFactor">
```

```
                        <scalar>0.956</scalar>
                    </property>
                    <property title="Vibrational Frequencies" dictRef="me:vibFreqs">
            <array units="cm-1">372.58 426.23 606.23 809.50 992.89 1131.96 1142.48 1465.74 2100.92 3166.72 3321.35 </array>
                    </property>
                    <property dictRef="me:imFreqs">
                        <scalar units="cm-1">781.10 </scalar>
                    </property>
                    <property title="Rotational Constants" dictRef="me:rotConsts">
                        <array units="cm-1">0.956 0.249 0.206 </array>
                    </property>
                    <property title="Symmetry Number" dictRef="me:symmetryNumber">
                        <scalar>1 </scalar>
                    </property>
            </propertyList>
             <me:DOSCMethod>QMRotors</me:DOSCMethod>
            <!-- <me:DOSCMethod>ClassicalRotors</me:DOSCMethod> -->
    </molecule>

    <molecule id="Int2" description="CH2OCN cyclo">
            <atomArray>
                    <atom id="a1" elementType="C" x3="-2.093436" y3="0.802871" z3="0.072428"/>
                    <atom id="a2" elementType="H" x3="-1.921142" y3="-0.265184" z3="0.075484"/>
                    <atom id="a3" elementType="H" x3="-1.928044" y3="1.343344" z3="-0.850046"/>
                    <atom id="a4" elementType="O" x3="-1.650955" y3="1.493290" z3="1.269105"/>
                    <atom id="a5" elementType="C" x3="-2.987503" y3="1.372780" z3="1.069571"/>
                    <atom id="a6" elementType="N" x3="-4.071062" y3="1.654352" z3="1.567041"/>
            </atomArray>
            <bondArray>
                    <bond atomRefs2="a3 a1" order="1"/>
                    <bond atomRefs2="a1 a2" order="1"/>
                    <bond atomRefs2="a1 a5" order="1"/>
                    <bond atomRefs2="a1 a4" order="1"/>
                    <bond atomRefs2="a5 a4" order="1"/>
                    <bond atomRefs2="a5 a6" order="2"/>
            </bondArray>
            <propertyList>
                    <property title="MW">
   <scalar>56</scalar>
 </property>
                    <property dictRef="me:ZPE">
                         <scalar units="kJ/mol" >-81.2</scalar>
                    </property>
                    <property title="SpinMultiplicity" dictRef="me:spinMultiplicity">
                        <scalar>2 </scalar>
                    </property>
                    <property dictRef="me:frequenciesScaleFactor">
                        <scalar>0.956</scalar>
                    </property>
                    <property title="Vibrational Frequencies" dictRef="me:vibFreqs">
    <array units="cm-1">428.90 437.41 778.54 899.19 986.81 1110.91 1125.46 1186.64 1498.40 1814.21 3145.08 3251.99 </array>
                    </property>
                    <property title="Rotational Constants" dictRef="me:rotConsts">
                        <array units="cm-1">0.900 0.274 0.219 </array>
                    </property>
                    <property title="Symmetry Number" dictRef="me:symmetryNumber">
                        <scalar>1 </scalar>
                    </property>
            </propertyList>
             <me:DOSCMethod>QMRotors</me:DOSCMethod>
            <!-- <me:DOSCMethod>ClassicalRotors</me:DOSCMethod> -->
            <me:energyTransferModel xsi:type="me:ExponentialDown">
                    <me:deltaEDown bathGas="N2" units="cm-1" >250</me:deltaEDown>
                    <me:deltaEDownTExponent bathGas="N2" referenceTemperature="298" >0.25</me:deltaEDownTExponent>
                    <me:deltaEDown bathGas="Ar" units="cm-1" >200</me:deltaEDown>
                    <me:deltaEDownTExponent bathGas="Ar" referenceTemperature="298" >0.5</me:deltaEDownTExponent>
                    <me:deltaEDown bathGas="He" units="cm-1" >100</me:deltaEDown>
                    <me:deltaEDownTExponent bathGas="He" referenceTemperature="298" >1.0</me:deltaEDownTExponent>
            </me:energyTransferModel>
    </molecule>
```

```
<molecule id="TS2/3" description="TS1/2: CH2OCN cyclo -> NCCH2O">
        <atomArray>
                <atom id="a1" elementType="C" x3="-2.056589" y3="0.835541" z3="0.128585"/>
                <atom id="a2" elementType="H" x3="-1.938468" y3="-0.245655" z3="0.088576"/>
                <atom id="a3" elementType="H" x3="-1.942864" y3="1.347838" z3="-0.824917"/>
                <atom id="a4" elementType="O" x3="-1.512457" y3="1.475867" z3="1.242926"/>
                <atom id="a5" elementType="C" x3="-3.132774" y3="1.317846" z3="0.974976"/>
                <atom id="a6" elementType="N" x3="-4.068989" y3="1.670017" z3="1.593437"/>
        </atomArray>
        <bondArray>
                <bond atomRefs2="a3 a1" order="1"/>
                <bond atomRefs2="a2 a1" order="1"/>
                <bond atomRefs2="a1 a5" order="1"/>
                <bond atomRefs2="a1 a4" order="1"/>
                <bond atomRefs2="a5 a4" order="1"/>
                <bond atomRefs2="a5 a6" order="2"/>
        </bondArray>
        <propertyList>
                <property title="MW">
  <scalar>56</scalar>
</property>
                <property dictRef="me:ZPE">
                         <scalar units="kJ/mol" >-62.5</scalar>
                </property>
                <property title="SpinMultiplicity" dictRef="me:spinMultiplicity">
                        <scalar>2 </scalar>
                </property>
                <property dictRef="me:frequenciesScaleFactor">
                        <scalar>0.956</scalar>
                </property>
                <property title="Vibrational Frequencies" dictRef="me:vibFreqs">
        <array units="cm-1">364.76 387.84 866.28 1012.58 1134.09 1168.99 1254.56 1511.91 2015.12 3085.85 3167.97 </array>
                </property>
                <property dictRef="me:imFreqs">
                        <scalar units="cm-1">661.62 </scalar>
                </property>
                <property title="Rotational Constants" dictRef="me:rotConsts">
                        <array units="cm-1">0.898 0.254 0.206 </array>
                </property>
                <property title="Symmetry Number" dictRef="me:symmetryNumber">
                        <scalar>1 </scalar>
                </property>
        </propertyList>
         <me:DOSCMethod>QMRotors</me:DOSCMethod>
        <!-- <me:DOSCMethod>ClassicalRotors</me:DOSCMethod> -->
</molecule>

<molecule id="Int3" description="NCCH2O">
        <atomArray>
                <atom id="a1" elementType="C" x3="2.101952" y3="0.268975" z3="-0.023397"/>
                <atom id="a2" elementType="H" x3="2.431188" y3="-0.777283" z3="-0.091071"/>
                <atom id="a3" elementType="H" x3="2.431160" y3="0.733486" z3="-0.963342"/>
                <atom id="a4" elementType="C" x3="0.627319" y3="0.316157" z3="0.058210"/>
                <atom id="a5" elementType="N" x3="-0.516023" y3="0.343914" z3="0.106033"/>
                <atom id="a6" elementType="O" x3="2.755029" y3="0.866591" z3="1.011369"/>
        </atomArray>
        <bondArray>
                <bond atomRefs2="a3 a1" order="1"/>
                <bond atomRefs2="a2 a1" order="1"/>
                <bond atomRefs2="a1 a4" order="1"/>
                <bond atomRefs2="a1 a6" order="1"/>
                <bond atomRefs2="a4 a5" order="3"/>
        </bondArray>
        <propertyList>
                <property title="MW">
  <scalar>56</scalar>
</property>
                <property dictRef="me:ZPE">
                         <scalar units="kJ/mol" >-153.4</scalar>
```

```
				</property>
				<property title="SpinMultiplicity" dictRef="me:spinMultiplicity">
					<scalar>2 </scalar>
				</property>
				<property dictRef="me:frequenciesScaleFactor">
					<scalar>0.956</scalar>
				</property>
				<property title="Vibrational Frequencies" dictRef="me:vibFreqs">
	<array units="cm-1">232.38 341.30 598.76 669.20 913.72 1093.42 1182.86 1344.07 1391.48 2424.50 3000.68 3024.26 </array>
				</property>
				<property title="Rotational Constants" dictRef="me:rotConsts">
					<array units="cm-1">1.386 0.164 0.151 </array>
				</property>
				<property title="Symmetry Number" dictRef="me:symmetryNumber">
					<scalar>1 </scalar>
				</property>
			</propertyList>
			  <me:DOSCMethod>QMRotors</me:DOSCMethod>
			<!-- <me:DOSCMethod>ClassicalRotors</me:DOSCMethod> -->
			<me:energyTransferModel xsi:type="me:ExponentialDown">
				<me:deltaEDown bathGas="N2" units="cm-1" >250</me:deltaEDown>
				<me:deltaEDownTExponent bathGas="N2" referenceTemperature="298" >0.25</me:deltaEDownTExponent>
				<me:deltaEDown bathGas="Ar" units="cm-1" >200</me:deltaEDown>
				<me:deltaEDownTExponent bathGas="Ar" referenceTemperature="298" >0.5</me:deltaEDownTExponent>
				<me:deltaEDown bathGas="He" units="cm-1" >100</me:deltaEDown>
				<me:deltaEDownTExponent bathGas="He" referenceTemperature="298" >1.0</me:deltaEDownTExponent>
			</me:energyTransferModel>
	</molecule>

	<molecule id="TS3/P" description="TS3/P: NCCH2O -> NCCOH + H">
			<atomArray>
				<atom id="a1" elementType="C" x3="2.090004" y3="0.643104" z3="0.037604"/>
				<atom id="a2" elementType="H" x3="2.388596" y3="-1.154971" z3="-0.174355"/>
				<atom id="a3" elementType="H" x3="2.492120" y3="0.706080" z3="-0.982975"/>
				<atom id="a4" elementType="C" x3="0.632023" y3="0.397376" z3="0.097142"/>
				<atom id="a5" elementType="N" x3="-0.500558" y3="0.222918" z3="0.101730"/>
				<atom id="a6" elementType="O" x3="2.728438" y3="0.937335" z3="1.018657"/>
			</atomArray>
			<bondArray>
				<bond atomRefs2="a3 a1" order="1"/>
				<bond atomRefs2="a1 a4" order="1"/>
				<bond atomRefs2="a1 a6" order="2"/>
				<bond atomRefs2="a4 a5" order="3"/>
			</bondArray>
			<propertyList>
				<property title="MW">
   <scalar>56</scalar>
</property>
				<property dictRef="me:ZPE">
					  <scalar units="kJ/mol" >-41.0</scalar>
				</property>
				<property title="SpinMultiplicity" dictRef="me:spinMultiplicity">
					<scalar>2 </scalar>
				</property>
				<property dictRef="me:frequenciesScaleFactor">
					<scalar>0.956</scalar>
				</property>
				<property title="Vibrational Frequencies" dictRef="me:vibFreqs">
			<array units="cm-1">234.77 284.02 429.77 469.15 627.39 922.07 1015.67 1393.04 1721.47 2403.72 3029.89 </array>
				</property>
				<property dictRef="me:imFreqs">
					<scalar units="cm-1">927.99 </scalar>
				</property>
				<property title="Rotational Constants" dictRef="me:rotConsts">
					<array units="cm-1">1.451 0.162 0.153 </array>
				</property>
				<property title="Symmetry Number" dictRef="me:symmetryNumber">
					<scalar>1 </scalar>
				</property>
			</propertyList>
```

```
<me:DOSCMethod>QMRotors</me:DOSCMethod>

<!-- <me:DOSCMethod>ClassicalRotors</me:DOSCMethod> -->
</molecule>

</moleculeList>

<reactionList>

<reaction id="R1" active="true" reversible="true">
<reactant>
<molecule ref="CN" role="deficientReactant" />
</reactant>
<reactant>
<molecule ref="CH2O" role="excessReactant" />
</reactant>
<product>
<molecule ref="vdW" role="modelled" />
</product>
<me:excessReactantConc>1E15</me:excessReactantConc>
<me:MCRCMethod xsi:type="me:MesmerILT">
<me:preExponential units="cm3molecule-1s-1" lower="1e-13" upper="9e-10" stepsize="1e-13">6.5836e-11</me:preExponential>
<!-- <me:preExponential units="cm3molecule-1s-1">6.00e-12</me:preExponential> -->
<me:activationEnergy units="kJ/mol">0.0</me:activationEnergy>
<me:TInfinity>30.0</me:TInfinity>
<me:nInfinity lower="-1.4" upper="1.4" stepsize="0.005">-0.097</me:nInfinity>
<!-- <me:nInfinity>-0.5</me:nInfinity> -->
</me:MCRCMethod>
</reaction>

<reaction id="R2" active="true" reversible="true">
<reactant>
<molecule ref="vdW" role="modelled" />
</reactant>
<product>
<molecule ref="HCN" role="sink" />
</product>
<product>
<molecule ref="HCO" role="sink" />
</product>
<me:transitionState>
<molecule ref="TS_HCN" role="transitionState" />
</me:transitionState>
<me:tunneling name="Eckart"/>
<me:MCRCMethod name="RRKM"/>
</reaction>

<!-- <reaction id="R3" active="true" reversible="true"> -->
<!-- <reactant> -->
<!-- <molecule ref="vdW" role="modelled" /> -->
<!-- </reactant> -->
<!-- <product> -->
<!-- <molecule ref="HNC" role="sink" /> -->
<!-- </product> -->
<!-- <product> -->
<!-- <molecule ref="HCO" role="sink" /> -->
<!-- </product> -->
<!-- <me:transitionState> -->
<!-- <molecule ref="TS_HNC" role="transitionState" /> -->
<!-- </me:transitionState> -->
<!-- <me:tunneling name="Eckart"/> -->
<!-- <me:MCRCMethod name="RRKM"/> -->
<!-- </reaction> -->

<!-- <reaction id="R4" active="true" reversible="true"> -->
<!-- <reactant> -->
<!-- <molecule ref="vdW" role="modelled" /> -->
<!-- </reactant> -->
<!-- <product> -->
```

```
				<!-- <molecule ref="Int1" role="modelled" /> -->
			<!-- </product> -->
			<!-- <me:transitionState> -->
				<!-- <molecule ref="TS_chain" role="transitionState" /> -->
			<!-- </me:transitionState> -->
			<!-- <me:tunneling name="Eckart"/> -->
			<!-- <me:MCRCMethod name="RRKM"/> -->
<!-- </reaction> -->

	<!-- <reaction id="R5" active="true" reversible="true"> -->
			<!-- <reactant> -->
				<!-- <molecule ref="Int1" role="modelled" /> -->
			<!-- </reactant> -->
			<!-- <product> -->
				<!-- <molecule ref="NCO" role="sink" /> -->
			<!-- </product> -->
			<!-- <product> -->
				<!-- <molecule ref="CH2_triplet" role="sink" /> -->
			<!-- </product> -->
			<!-- <me:tunneling name="Eckart"/> -->
			<!-- <me:MCRCMethod name="RRKM"/> -->
<!-- </reaction> -->

	<!-- <reaction id="R6" active="true" reversible="true"> -->
			<!-- <reactant> -->
				<!-- <molecule ref="Int1" role="modelled" /> -->
			<!-- </reactant> -->
			<!-- <product> -->
				<!-- <molecule ref="Int2" role="modelled" /> -->
			<!-- </product> -->
			<!-- <me:transitionState> -->
				<!-- <molecule ref="TS1/2" role="transitionState" /> -->
			<!-- </me:transitionState> -->
			<!-- <me:tunneling name="Eckart"/> -->
			<!-- <me:MCRCMethod name="RRKM"/> -->
<!-- </reaction> -->

	<!-- <reaction id="R7" active="true" reversible="true"> -->
			<!-- <reactant> -->
				<!-- <molecule ref="Int2" role="modelled" /> -->
			<!-- </reactant> -->
			<!-- <product> -->
				<!-- <molecule ref="Int3" role="modelled" /> -->
			<!-- </product> -->
			<!-- <me:transitionState> -->
				<!-- <molecule ref="TS2/3" role="transitionState" /> -->
			<!-- </me:transitionState> -->
			<!-- <me:tunneling name="Eckart"/> -->
			<!-- <me:MCRCMethod name="RRKM"/> -->
<!-- </reaction> -->

	<!-- <reaction id="R8" active="true" reversible="true"> -->
			<!-- <reactant> -->
				<!-- <molecule ref="Int3" role="modelled" /> -->
			<!-- </reactant> -->
			<!-- <product> -->
				<!-- <molecule ref="NCCOH" role="sink" /> -->
			<!-- </product> -->
			<!-- <product> -->
				<!-- <molecule ref="H" role="sink" /> -->
			<!-- </product> -->
			<!-- <me:transitionState> -->
				<!-- <molecule ref="TS3/P" role="transitionState" /> -->
			<!-- </me:transitionState> -->
			<!-- <me:tunneling name="Eckart"/> -->
			<!-- <me:MCRCMethod name="RRKM"/> -->
<!-- </reaction> -->
```

```
</reactionList>

        <me:conditions>
        <me:bathGas>N2</me:bathGas>
            <me:PTs>

            <!-- <me:bathGas>N2</me:bathGas> -->
            <!--West 2019-->

<me:PTpair units="PPCC" P="2.91e16" T="70" me:precision="dd"> <me:bathGas>N2</me:bathGas> <me:experimentalRate ref1="CN" ref2="CN"  error=     "5200">13000     </me:experimentalRate> </me:PTpair>
<me:PTpair units="PPCC" P="7.56e16" T="84" me:precision="dd"> <me:bathGas>N2</me:bathGas> <me:experimentalRate ref1="CN" ref2="CN"  error=     "2700">9900      </me:experimentalRate> </me:PTpair>
<me:PTpair units="PPCC" P="4.99e17" T="92" me:precision="dd"> <me:bathGas>N2</me:bathGas> <me:experimentalRate ref1="CN" ref2="CN"  error=     "2900">18000     </me:experimentalRate> </me:PTpair>
<me:PTpair units="PPCC" P="6.80e16" T="103" me:precision="dd"> <me:bathGas>N2</me:bathGas> <me:experimentalRate ref1="CN" ref2="CN"  error="1900">14500      </me:experimentalRate> </me:PTpair>

            <!-- <me:bathGas>Ar</me:bathGas> -->
            <!-- West 2019 -->
<me:PTpair units="PPCC" P="3.24e16" T="32" me:precision="dd"> <me:bathGas>Ar</me:bathGas> <me:experimentalRate ref1="CN" ref2="CN"  error=     "5300         ">35700</me:experimentalRate> </me:PTpair>
<me:PTpair units="PPCC" P="3.24e16" T="32" me:precision="dd"> <me:bathGas>Ar</me:bathGas> <me:experimentalRate ref1="CN" ref2="CN"  error=     "8400         ">46200</me:experimentalRate> </me:PTpair>
<me:PTpair units="PPCC" P="8.36e16" T="40" me:precision="dd"> <me:bathGas>Ar</me:bathGas> <me:experimentalRate ref1="CN" ref2="CN"  error=     "21100        ">22600</me:experimentalRate> </me:PTpair>
<me:PTpair units="PPCC" P="7.04e16" T="53" me:precision="dd"> <me:bathGas>Ar</me:bathGas> <me:experimentalRate ref1="CN" ref2="CN"  error=     "11900        ">31800</me:experimentalRate> </me:PTpair>
<me:PTpair units="PPCC" P="7.58e16" T="56" me:precision="dd"> <me:bathGas>Ar</me:bathGas> <me:experimentalRate ref1="CN" ref2="CN"  error=     "19400        ">15600</me:experimentalRate> </me:PTpair>
<me:PTpair units="PPCC" P="11.18e16" T="70" me:precision="dd"> <me:bathGas>Ar</me:bathGas> <me:experimentalRate ref1="CN" ref2="CN"  error="4900">15100</me:experimentalRate> </me:PTpair>

            <!-- Yu 1993 -->
<me:PTpair units="Torr" P="100" T="297" me:precision="d"> <me:bathGas>Ar</me:bathGas> <me:experimentalRate ref1="CN" ref2="CN"  error=        "1660">16600   </me:experimentalRate> </me:PTpair>
<me:PTpair units="Torr" P="100" T="345" me:precision="d"> <me:bathGas>Ar</me:bathGas> <me:experimentalRate ref1="CN" ref2="CN"  error=        "1720">17200   </me:experimentalRate> </me:PTpair>
<me:PTpair units="Torr" P="100" T="425" me:precision="d"> <me:bathGas>Ar</me:bathGas> <me:experimentalRate ref1="CN" ref2="CN"  error=        "2220">22200   </me:experimentalRate> </me:PTpair>
<me:PTpair units="Torr" P="100" T="528" me:precision="d"> <me:bathGas>Ar</me:bathGas> <me:experimentalRate ref1="CN" ref2="CN"  error=        "2620">26200   </me:experimentalRate> </me:PTpair>
<me:PTpair units="Torr" P="100" T="673" me:precision="d"> <me:bathGas>Ar</me:bathGas> <me:experimentalRate ref1="CN" ref2="CN"  error=        "3990">39900   </me:experimentalRate> </me:PTpair>

            <!-- <me:bathGas>He</me:bathGas> -->
            <!-- Chang 1995 -->
<me:PTpair units="Torr" P="52" T="294" me:precision="d"> <me:bathGas>He</me:bathGas> <me:experimentalRate ref1="CN" ref2="CN"  error=        "1640         ">16400</me:experimentalRate> </me:PTpair>
<me:PTpair units="Torr" P="100" T="294" me:precision="d"> <me:bathGas>He</me:bathGas> <me:experimentalRate ref1="CN" ref2="CN"  error=        "1640         ">16400</me:experimentalRate> </me:PTpair>
<me:PTpair units="Torr" P="155" T="294" me:precision="d"> <me:bathGas>He</me:bathGas> <me:experimentalRate ref1="CN" ref2="CN"  error=        "1640         ">16400</me:experimentalRate> </me:PTpair>
<me:PTpair units="Torr" P="201" T="294" me:precision="d"> <me:bathGas>He</me:bathGas> <me:experimentalRate ref1="CN" ref2="CN"  error=        "1640         ">16400</me:experimentalRate> </me:PTpair>
<me:PTpair units="Torr" P="102.5" T="323" me:precision="d"> <me:bathGas>He</me:bathGas> <me:experimentalRate ref1="CN" ref2="CN"  error="1940">19400         </me:experimentalRate> </me:PTpair>
<me:PTpair units="Torr" P="97.5" T="357" me:precision="d"> <me:bathGas>He</me:bathGas> <me:experimentalRate ref1="CN" ref2="CN"  error=        "2030         ">20300</me:experimentalRate> </me:PTpair>
<me:PTpair units="Torr" P="97.5" T="400" me:precision="d"> <me:bathGas>He</me:bathGas> <me:experimentalRate ref1="CN" ref2="CN"  error=        "2320         ">23200</me:experimentalRate> </me:PTpair>
<me:PTpair units="Torr" P="101" T="455" me:precision="d"> <me:bathGas>He</me:bathGas> <me:experimentalRate ref1="CN" ref2="CN"  error=        "2740         ">27400</me:experimentalRate> </me:PTpair>
<me:PTpair units="Torr" P="95" T="526" me:precision="d"> <me:bathGas>He</me:bathGas> <me:experimentalRate ref1="CN" ref2="CN"  error=        "3110         ">31100</me:experimentalRate> </me:PTpair>
<me:PTpair units="Torr" P="91" T="625" me:precision="d"> <me:bathGas>He</me:bathGas> <me:experimentalRate ref1="CN" ref2="CN"  error=        "3230         ">32300 </me:experimentalRate> </me:PTpair>
<me:PTpair units="Torr" P="91.5" T="769" me:precision="d"> <me:bathGas>He</me:bathGas> <me:experimentalRate ref1="CN" ref2="CN"  error=        "4080         ">40800</me:experimentalRate> </me:PTpair>



<!-- <me:PTpair units="PPCC" P="1e15" T="50"   me:precision="qd"> <me:bathGas>N2</me:bathGas> </me:PTpair> -->

</me:PTs>
  </me:conditions>

        <me:modelParameters>
    <!--Specify grain size directly...-->
    <me:grainSize units="cm-1">30</me:grainSize>
   <!--  <me:energyAboveTheTopHill>10.0</me:energyAboveTheTopHill> -->
              <me:automaticallySetMaxEne>1.0e-15</me:automaticallySetMaxEne>
  </me:modelParameters>

        <me:control>
        <me:calcMethod xsi:type="me:marquardt">
   <me:MarquardtIterations>12</me:MarquardtIterations>
   <me:MarquardtTolerance>1e-9</me:MarquardtTolerance>
   <me:MarquardtDerivDelta>2.e-02</me:MarquardtDerivDelta>
  </me:calcMethod>
  <me:testDOS />
  <me:printSpeciesProfile />
  <!--<me:testMicroRates />-->
  <me:testRateConstant />
  <me:printGrainDOS />
  <!--<me:printCellDOS />-->
  <!--<me:printReactionOperatorColumnSums />-->
  <!--<me:printTunnellingCoefficients />-->
  <me:printGrainkfE />
  <!--<me:printGrainBoltzmann />-->
  <me:printGrainkbE />
  <me:eigenvalues>15</me:eigenvalues>
  <me:diagramEnergyOffset ref="R1">0</me:diagramEnergyOffset>
        <me:MaximumEvolutionTime>1</me:MaximumEvolutionTime>
```

</me:control>

<!-- <me:conditions> -->
<!-- <me:bathGas>Ar</me:bathGas> -->
<!-- <me:PTs> -->

<!-- <me:PTpair units="PPCC" P="1.00e13" T="20." precision="qd" default="true" bathGas="Ar"> </me:PTpair> -->

<!-- </me:PTs> -->
<!-- </me:conditions> -->

<!-- Error estimates for above condiditons -->
<!-- <me:control> -->
<!-- <me:calcMethod xsi:type="me:ErrorPropagation"/> -->
<!-- <me:errorPropagationSamples>4096</me:errorPropagationSamples> -->
<!-- </me:control> -->
